# Life expectancy associated with different ages at diagnosis of diabetes: 23 million person-years of observation

**DOI:** 10.1016/S2213-8587(23)00223-1

**Published:** 2023-09-11

**Authors:** Emerging Risk Factors Collaboration, Stephen Kaptoge, Sreenivasa Rao Kondapally Seshasai, Luanluan Sun, Matthew Walker, Thomas Bolton, Sarah Spackman, Feven Ataklte, Peter Willeit, Steven Bell, Steven Burgess, Lisa Pennells, Servet Altay, Gerd Assmann, Yoav Ben-Shlomo, Lyle G Best, Cecilia Björkelund, Dan G Blazer, Hermann Brenner, Eric J Brunner, Gilles R Dagenais, Jackie A Cooper, Cyrus Cooper, Carlos J Crespo, Mary Cushman, Ralph B D’Agostino Sr, Makoto Daimon, Lori B Daniels, Rachel Dankner, Karina W Davidson, Renate T de Jongh, Chiara Donfrancesco, Pierre Ducimetiere, Petra J M Elders, Gunnar Engström, Ian Ford, John Gallacher, Stephan J L Bakker, Uri Goldbourt, Agustin Gómez de la Cámara, Sameline Grimsgaard, Vilmundur Gudnason, Per-Olof Hansson, Hironori Imano, J Wouter Jukema, Christopher Kabrhel, Jussi Kauhanen, Maryam Kavousi, Stefan Kiechl, Matthew W Knuiman, Daan Kromhout, Harlan M Krumholz, Lewis H Kuller, Tiina Laatikainen, Debbie A Lawlor, Haakon E Meyer, Kenneth Mukamal, Paul J Nietert, Toshiharu Ninomiya, Dorothea Nitsch, Børge G Nordestgaard, Luigi Palmieri, Jackie F Price, Paul M Ridker, Qi Sun, Annika Rosengren, Ronan Roussel, Masaru Sakurai, Veikko Salomaa, Ben Schöttker, Jonathan E Shaw, Timo E Strandberg, Johan Sundström, Hanna Tolonen, Aage Tverdal, WM Monique Verschuren, Henry Völzke, Lynne Wagenknecht, Robert B Wallace, S Goya Wannamethee, Nicholas J Wareham, Sylvia Wassertheil-Smoller, Kazumasa Yamagishi, Bu B Yeap, Seamus Harrison, Michael Inouye, Simon Griffin, Adam S Butterworth, Angela M Wood, Simon G Thompson, Naveed Sattar, John Danesh, Emanuele Di Angelantonio

**Affiliations:** 1 BHF Cardiovascular Epidemiology Unit, Department of Public Health and Primary Care, University of Cambridge, Cambridge, UK; 2 Victor Phillip Dahdaleh Heart and Lung Research Institute, University of Cambridge, Cambridge, UK; 3 St George’s University, London, UK; 4 BHF Data Science Centre, Health Data Research UK, London, UK; 5 Department of Internal Medicine Boston Medical Center and Boston University School of Medicine Boston MA; 6 Clinical Epidemiology Team, Medical University of Innsbruck, Austria; 7 Department of Clinical Neurosciences, University of Cambridge, Cambridge, UK; 8 BHF Centre of Research Excellence, School of Clinical Medicine, Addenbrooke’s Hospital, University of Cambridge, UK; 9 Medical Research Council Biostatistics Unit, University of Cambridge, UK; 10 Department of Cardiology, Trakya University School of Medicine, Erdine, Turkey; 11 Assmann Foundation for Prevention, Münster, Germany; 12 Population Health Sciences, University of Bristol, Bristol, UK; 13 Missouri Breaks Industries Research, Inc., Eagle Butte, SD, USA; 14 Institute of Medicine, Sahlgrenska Academy, University of Gothenburg, Gothenburg, Sweden; 15 School of Medicine, Duke University, Durham, NC, USA; 16 Division of Clinical Epidemiology and Aging Research, German Cancer Research Center, Heidelberg, Germany; 17 Network Aging Research, Heidelberg University, Heidelberg, Germany; 18 Department of Epidemiology and Public Health University College London, London, UK; 19 Quebec Heart and Lung Institute, Quebec, QC, Canada; 20 William Harvey Research Institute, NIHR Barts Biomedical Research Centre, Queen Mary University of London, London, UK; 21 MRC Lifecourse Epidemiology Centre University of Southampton, Southampton, UK; 22 Oregon Health and Science University and Portland State University Joint School of Public Health, Portland, Oregon; 23 Larner College of Medicine, The University of Vermont, Burlington, VT, USA; 24 Mathematics and Statistics Department, Boston University, Boston, MA, USA; 25 Global Center of Excellence Program Study Group, Yamagata University Faculty of Medicine, Yamagata, Japan; 26 Department of Endocrinology and Metabolism, Hirosaki University Graduate School of Medicine, Aomori, Japan; 27 UCSD Division of Cardiovascular Medicine, Sulpizio Cardiovascular Center, La Jolla, CA, USA; 28 The Gertner Institute for Epidemiology and Health Policy Research, Sheba Medical Center, Tel Hashomer, Israel; 29 School of Public Health, Department of Epidemiology and Preventive Medicine, Tel Aviv University, Ramat Aviv, Tel Aviv, Israel; 30 The Feinstein Institute for Medical Research, Northwell Health, Manhasset, NY, USA; 31 Amsterdam University Medical Centers, VUMC, Amsterdam, the Netherlands; 32 Department of Cardiovascular, Endocrine-metabolic Diseases and Aging, Istituto Superiore di Sanità, Rome, Italy; 33 Inserm - Université Paris Sud - CESP Villejuif, France; 34 Department of General Practice, Amsterdam UMC, Vrije Universiteit, Amsterdam Public Health research institute, Amsterdam, the Netherlands; 35 Department of Clinical Sciences Malmö, Lund University, Malmö, Sweden; 36 Institute of Health & Wellbeing, University of Glasgow, Glasgow, UK; 37 Department of Psychiatry, University of Oxford, Oxford, UK; 38 Department of Internal Medicine, University Medical Centre Groningen, University of Groningen, Groningen, Netherlands; 39 Department of Epidemiology and Preventive Medicine, School of Public Health, Sackler Faculty of Medicine, Tel-Aviv University, Tel-Aviv, Israel; 40 12 Octubre Hospital Research Institute, Madrid, Spain; 41 Department of Community Medicine, UiT The Arctic University of Norway, Tromsø, Norway; 42 Faculty of Medicine, University of Iceland, Reykjavik, Iceland; 43 Icelandic Heart Association, Kopavogur, Iceland; 44 Region Västra Götaland, Sahlgrenska University Hospital, Department of Medicine Geriatrics and Emergency Medicine/Östra, Gothenburg, Sweden; 45 Institute of Medicine, Department of Molecular and Clinical Medicine, Sahlgrenska Academy, Gothenburg University, Gothenburg, Sweden; 46 Public Health, Osaka University Graduate School of Medicine, Suita, Japan; 47 Department of Cardiology, Leiden University Medical Center, the Netherlands; 48 Einthoven Laboratory for Experimental Vascular Medicine, LUMC, Leiden, the Netherlands; 49 Netherlands Heart Institute, Utrecht, the Netherlands; 50 Center for Vascular Emergencies, Department of Emergency Medicine, Massachusetts General Hospital, Harvard Medical School, Boston, MA, USA; 51 University of Eastern Finland (UEF), Kuopio, Finland; 52 Department of Epidemiology, Erasmus MC, University Medical Center Rotterdam, Rotterdam, the Netherlands; 53 Medical University Innsbruck, Innsbruck, Austria; 54 School of Population and Global Health, The University of Western Australia, Crawley, WA, Australia; 55 University of Groningen, University Medical Center Groningen, Department of Epidemiology, Groningen, The Netherlands; 56 Center for Outcomes Research and Evaluation, Yale-New Haven Hospital, New Haven, CT, USA; 57 Department of Health Policy and Management, Yale School of Public Health, New Haven, CT, USA; 58 Section of Cardiovascular Medicine, Department of Internal Medicine, Yale School of Medicine, New Haven, CT, USA; 59 Department of Epidemiology, University of Pittsburgh, Pittsburgh, PA, USA; 60 National Institute for Health and Welfare, Helsinki, Finland; 61 MRC Integrative Epidemiology Unit at the University of Bristol, UK; 62 Population Health Science, Bristol Medical School, University of Bristol, UK; 63 Norwegian Institute of Public Health, Oslo, Norway; 64 Beth Israel Deaconess Medical Centre, Harvard Medical School, Harvard University, Boston, MA, USA; 65 Department of Public Health Sciences, Medical University of South Carolina, Charleston, SC, USA; 66 Department of Epidemiology and Public Health, Graduate School of Medical Sciences, Kyushu University, Fukuoka, Japan; 67 London School of Hygiene & Tropical Medicine, London, UK; 68 Department of Clinical Biochemistry and the Copenhagen General Population Study, Herlev and Gentofte Hospital, Copenhagen University Hospital, Denmark; 69 The Copenhagen City Heart Study, Frederiksberg Hospital, Copenhagen University Hospital, Denmark; 70 Department of Clinical Medicine, Faculty of Health and Medical Sciences, University of Copenhagen, Denmark; 71 Department of Cardiovascular, Dysmetabolic and Ageing-Associated Diseases, Istituto Superiore di Sanità, Rome, Italy; 72 Usher Institute, University of Edinburgh, Edinburgh, UK; 73 Brigham & Women’s Hospital, Harvard Medical School, Harvard University, Boston, MA, USA; 74 Sahlgrenska University Hospital and Östra Hospital, Göteborg, Sweden; 75 Chef de Service Endocrinologie Diabétologie Nutrition, Departement Hospitalo-Universitaire FIRE Groupe Hospitalier Bichat - Claude Bernard, Paris; 76 Department of Social and Environmental Medicine, Kanazawa Medical University; 77 National Institute for Health and Welfare, Helsinki, Finland; 78 Division of Clinical Epidemiology and Aging Research, German Cancer Research Center (DKFZ), Heidelberg, Germany; 79 Network Aging Research, University of Heidelberg, Heidelberg, Germany; 80 Clinical Diabetes and Epidemiology, Baker Heart and Diabetes Institute, Melbourne, VIC, Australia; 81 University of Helsinki and Helsinki University Hospital, Helsinki, Finland; 82 University of Oulu, Center for Life Course Health Research, Oulu, Finland; 83 Department of Medical Sciences, Uppsala University, Uppsala, Sweden; 84 Department of Public Health Solutions, National Institute for Health and Welfare (THL), Helsinki, Finland; 85 Norwegian Institute of Public Health, Centre for Fertility and Health, Oslo, Norway; 86 National Institute for Public Health and the Environment (RIVM), Bilthoven, the Netherlands; 87 Julius Center for Health Sciences and Primary Care, University Medical Center Utrecht, Utrecht University, Utrecht, the Netherlands; 88 Universitätsmedizin Greifswald, Institut für Community Medicine, Abteilung SHIP/ Klinisch-Epidemiologische Forschung, Greifswald; 89 Wake Forest School of Medicine, Wake Forest University, Winston-Salem, NC, USA; 90 College of Public Health, University of Iowa, Iowa, IA, USA; 91 Department of Primary Care and Population Health, University College London, London, UK; 92 MRC Epidemiology Unit, School of Clinical Medicine, University of Cambridge, Cambridge, UK; 93 Department of Epidemiology and Population Health, Albert Einstein College of Medicine, Bronx, NY, USA; 94 Department of Public Health Medicine, Faculty of Medicine, and Health Services Research and Development Center, University of Tsukuba, Tsukuba, Japan; 95 Medical School, The University of Western Australia, and Department of Endocrinology and Diabetes, Fiona Stanley Hospital, Perth, Western Australia, Australia; 96 Baker Heart and Diabetes Institute, Melbourne, VIC, Australia; 97 Health Data Research UK Cambridge, Wellcome Genome Campus and University of Cambridge, UK; 98 The Alan Turing Institute, London, UK; 99 Primary Care Unit, Department of Public Health and Primary Care, University of Cambridge, Cambridge, UK; 100 NIHR Blood and Transplant Research Unit in Donor Health and Behaviour, University of Cambridge, UK; 101 Cambridge Centre for AI in Medicine, Cambridge, UK; 102 Institute of Cardiovascular & Medical Sciences, University of Glasgow, Glasgow, UK; 103 Department of Human Genetics, Wellcome Sanger Institute, Hinxton, UK; 104 Health Data Science Centre, Human Technopole, Milan, Italy

## Abstract

**Background:**

The prevalence of type 2 diabetes is increasing rapidly, particularly among younger adults. It is estimated that people with diabetes die, on average, six years earlier than people without diabetes. Our aim was to provide reliable estimates of the associations of age at diagnosis of diabetes with all-cause and cause-specific mortality and reductions in life expectancy.

**Methods:**

We conducted a combined analysis of individual-participant-data from two large-scale data sources in 19 high-income countries, Emerging Risk Factors Collaboration (96 cohorts, baseline years 1961-2020, latest follow up years 1980-2020) and UK Biobank (baseline year 2006, latest follow up year 2020). We calculated age- and sex-adjusted hazard ratios (HRs) for all-cause mortality according to age at diagnosis of diabetes in 1,515,718 participants, in whom deaths were recorded during 23.1 million person-years of follow-up. We estimated cumulative survival by applying age-specific HRs to contemporary age-specific death rates in US and Europe.

**Findings:**

We observed a log-linear dose-response association between earlier age at diagnosis of diabetes and higher risk of all-cause mortality as compared to concurrent participants without diabetes. HRs were 2.69 (95% CI: 2.43-2.97) at 30-39 years, 2.26 (2.08-2.45) at 40-49 years, 1.84 (1.72-1.97) at 50-59 years, 1.57 (1.47-1.67) at 60-69 years, and 1.39 (1.25-1.51) at age ≥70 years. HRs per decade earlier diagnosis were similar for men and women. Using US death rates, a 50-year-old with diabetes, diagnosed at age 30, 40, or 50 years died on average 14, 10, or 6 years earlier, respectively, than an individual without diabetes. Corresponding estimates were 13, 9, or 5 years earlier using EU death rates.

**Interpretation:**

Every decade of earlier diagnosis of diabetes was associated with about three to four years of lower life expectancy, highlighting the potential value of early interventions that delay or prevent diabetes.

**Funding:**

BHF, MRC, NIHR, HDRUK

## Introduction

The prevalence of type 2 diabetes is rising globally, driven mainly by behavioural and societal factors related to obesity, nutrition and physical activity.^1-3^ In 2021, 537 million adults were estimated to have diabetes worldwide, with an increasing numbers diagnosed at younger ages.^3,4^

Previous estimates have suggested that adults with type 2 diabetes die, on average, six years earlier than counterparts without diabetes.^5-7^ There is uncertainty, however, about how this average reduction in life expectancy varies according to age at diagnosis.^8-19^ Valid characterization of this association requires prospective comparison of outcomes within the same cohorts of people with diabetes diagnosed at varying ages. However, few population cohorts have had sufficient statistical power, detail, and duration of follow-up to enable secure estimation.^20-25^ Moreover, existing modelling studies have only considered diabetes as a binary condition in estimating the impact on life expectancy using state-transition models and life tables that rely on inputs from aggregated data.^7,26-29^ Thus few of published studies have directly analysed the associations of age at diagnosis of diabetes *per se* with mortality and life expectancy.

Here we aim to provide reliable estimates of the associations of age at diagnosis of diabetes with all-cause and cause-specific mortality and reductions in life expectancy in high-income countries. We analysed individual records from 97 long-term prospective cohorts involving 1,515,718 participants followed-up for a total of 23.1 million person-years.

## Methods

### Study design, data sources, and participants

We conducted a combined analysis of individual-participant data from two large-scale data sources, each constituting prospective population cohort studies with information on age at diagnosis of diabetes (appendix pp 2-3 and p 24). First, the Emerging Risk Factors Collaboration (ERFC) is a collaboration of prospective cohort studies with information about a variety of risk factors, cardiovascular disease outcomes, and mortality.^30^ Prospective cohort studies contributing to the ERFC were included in this analysis if they met all of the following criteria: had recruited participants on the basis of informed consent; did not select participants on the basis of having previous chronic disease (including cardiovascular disease and diabetes); had recorded information on diabetes status, and age at diagnosis of diabetes; had recorded cause-specific deaths; and had accrued more than 1 year of follow-up. The second data source was the UK Biobank (UKBB), a single large prospective study in which participants were recruited from 22 centres throughout the UK.^31^ After giving consent, participants provided biological samples and completed a touch-screen questionnaire, a computer-assisted interview, and a physical examination (appendix p 26). Data from participants in the UKBB have been linked with death records of the UK Office for National Statistics through National Health Service identification numbers. For all studies, written informed consent was obtained from participants and approval was obtained from relevant ethics committees.

We ascertained baseline diabetes status on the basis of self-report information, medical records, medication usage, or a combination of these factors (appendix p 4).^5,32^ To calculate age at diagnosis of diabetes, we used information recorded at the “baseline” enrolment survey in prospective cohort studies, supplemented, when available, by information on new-onset incident type 2 diabetes recorded during follow-up (appendix p 27). For 37,513 of 47,404 (79%) new-onset incident cases, age at diagnosis of diabetes was calculated using date of diagnosis provided by the contributing cohorts. For the remaining 9891 (21%) of the new-onset incident cases of diabetes, for whom information was provided as diabetes status (yes/no) at date-stamped resurveys, we estimated the age at diagnosis as the participant’s age at the midpoint of two consecutive surveys in which the participant developed diabetes (appendix pp 2-3). We also computed an accuracy indicator as half-width of the time interval between the two surveys, and the average was ±2.4 (SD 0.9) years (appendix pp 2-3).

We classified mortality according to the primary cause (or, in its absence, the underlying cause) on the basis of coding from the *International Classification of Diseases*, revisions 8 through 10, to at least 3 digits, or according to study-specific classification systems. Classification of deaths was based on death certificates, supplemented in 76 studies by medical records, findings on autopsy, and other sources in the ERFC. The date of latest mortality follow-up was October 2014 in the ERFC and November 2020 in UKBB.

### Statistical analysis

To be eligible for the analysis, participants had to have information recorded about their history of diabetes plus age and sex. To focus analysis on individuals with type 2 diabetes, we excluded 3695 participants diagnosed with diabetes at age <30 years, who would be more likely to have type 1 diabetes. To assess “dose-response” relationships, we categorized participants according to their history of diabetes (yes vs no) and their age at diagnosis into 10-year groups: i.e., 30 to <40 years, 40 to <50 years, 50 to <60 years, 60 to <70 years, and ≥70 years. We also assessed the continuous shape of associations using fractional polynomials. We then assessed adjusted associations, guided by the dose-response analyses results and prior evidence for other continuous covariates. The primary outcome was all-cause mortality, with additional outcomes including deaths from vascular disease, cancer, and nonvascular conditions not attributed to cancer (appendix pp 5-6). Hazard ratios (HRs) for age at diagnosis of diabetes were calculated separately within each study using time-dependent Cox proportional hazards regression models (i.e., allowing diabetes status, age at diagnosis, and other covariates to change during follow up, when reassessed). The timescale for the survival analysis was duration (in years) since entry to the study at baseline. Participants were included in analyses of mortality outcomes irrespective of previous non-fatal events. For each specific cause of death, participants’ data were censored if a participant was lost to follow-up, died from other causes, or reached the end of follow-up period. HRs calculated in this manner for each cause of death are aetiologically interpretable and provide reliable assessments of the marginal cause-specific associations, including in the case of competing risks with low to moderate correlations of failure times, that would be typical of most practical circumstances.^33-35^ Sensitivity analyses were conducted for cause-specific mortality considering death from other causes as competing risks using the Fine and Gray regression model. Study-specific estimates (i.e. log HRs) were then pooled across studies by multivariate random-effects meta-analysis due to expected heterogeneity with diverse data sources analysed.^36^ To avoid model overfitting, studies with fewer than 10 deaths for any outcome (i.e. all cause and cause specific death) were excluded from the main analyses for relevant outcomes. Further sensitivity analyses excluded studies with fewer than 80 deaths (i.e. applying stricter 10 events per variable rule at the study level). The proportional hazards assumption, assessed by meta-analysis of study-specific interaction of coded exposure variable (indicators or continuous) and the survival analysis time in years, was met (P>0.05).

Because the principal objective of our study was to estimate reductions in life expectancy according to age at diagnosis of diabetes, the main analysis calculated HRs stratified by sex and adjusted for age only. A secondary objective was to explore the extent to which the age-specific relevance of diabetes could be accounted by other known factors associated with mortality risk. Hence, HRs were sequentially adjusted for several variables recorded after diagnosis of diabetes, including smoking status, body-mass index (BMI), systolic blood pressure, total cholesterol, measures of glycemia, measures of renal function, measures of inflammation, level of education, and self-reported use of medications. These variables were selected considering subject matter knowledge and data availability. The order of sequential adjustment reflected prioritisation of a variable as a confounder, mediator, or indicator of severity of diabetes, consistent with principles of the modified disjunctive cause criterion reasoning.^37^ We investigated effect modification with tests for interaction for individual characteristics (age, sex, smoking, history of CVD) and by meta-regression of study-specific log HRs (i.e. outcome) on study-level characteristics (diabetes diagnosis information available, median year of baseline, median year of follow up) assuming normal error terms^36^ and using a 0.001 significance threshold to make some allowance for multiple testing (i.e. 0.01/7 for seven interactions assessed at 0.01 nominal significance each). Between-study heterogeneity of log HRs was assessed by the *I*^2^ statistic.^38^

Appendix (p28) provides details of the methods used to estimate reductions in life expectancy by age at diagnosis of diabetes. Briefly, estimates of cumulative survival from 40 years of age onward according to age at diagnosis of diabetes were calculated by applying the HRs for cause-specific mortality (specific to age at risk and sex) to respective mortality rates obtained from the detailed mortality component of the US Centers for Disease Control and Prevention’s CDC WONDER database,^39^ which recorded 2.7 million deaths among more than 320 million individuals during year 2015.^9,13^ This method does not rely on the survival estimates from the cohort data; instead, it makes inferences by estimating age-at-risk specific HRs from the cohort data, which are then combined with external population age-specific mortality rates.^11^ Supplementary analyses used European Union (EU) death rates during 2015. Analyses involved Stata version 15.1 (StataCorp), 2-sided P-values, and used a significance level of P<0.05 unless stated otherwise.

### Role of the funding source

The funders of the study had no role in study design, data collection, data analysis, data interpretation, or writing of the report.

## Results

A total of 1,515,718 participants from 97 prospective cohorts had sufficient information for inclusion in this analysis (comprising 1,017,695 participants in 96 ERFC cohorts and 498,023 participants in UKBB, Table 1 and appendix pp 2-3). In ERFC the median year of recruitment was 1990 (range 1961-2020) and the median of year of latest follow up was 2015 (range 1980-2020). Corresponding values in UKBB were 2009 and 2020. In the ERFC, the large majority of participants were enrolled in Europe (50%) or North America (42%). Overall, the mean (SD) age of participants at baseline was 55.0 (9.2) years and 690,596 participants (46%) were male. In ERFC, age at diagnosis of prevalent diabetes was available for 23,335 participants (57% of 41,160 participants who had prevalent diabetes) and a further 45,585 participants had diabetes diagnosed during follow-up (i.e., new-onset disease). In UKBB, age at diagnosis of prevalent diabetes was available for 24,981 participants (98% of 25,416 participants who had prevalent diabetes), and a further 1819 participants had diabetes diagnosed during follow-up. The mean (SD) age at diagnosis of diabetes was 54 (9) years for the prevalent cases and 65 (9) years for the incident cases. Over a median follow-up of 12.5 years (5^th^-95^th^ percentile: 5.0-32.1 years; 23.1 million person-years at risk), there were 246,670 deaths recorded, including 84,443 deaths due to vascular diseases, 85,014 deaths due to cancer, and 61,516 deaths due to non-vascular, non-cancer causes (appendix p 5).

**Figure 1:**
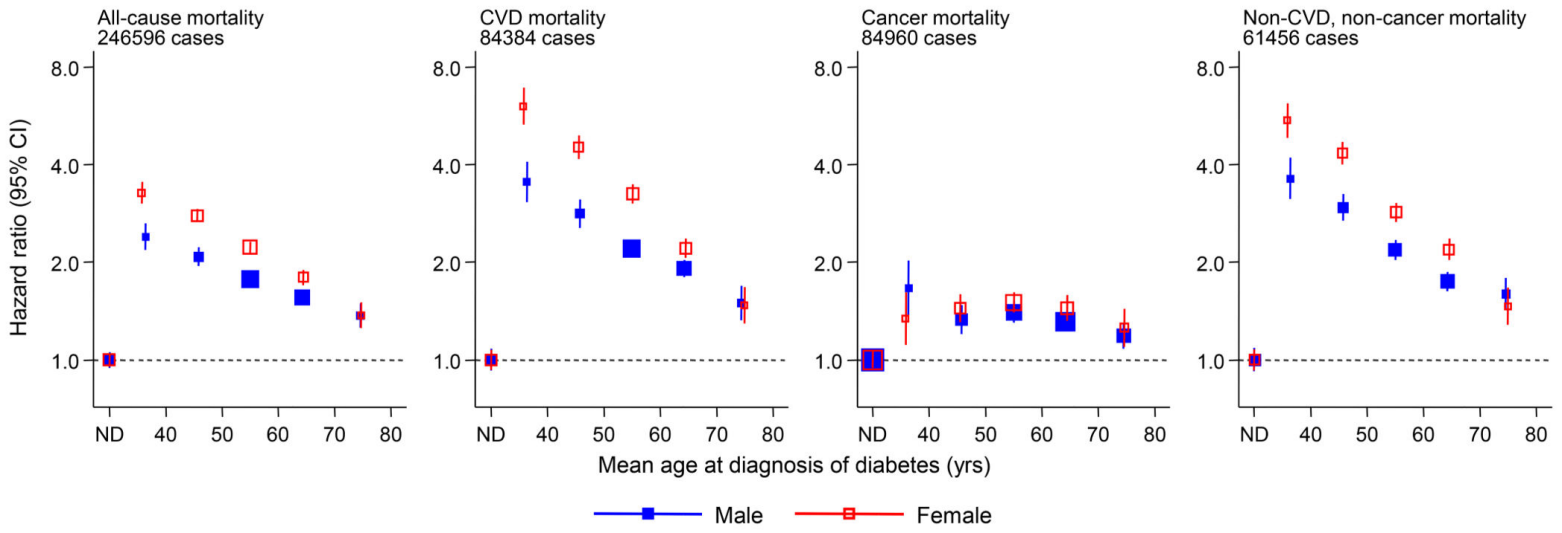
Sex-specific hazard ratios for all-cause and cause-specific mortality according to age at diagnosis of diabetes. ND, No diabetes. The 6 categories of age at diagnosis correspond to: ND, 30 to <40 yrs, 40 to <50 yrs, 50 to <60 yrs, 60 to <70yrs, and ≥70 yrs. Hazard ratios adjusted for age. The reference category is no diabetes. Studies with fewer than 10 events of any outcome were excluded from the analysis of that outcome. Sizes of the boxes are proportional to the inverse of the variance of the log-transformed hazard ratios. Vertical lines represent 95% CIs.

In analyses adjusted for age we observed a log-linear dose-response association between earlier age at diagnosis of diabetes and higher risk of all-cause, CVD and “non-CVD, non-cancer” mortality for each sex (Figure 1), with broadly similar findings in combined analyses adjusted for sex and continuous modelling with fractional polynomials (appendix p 13). Further adjusted analyses used data from 92 cohorts and 1,132,277 participants with complete information on the following factors: age at diagnosis of diabetes, age, sex, smoking, body mass index, systolic blood pressure and total cholesterol. Compared to participants without a history of diabetes, HRs for all-cause mortality, adjusted for age and sex only, were: 2.69 (95% CI: 2.43-2.97) for those diagnosed at 30-39 years, 2.26 (2.08-2.45) at 40-49 years, 1.84 (1.72-1.97) at 50-59 years, 1.57 (1.47-1.67) at 60-69 years, and 1.39 (1.29-1.51) at age ≥70 years (Table 2). For participants diagnosed with diabetes at age 30-39 years, HRs were 4.20 (3.57-4.94) for vascular mortality, 1.55 (1.30-1.85) for cancer mortality, and 3.99 (3.50-4.55) for non-vascular non-cancer mortality (mainly comprising diseases of the respiratory system, nervous system, infections, and external causes). Across all ages, HRs per decade of earlier diagnosis of diabetes were 1.14 (1.08-1.19) for all-cause mortality, 1.19 (1.11-1.27) for vascular mortality, 0.95 (0.88-1.02) for cancer mortality, and 1.18 (1.10-1.27) for non-vascular non-cancer mortality (Table 2).

HRs for all-cause mortality changed little after additional adjustment for other risk factors (Table 2). However, HRs were attenuated substantially after further adjustment for measures of glycemia (i.e., fasting glucose or HbA1c), a pattern also observed for the cause-specific mortality that we studied (appendix p 7). There was little change in HRs after adjustment for measures of renal function (i.e., estimated glomerular filtration rate), inflammation (i.e., C-reactive protein), or lipids (i.e., non-HDL, HDL, triglycerides; appendix p 7).

Broadly similar HRs to those noted above were observed in sensitivity analyses that compared results by: diabetes defined using prevalent disease, incident disease, or both; participant characteristics (eg, smoking status; appendix p 14). HRs differed somewhat by calendar time of study enrolment, or follow-up period (appendix pp 14-15), and by data source (ie, ERFC and UKBB; appendix p 16). Tests for interactions on additive scale were generally confirmative of positive interactions of female sex, current smoking, older age, and history of CVD with diabetes status categorised according to age at diagnosis (appendix p 8). Associations were broadly similar also in analyses that estimated HRs for all-cause and cause-specific mortality according to duration of diabetes (ie, time since diagnosis), rather than age at diagnosis (appendix pp 9-10 and 17). Supplementary analyses according to detailed components of non-CVD mortality suggested broadly similar associations for cancer mortality components, but potentially notable variations in the magnitude of associations for non-CVD non-cancer mortality components (appendix p 11), such as HRs per decade earlier diagnosis of diabetes of 1.46 (1.16, 1.84) for renal disease mortality, 1.28 (1.07, 1.53) for infection related mortality, 1.21 (1.04, 1.42) for external causes of mortality, 1.20 (1.03, 1.40) for digestive system disease mortality, and 1.07 (0.96, 1.19) for respiratory system disease mortality, among others. Results of cause-specific mortality were broadly similar when using competing risks adjusted analyses (appendix p 12). Loss to follow up was less than 10% in majority of studies but the percentage of right censored participants and cause-specific deaths somewhat varied across cohorts (appendix pp 18-19). Sensitivity analyses excluding studies with fewer than 80 cause-specific deaths showed similar findings as in the main analyses excluding studies with fewer than 10 deaths (appendix p 12).

### Life expectancy

**Figure 2:**
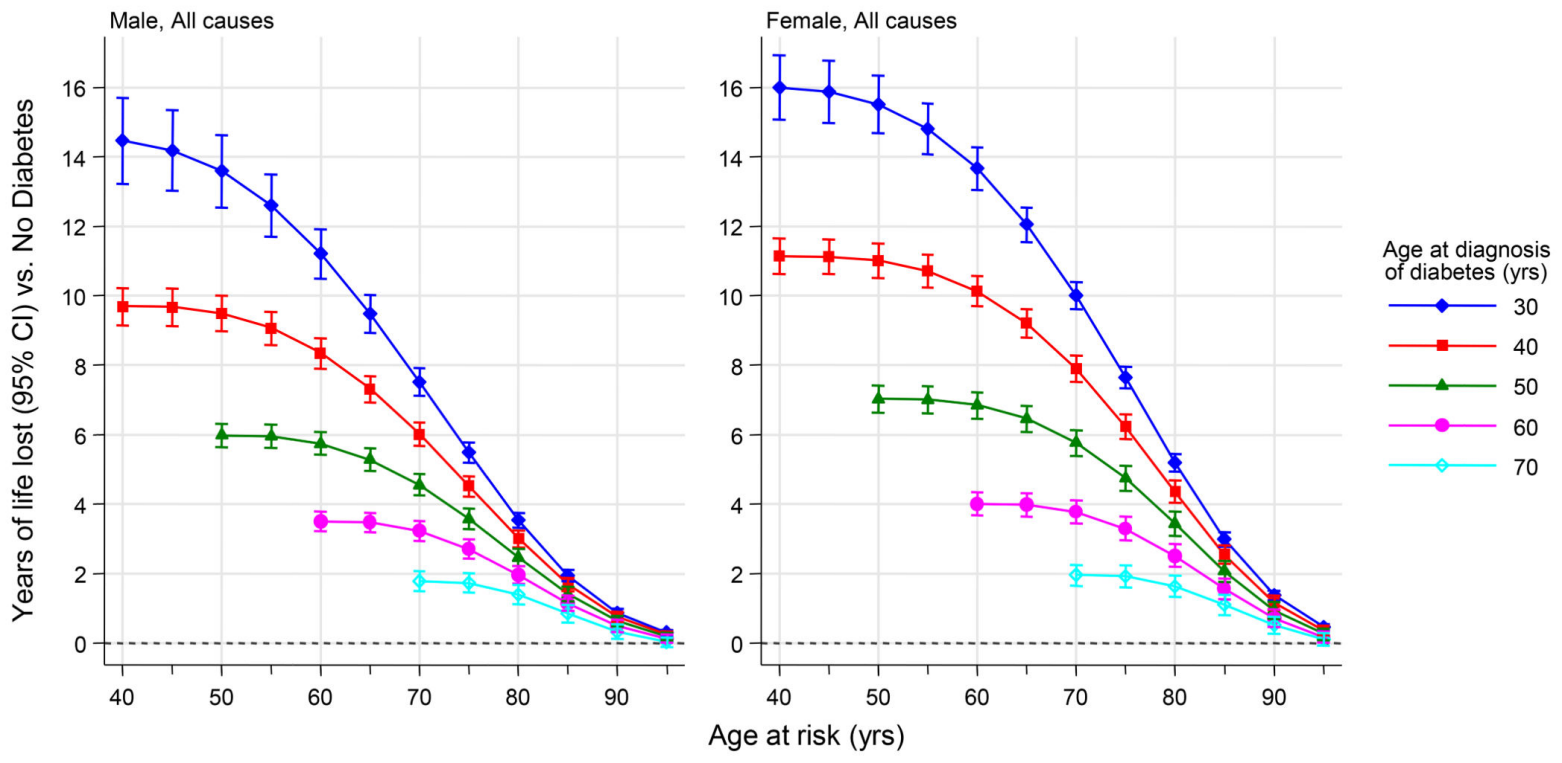
Estimated years of life lost by age at diagnosis of diabetes compared with those without diabetes The estimates of cumulative survival from 40 years of age onwards according to age at diagnosis of diabetes were calculated by applying hazard ratios (specific to age at risk) for all-cause mortality associated with age at diagnosis of diabetes to US 2015 death rates at the age of 40 years or older.

Compared to absence of diabetes at different attained ages, earlier age at diagnosis of diabetes was associated with greater reductions in life expectancy using US death rates (Figure 2). For example, at age 50 years, individuals with diabetes diagnosed at ages 30, 40, and 50 years on average died about 14, 10, and 6 years earlier, respectively, than individuals without diabetes (Figure 2). These estimates were slightly higher in women (16, 11, and 7 years, respectively) than men (14, 9, and 5 years, respectively; Figure 2). Depending on age and sex, vascular deaths accounted for about 30% to 45% of the reduction in life expectancy associated with diabetes, with the remaining percentage being largely due to non-CVD, non-cancer deaths (appendix p 20). Findings were broadly similar in analyses using EU 2015 death rates, with corresponding estimates being about 13, 9, or 5 years earlier death on average (appendix p 21). In supplementary analyses including people with diabetes diagnosed before age 30 years, we found similar patterns in estimated reductions in life expectancy with highest estimated reductions in those diagnosed in childhood and more notably higher in women than men (appendix pp 22-23). At age 50 years, the estimates corresponded to about two to three years reduction per decade earlier diagnosis.

## Discussion

We analysed over 23 million person-years of longitudinal data from population cohorts in 19 high-income countries. We found a steep log-linear dose-response association between earlier age at diagnosis of diabetes and higher risk of all-cause mortality. Overall, every decade of earlier diagnosis of diabetes was associated with about four years of reduced life expectancy. Our modelling has suggested that, for individuals surviving to age 50, those with diabetes diagnosed at age 30, 40, and 50 years died, on average, 14, 10, and 6 years earlier, respectively, than individuals without diabetes. The strongest associations of earlier age at diagnosis of diabetes were for vascular causes of death (e.g., myocardial infarction and stroke), and other non-neoplastic causes of death, mainly respiratory, neurological, and infectious diseases as well as external causes. Our estimates of reduced life expectancy associated with diabetes were somewhat greater for women than men. A key implication of our results is the high priority that should be given to developing and implementing interventions that prevent or delay onset of diabetes, especially as the prevalence of diabetes among younger adults is rising globally^3^.

Our observation of higher HRs for mortality with earlier age at diagnosis of diabetes suggests that the relative impact of diabetes is greatest at ages when the underlying risk of mortality in the general population is lowest. The same phenomenon has been previously observed for other vascular risk factors, including blood pressure^40^ and LDL-cholesterol^41^. Conversely, in older adults, in whom the underlying mortality risk is high, the proportional relevance of diabetes is smaller. It has been previously suggested that individuals who develop type 2 diabetes at younger ages may have more aggressive “phenotypes”^42^ (characterised by higher BMI, blood pressure, and pro-atherogenic lipids,^43^ as well as faster deterioration in glycemic control^24,44^) than individuals who develop diabetes at older ages, potentially leading to premature mortality.^45^ Our findings are consistent with this hypothesis, suggesting the large excess mortality associated with diabetes at younger ages may, in part, reflect cumulative exposure to worsened metabolic profiles. Furthermore, we observed substantial attenuation of excess mortality associated with diabetes after adjustment for glycemic markers, suggesting that early detection of diabetes by screening and intensive glucose management are relevant to prevention of long-term complications in adults with type 2 diabetes.^46,47,48^

Our study had several strengths and it is distinctive and complimentary to previously reported studies.^7-19,26-29^ Our focus on age at diagnosis of diabetes avoided inherent difficulties in defining age at onset of diabetes (which may require near continuous assessment of glycemic status),^49^ and in defining duration of diabetes (which may be confounded by the timing and duration of participants’ entry into prospective cohort studies). Furthermore, our study estimated age at diagnosis of diabetes using information from people diagnosed with prevalent diabetes as well as those diagnosed with incident diabetes. Our study’s access to individual-participant data avoided limitations of previous literature-based reviews, allowing extensive sensitivity analyses to assess potential sources of heterogeneity and interactions according to study-level (including calendar time) and individual-level characteristics. Our estimation of reductions in life expectancy relied on age-specific HRs directly estimated from individual-level data and applied to contemporary population-specific mortality rates. This was desirable because HRs are often less variable across similar populations and time and can be more precisely estimated in combined data synthesis as in our study. Generalisability of the findings was enhanced by inclusion of data from 97 prospective studies based in many different Western populations recruited between 1964 and 2009 and latest follow up between 1980 and 2020.

Our study also had potential limitations. Contributing prospective studies defined diabetes in varying ways. There were, however, no major differences in results across studies due to such variation. Between-study heterogeneity of associations was moderate to high, and not explained by the characteristics assessed in subgroup analyses. We did not have information as to the pathophysiological subtype of diabetes. However, given that we excluded participants diagnosed with diabetes at age <30 years, it may be reasonable to infer that the large majority of participants had type 2 diabetes.^50^ We did not have information on whether individuals with diabetes were differentially treated and/or followed up depending on age at diagnosis or duration of diabetes (e.g. specific type of medication, dose or intensity of treatment), factors which are likely to have had an impact on long-term disease outcomes. Residual confounding due to measurement error in variables considered for adjustment (e.g. smoking) has not been addressed. We also did not have information on other co-morbidities (e.g. mental health) and socio-economic variables that would have been useful to adjust for. The present analysis involved participants who were mostly of European continental ancestry; future studies should seek to evaluate these results in other ethnic and racial groups. Finally, while we found broadly similar results for cause-specific mortality using competing and non-competing adjusted models, the aetiological interpretation is limited for models adjusted for competing risk.^51^ However, non-competing risk adjusted models may be subject to selection bias as HRs are calculated conditional on those who have survived.^52^

In conclusion, our study has suggested that every decade of earlier diagnosis of diabetes is associated with about three to four years of lower life expectancy, highlighting the potential value of early interventions that delay or prevent diabetes.

## Supplementary Material

Supplementary material

## Figures and Tables

**Table 1:** Baseline participant characteristics by age at diagnosis of diabetes.

Overall			Age at diagnosis of diabetes*					
ERFC (1017695 participants, 96 cohorts)	N	Mean (SD) or n (%)	No Diabetes	30 to <40 yrs	40 to <50 yrs	50 to <60 yrs	60 to <70yrs	≥70 yrs
No. of participants (Row %)	1017695	1017695 (100.0%)	948775 (93.2%)	3253 (0.3%)	8763 (0.9%)	18605 (1.8%)	21527 (2.1%)	16772 (1.6%)
Prevalent diabetes, No. (Col %)	1017695	23335 (2%)	-	2835 (87%)	5602 (64%)	7047 (38%)	5632 (26%)	2219 (13%)
Incident diabetes, No. (Col %)	1017695	45585 (4%)	-	418 (13%)	3161 (36%)	11558 (62%)	15895 (74%)	14553 (87%)
Age at baseline, mean (SD), yrs	1017695	54.6 (9.7)	54.4 (9.8)	49.4 (8.0)	51.0 (7.4)	55.5 (6.8)	60.6 (6.0)	67.6 (4.9)
Male sex, No. (%)	1017695	463920 (46%)	436576 (46%)	1187 (36%)	4150 (47%)	8246 (44%)	7796 (36%)	5965 (36%)
Female sex, No. (%)	1017695	553775 (54%)	512199 (54%)	2066 (64%)	4613 (53%)	10359 (56%)	13731 (64%)	10807 (64%)
Current smoker, No. (%)	945856	267678 (28%)	252507 (29%)	914 (29%)	2583 (31%)	4763 (27%)	4422 (21%)	2489 (15%)
Systolic blood pressure, mean (SD), mmHg	753256	135 (19)	134 (19)	135 (19)	138 (19)	142 (19)	143 (19)	146 (20)
Body mass index, mean (SD), kg/m^2^	890293	26.0 (4.3)	25.8 (4.1)	27.9 (6.0)	28.6 (5.7)	28.9 (5.5)	28.5 (5.2)	27.8 (4.7)
Total cholesterol, mean (SD), mmol/l	594679	5.80 (1.11)	5.79 (1.11)	5.55 (1.15)	5.69 (1.15)	5.86 (1.12)	5.87 (1.14)	5.93 (1.15)
Random glucose, mean (SD), mmol/l	398521	5.39 (1.23)	5.25 (0.87)	8.20 (4.04)	7.98 (3.58)	7.51 (2.96)	6.94 (2.57)	6.42 (2.31)
Fasting glucose, mean (SD), mmol/l	196159	5.30 (1.09)	5.15 (0.64)	7.88 (3.77)	7.27 (3.38)	6.94 (2.66)	6.48 (2.03)	5.93 (1.33)
HbA1c, mean (SD), %	96802	5.42 (0.79)	5.25 (0.48)	7.37 (1.89)	7.14 (1.79)	6.71 (1.59)	6.30 (1.22)	6.07 (0.95)
History of CVD, No. (%)	1017695	73091 (7%)	63734 (7%)	422 (13%)	1097 (13%)	2256 (12%)	2820 (13%)	2762 (16%)
Medication usage, No. (%)^$^	614036	84056 (14%)	66186 (12%)	1060 (53%)	2610 (47%)	4777 (41%)	5261 (38%)	4162 (33%)
Vocational/University education level, No. (%)	389924	99850 (26%)	94710 (26%)	313 (23%)	775 (23%)	1578 (23%)	1583 (21%)	891 (16%)
Non-white ethnic group, No. (%)	534131	71348 (13%)	61154 (12%)	617 (35%)	1781 (33%)	3378 (30%)	2817 (22%)	1601 (15%)
**UK Biobank (498023 participants)**	**N**	**Mean (SD) or n (%)**	**No Diabetes**	**30 to <40 yrs**	**40 to <50 yrs**	**50 to <60 yrs**	**60 to <70yrs**	**≥70 yrs**
No. of participants (Row %)	498023	498023 (100.0%)	471223 (94.6%)	1550 (0.3%)	5859 (1.2%)	11041 (2.2%)	7406 (1.5%)	944 (0.2%)
Prevalent diabetes, No. (Col %)	498023	24981 (5%)	-	1550 (100%)	5810 (99%)	10798 (98%)	6818 (92%)	5 (1%)
Incident diabetes, No. (Col %)	498023	1819 (0%)	-	0 (0%)	49 (1%)	243 (2%)	588 (8%)	939 (99%)
Age at baseline, mean (SD), yrs	498023	57.0 (8.1)	56.8 (8.1)	52.8 (8.5)	54.0 (7.1)	60.8 (4.9)	65.4 (4.3)	62.0 (5.3)
Male sex, No. (%)	498023	226676 (46%)	210224 (45%)	882 (57%)	3560 (61%)	6889 (62%)	4508 (61%)	613 (65%)
Female sex, No. (%)	498023	271347 (54%)	260999 (55%)	668 (43%)	2299 (39%)	4152 (38%)	2898 (39%)	331 (35%)
Current smoker, No. (%)	497789	52494 (11%)	49497 (11%)	243 (16%)	815 (14%)	1193 (11%)	665 (9%)	81 (9%)
Systolic blood pressure, mean (SD), mmHg	497112	138 (19)	137 (19)	137 (17)	138 (17)	141 (17)	144 (17)	145 (18)
Body mass index, mean (SD), kg/m^2^	495426	27.5 (4.8)	27.2 (4.6)	31.3 (7.0)	32.1 (6.5)	31.7 (5.7)	30.8 (5.2)	30.3 (4.8)
Total cholesterol, mean (SD), mmol/l	465833	5.70 (1.14)	5.76 (1.11)	4.53 (1.10)	4.53 (1.09)	4.52 (1.05)	4.63 (1.09)	5.60 (1.16)
Random glucose, mean (SD), mmol/l	426110	5.10 (1.19)	4.97 (0.78)	8.93 (4.54)	7.92 (3.85)	7.35 (3.07)	6.67 (2.53)	5.66 (1.75)
Fasting glucose, mean (SD), mmol/l	17712	5.15 (1.12)	5.04 (0.70)	7.42 (4.23)	7.38 (3.59)	7.00 (2.99)	6.23 (2.28)	5.29 (0.81)
HbA1c, mean (SD), %	462791	4.88 (0.89)	4.76 (0.61)	8.20 (2.27)	7.37 (2.20)	6.98 (1.76)	6.53 (1.53)	5.66 (1.20)
History of CVD, No. (%)	498023	69693 (14%)	62346 (13%)	363 (23%)	1316 (22%)	3122 (28%)	2344 (32%)	202 (21%)
Medication usage, No. (%)$	490758	135849 (28%)	113681 (24%)	1364 (90%)	4707 (82%)	9453 (87%)	6173 (85%)	471 (51%)
Vocational/University education level, No. (%)	494242	298113 (60%)	284262 (61 %)	875 (58%)	3237 (57%)	5811 (54%)	3362 (46%)	566 (60%)
Non-white ethnic group, No. (%)	496231	26390 (5%)	23052 (5%)	363 (24%)	1167 (20%)	1234 (11%)	522 (7%)	52 (6%)

* Includes people with a history of diabetes at the baseline survey and people with incident diabetes.

$ Includes use of lipid-lowering, anti-hypertensive, or anti-diabetic medication at baseline.

Differences in characteristics across categories of age at diagnosis of diabetes were all statistically significant (p < 0.001 adjusted for age and sex) based on Wald tests.

**Table 1:** Hazard ratios for all-cause and cause-specific mortality according to age at diagnosis of diabetes with adjustment for conventional risk factors.*

	HR (95% CI) adjusted for...			
	No. of events	Age and sex	Age, sex, and smoking	Age, sex, smoking, and other risk factors**
**All-cause mortality**				
No Diabetes	153068	1 (Ref)	1 (Ref)	1 (Ref)
30 to <40 yrs	676	2.69 (2.43, 2.97)	2.74 (2.49, 3.02)	2.64 (2.41,2.90)
40 to <50 yrs	2070	2.26 (2.08, 2.45)	2.33 (2.14, 2.53)	2.24 (2.06, 2.43)
50 to <60 yrs	4197	1.84 (1.72, 1.97)	1.87 (1.75, 1.99)	1.79 (1.69, 1.90)
60 to <70 yrs	4125	1.57 (1.47, 1.67)	1.60 (1.51,1.70)	1.55 (1.46, 1.64)
≥70 yrs	3026	1.39 (1.29, 1.51)	1.43 (1.33, 1.55)	1.41 (1.31,1.53)
**Per decade earlier**	**167162**	**1.14 (1.08, 1.19)**	**1.13 (1.08, 1.19)**	**1.13 (1.07, 1.19)**
P-value***		<0.0001	<0.0001	<0.0001
I^2^ (95% CI)		67 (59, 74)	68 (60, 74)	67 (60, 74)
**CVD mortality**				
No Diabetes	53857	1 (Ref)	1 (Ref)	1 (Ref)
30 to <40 yrs	278	4.20 (3.57, 4.94)	4.26 (3.65, 4.99)	3.93 (3.41,4.53)
40 to <50 yrs	821	3.19 (2.80, 3.64)	3.31 (2.90, 3.76)	2.93 (2.60, 3.30)
50 to <60 yrs	1564	2.31 (2.10, 2.53)	2.36 (2.16, 2.58)	2.10 (1.94, 2.27)
60 to <70 yrs	1580	1.95 (1.78, 2.14)	1.98 (1.82, 2.17)	1.81 (1.67, 1.97)
≥70 yrs	1251	1.50 (1.35, 1.67)	1.54 (1.38, 1.71)	1.48 (1.33, 1.65)
**Per decade earlier**	**59351**	**1.19 (1.11, 1.27)**	**1.19 (1.11, 1.27)**	**1.19 (1.11, 1.28)**
P-value***		<0.0001	<0.0001	<0.0001
I^2^ (95% CI)		58 (47, 67)	59 (48, 67)	59 (48, 67)
**Cancer mortality**				
No Diabetes	53217	1 (Ref)	1 (Ref)	1 (Ref)
30 to <40 yrs	124	1.55 (1.30, 1.85)	1.56 (1.31,1.86)	1.48 (1.24, 1.76)
40 to <50 yrs	433	1.28 (1.16, 1.42)	1.32 (1.19, 1.45)	1.25 (1.13, 1.37)
50 to <60 yrs	1211	1.33 (1.22, 1.46)	1.37 (1.25, 1.49)	1.35 (1.26, 1.44)
60 to <70 yrs	1178	1.27 (1.17, 1.38)	1.29 (1.19, 1.40)	1.28 (1.19, 1.37)
≥70 yrs	593	1.29 (1.18, 1.42)	1.32 (1.20, 1.44)	1.31 (1.20, 1.42)
**Per decade earlier**	**56756**	**0.95 (0.88, 1.02)**	**0.95 (0.88, 1.02)**	**0.94 (0.88, 1.02)**
P-value***		0.176	0.160	0.128
I^2^ (95% CI)		26 (2, 44)	24 (0, 43)	24 (0, 43)
**Non-CVD, non-cancer mortality**				
No Diabetes	35986	1 (Ref)	1 (Ref)	1 (Ref)
30 to <40 yrs	250	3.99 (3.50, 4.55)	4.04 (3.54, 4.60)	3.90 (3.42, 4.45)
40 to <50 yrs	701	3.24 (2.88, 3.64)	3.34 (2.96, 3.76)	3.31 (2.95, 3.71)
50 to <60 yrs	1224	2.31 (2.09, 2.54)	2.37 (2.15, 2.60)	2.38 (2.16, 2.64)
60 to <70 yrs	1137	1.84 (1.67, 2.02)	1.87 (1.70, 2.05)	1.88 (1.71,2.06)
≥70 yrs	861	1.66 (1.48, 1.87)	1.71 (1.52, 1.93)	1.76 (1.56, 1.98)
**Per decade earlier**	**40159**	**1.18 (1.10, 1.27)**	**1.18 (1.09, 1.27)**	**1.16 (1.08, 1.25)**
P-value***		<0.0001	<0.0001	<0.0001
I^2^ (95% CI)		50 (35, 61)	51 (36, 62)	50 (36, 62)

* Analyses based on ERFC and UK Biobank, including 92 cohorts and 1,132,277 participants with complete information on age at diagnosis of diabetes, age, sex, smoking and other risk factors.

** Other risk factors were body mass index, systolic blood pressure and total cholesterol.

*** P-value for log-linear analyses per decade earlier.

## Data Availability

Data from UK Biobank is available to any *bona fide* scientific research on application. Data from the Emerging Risk Factors Collaboration is available at the discretion of the principal investigators of the individual studies.

## Research in Context

### Evidence before this study

We searched articles published in Medline from inception to November 30, 2022 that reported on associations of age at diagnosis or duration of diabetes with all-cause mortality, and on years of life lost according to age at diagnosis of diabetes. Few population cohorts have had sufficient statistical power, detail, and duration of follow-up to enable reliable estimation, or have directly analysed the associations of age at diagnosis of diabetes *per se* with mortality and life expectancy.

### Added value of this study

Using data from large-scale data sources in 19 high-income countries involving individual records on 1,515,718 participants in whom deaths were recorded during 23.1 million person-years of follow-up, we calculated age- and sex-adjusted hazard ratios for all-cause mortality according to age at diagnosis of diabetes and estimated cumulative survival by applying the age-specific estimates to contemporary age-specific death rates. We found a steep log-linear dose-response association between earlier age at diagnosis of diabetes and higher risk of all-cause mortality. Every decade of earlier diagnosis of diabetes was associated with about four years of reduced life expectancy. Our public health modelling has suggested that, for individuals surviving to age 50 years, those with diabetes diagnosed at age 30, 40, and 50 years died, on average, 14, 10, and 6 years earlier, respectively, than individuals without diabetes.

### Implications of all the available evidence

As earlier diagnosis of diabetes is associated with shorter life expectancy, high priority should be given to developing and implementing interventions that prevent or delay onset of diabetes, especially as the prevalence of diabetes among younger adults is rising globally. In addition, the evidence highlights the need for intensive treatment of risk factors for premature mortality among young adults diagnosed with diabetes.
