# Supplementary material for: Life expectancy associated with different ages at diagnosis of diabetes: 23 million person-years of observation
Source: Lancet Diabetes Endocrinol. Author manuscript; Available in PMC 2023 Nov 13. (PMC7615299; doi:10.1016/S2213-8587(23)00223-1)

Life expectancy associated with different ages at diagnosis of diabetes: 23 million person-years of observation

The Emerging Risk Factors Collaboration

Contents list of supplementary material

Supplementary Tables

		Page
**eTable 1**	Cohort-specific characteristics: Exposure details	2
**eTable 2**	Cohort-specific characteristics: Ascertainment of diabetes and deaths	4
**eTable 3**	Cohort-specific characteristics: Follow up and outcomes	5
**eTable 4**	Outcome definitions used in current analyses	6
**eTable 5**	Hazard ratios for all-cause and cause-specific mortality according to age of diagnosis, with adjustment for markers of glycemia, renal function, inflammation and lipids	7
**eTable 6**	Assessment of additive interactions by category of age at diagnosis of diabetes	8
**eTable 7**	Hazard ratios for all-cause and cause-specific mortality according to duration of diabetes with adjustment for conventional risk factors	9
**eTable 8**	Hazard ratios for all-cause and cause-specific mortality in ERFC per decade higher duration of diabetes without and with adjustment for markers of glycemia, renal function, inflammation and lipids	10
**eTable 9**	Hazard ratios for components of non-CVD mortality per decade earlier age at diagnosis of diabetes adjusted for conventional risk factors	11
**eTable 10**	Hazard ratios and subdistribution hazard ratios for all-cause and cause-specific mortality per decade earlier age at diagnosis of diabetes with/without adjustment for competing risks	12

Supplementary Figures

**eFigure 1**	Associations of age at diagnosis of diabetes with all-cause and cause-specific mortality adjusted for age and sex.	13
**eFigure 2**	Hazard ratios for all-cause mortality in ERFC per decade earlier age at diagnosis of diabetes according to selected participant and study-level characteristics.	14
**eFigure 3**	Cohort-specific hazard ratios for all-cause mortality in ERFC per decade earlier age at diagnosis of diabetes according to calendar time of study enrolment and follow-up period.	15
**eFigure 4**	Hazard ratios for all-cause and cause-specific mortality in ERFC and UK Biobank separately according to age at diagnosis of diabetes adjusted for age and sex	16
**eFigure 5**	Hazard ratios for all-cause and cause-specific mortality according to duration of diabetes adjusted for age and sex (top) and sex-specific (bottom).	17
**eFigure 6**	Cohort specific percentages of right censoring, deaths and loss to follow up.	18
**eFigure 7**	Estimated cause-specific contributions to overall reduction in life expectancy by age at diagnosis of diabetes using US 2015 death rates.	20
**eFigure 8**	Estimated cause-specific contributions to overall reduction in life expectancy by age at diagnosis of diabetes using EU 2015 death rates.	21
**eFigure 9**	Estimated cause-specific contributions to overall reduction in life expectancy by age at diagnosis of diabetes using US 2015 death rates, including diabetes diagnosed before age 30 years.	22
**eFigure 10**	Estimated cause-specific contributions to overall reduction in life expectancy by age at diagnosis of diabetes using EU 2015 death rates, including diabetes diagnosed before age 30 years.	23

Supplementary Appendices

**eAppendix 1**	List of ERFC study acronyms	24
**eAppendix 2**	UK Biobank	26
**eAppendix 3**	Calculation of age at diagnosis of diabetes mellitus	27
**eAppendix 4**	Statistical methods used for estimating years of life lost	28

eTable 1. Cohort-specific characteristics: Definition of analysis sample, primary exposure, and summary of baseline characteristics.

Cohort abbreviation	Country	Median year of baseline	Maximum year of follow up	Total participants	Diabetes at baseline, N (%)	Diabetes duration information, N (%)	Included in analyses, Yes (%)	Age at survey (yrs) mean (sd)	Sex, Male (%)	Prevalent Diabetes duration (yrs) mean (sd)	Age at diagnosis of prevalent diabetes (yrs) mean (sd)	Incident diabetes, N (%)	Incident diabetes time inferred, N (%)	Incident diabetes time provided, N (%)	Time to incident diabetes (yrs) mean (sd)	Age at incident diabetes (yrs) mean (sd)	Accuracy of incident diabetes time (yrs) mean (sd)	History of CVD at baseline, Yes (%)
ARIC	USA	1988	2011	15638	1889 (12.1)	831 (44)	14580 (93)	55 (6)	6515 (45)	8.4 (6.8)	48 (8)	1534 (9.8)	22 (1)	1512 (99)	5.2 (2.4)	60 (6)	1.43 (0.11)	2249 (15.4)
AUSDIAB	Australia	2000	2012	11236	564 (5.0)	29 (5)	10701 (95)	51 (14)	4758 (44)	8.1 (6.8)	61 (12)	632 (5.6)	544 (86)	88 (14)	7.7 (3.2)	62 (12)	2.87 (0.44)	879 (8.2)
BHS	Australia	1972	2004	7152	144 (2.0)	101 (70)	7109 (99)	46 (17)	3387 (48)	7.4 (7.5)	54 (11)	234 (3.3)	234 (100)	0 (0)	15.2 (7.8)	66 (11)	4.94 (3.13)	831 (11.7)
BRUN	Italy	1990	2010	910	45 (4.9)	45 (100)	910 (100)	59 (12)	466 (51)	8.7 (5.1)	61 (11)	105 (11.5)	89 (85)	16 (15)	10.8 (5.4)	71 (10)	0.45 (0.86)	125 (13.7)
BWHHS	UK	2000	2014	4277	228 (5.3)	219 (96)	4268 (100)	69 (6)	0 (0)	8.0 (6.7)	61 (8)	218 (5.1)	14 (6)	204 (94)	5.0 (2.1)	73 (6)	1.82 (0.28)	1281 (30.0)
CHARL	USA	1961	2009	2265	96 (4.2)	25 (26)	2194 (97)	50 (11)	1047 (48)	4.8 (4.9)	42 (9)	351 (15.5)	150 (43)	201 (57)	19.6 (7.6)	65 (9)	4.05 (1.56)	204 (9.3)
CHS	USA	1990	2007	5832	978 (16.8)	191 (20)	5045 (87)	73 (5)	2078 (41)	10.8 (8.7)	60 (9)	439 (7.5)	386 (88)	53 (12)	4.5 (2.1)	77 (6)	0.50 (0.06)	1263 (25.0)
COPEN	Denmark	1993	2013	10049	345 (3.4)	184 (53)	9888 (98)	58 (15)	4306 (44)	8.6 (5.5)	57 (11)	740 (7.4)	186 (25)	554 (75)	10.9 (4.9)	72 (11)	4.70 (0.20)	1354 (13.7)
DESIR	France	1995	2010	3951	166 (4.2)	124 (75)	3909 (99)	48 (10)	1927 (49)	8.2 (7.6)	48 (8)	187 (4.7)	132 (71)	55 (29)	5.9 (2.6)	56 (10)	1.49 (0.10)	60 (1.5)
DRECE	Spain	1991	2011	3092	159 (5.1)	11 (7)	2944 (95)	38 (12)	1437 (49)	5.2 (6.3)	49 (6)	24 (0.8)	0 (0)	24 (100)	14.8 (0.4)	63 (10)	-	42 (1.4)
EPESEIOW	USA	1982	2008	1912	140 (7.3)	137 (98)	1909 (100)	73 (5)	667 (35)	10.3 (8.5)	62 (9)	160 (8.4)	25 (16)	135 (84)	4.5 (1.9)	77 (6)	0.51 (0.03)	327 (17.1)
EPESENHA	USA	1982	1992	985	110 (11.2)	108 (98)	983 (100)	73 (6)	383 (39)	10.1 (9.0)	63 (10)	79 (8.0)	9 (11)	70 (89)	3.8 (2.2)	76 (6)	0.59 (0.09)	173 (17.6)
EPICNOR	UK	1996	2005	21712	620 (2.9)	532 (86)	21624 (100)	59 (9)	9852 (46)	6.5 (8.1)	58 (11)	713 (3.3)	0 (0)	713 (100)	6.0 (2.4)	68 (8)	-	930 (4.3)
ESTHER	Germany	2001	2015	9651	1405 (14.6)	932 (66)	9178 (95)	62 (7)	4099 (45)	8.1 (7.3)	56 (9)	1339 (13.9)	0 (0)	1339 (100)	6.9 (3.8)	68 (7)	-	1788 (19.5)
FINE_FIN	Finland	1984	1999	452	40 (8.8)	37 (93)	449 (99)	72 (5)	449 (100)	6.9 (6.1)	67 (6)	20 (4.4)	16 (80)	4 (20)	8.0 (3.2)	78 (3)	2.50 (0.00)	0 (0.0)
FINRISK92	Finland	1992	2008	6004	104 (1.7)	104 (100)	6004 (100)	45 (11)	2834 (47)	6.7 (6.5)	49 (8)	450 (7.5)	0 (0)	450 (100)	11.0 (4.3)	61 (9)	-	229 (3.8)
FINRISK97	Finland	1997	2008	8355	277 (3.3)	223 (81)	8301 (99)	49 (14)	4179 (50)	7.3 (6.8)	55 (10)	501 (6.0)	0 (0)	501 (100)	7.1 (3.4)	63 (11)	-	425 (5.1)
FRAMOFF	USA	1992	2005	3378	221 (6.5)	191 (86)	3348 (99)	54 (10)	1514 (45)	7.0 (5.7)	52 (10)	283 (8.4)	167 (59)	116 (41)	7.4 (2.7)	64 (9)	1.85 (0.65)	0 (0.0)
FUNAGATA	Japan	1991	2003	2766	1 (0.0)	1 (100)	2766 (100)	57 (12)	1219 (44)	1 (-)	42 (-)	61 (2.2)	0 (0)	61 (100)	3.8 (1.3)	67 (9)	-	0 (0.0)
GOTO13	Sweden	1963	1998	794	18 (2.3)	13 (72)	789 (99)	50 (1)	789 (100)	6.3 (6.1)	47 (9)	13 (1.6)	0 (0)	13 (100)	9.1 (2.3)	59 (2)	-	12 (1.5)
GOTO33	Sweden	1984	1998	749	23 (3.1)	14 (61)	740 (99)	51 (0)	740 (100)	5.4 (4.1)	45 (4)	39 (5.2)	0 (0)	39 (100)	6.1 (4.0)	57 (4)	-	20 (2.7)
GOTO43	Sweden	1993	2003	790	19 (2.4)	9 (47)	780 (99)	50 (0)	780 (100)	5.3 (4.4)	45 (4)	14 (1.8)	0 (0)	14 (100)	5.7 (2.7)	56 (3)	-	20 (2.6)
GOTOW	Sweden	1969	2009	2222	18 (0.8)	11 (61)	2215 (100)	46 (8)	0 (0)	3.5 (3.5)	52 (7)	146 (6.6)	10 (7)	136 (93)	21.9 (9.4)	69 (9)	4.67 (1.04)	75 (3.4)
HBS	Finland	1986	2013	1215	47 (3.9)	35 (74)	1203 (99)	60 (4)	1203 (100)	5.0 (3.7)	59 (7)	111 (9.1)	0 (0)	111 (100)	15.7 (4.2)	74 (5)	-	57 (4.7)
HCS	UK	2001	2012	3209	448 (14.0)	195 (44)	2956 (92)	66 (3)	1549 (52)	7.3 (7.1)	59 (8)	16 (0.5)	12 (75)	4 (25)	6.4 (3.5)	71 (4)	1.13 (0.22)	459 (15.5)
HIMS	Australia	1997	2010	12192	1406 (11.5)	476 (34)	11262 (92)	72 (4)	11262 (100)	7.6 (7.7)	64 (8)	251 (2.1)	207 (82)	44 (18)	5.8 (0.9)	77 (3)	2.90 (0.43)	3148 (28.0)
HOORN	Netherlands	1991	2005	2463	257 (10.4)	103 (40)	2309 (94)	61 (7)	1063 (46)	7.4 (6.4)	58 (10)	135 (5.5)	47 (35)	88 (65)	6.3 (0.7)	69 (7)	3.21 (0.24)	213 (9.2)
HPFS1	USA	1986	2010	51321	1519 (3.0)	1491 (98)	51293 (100)	55 (10)	51293 (100)	9.4 (7.4)	52 (10)	3445 (6.7)	0 (0)	3445 (100)	11.0 (5.3)	66 (9)	-	2606 (5.1)
LASA	Netherlands	1993	2010	2659	254 (9.6)	236 (93)	2641 (99)	70 (9)	1293 (49)	11.2 (9.9)	62 (12)	136 (5.1)	53 (39)	83 (61)	7.1 (3.5)	75 (8)	1.18 (0.70)	751 (28.4)
MDC	Sweden	1994	2010	28042	1121 (4.0)	467 (42)	27388 (98)	58 (8)	10698 (39)	9.1 (7.2)	51 (9)	830 (3.0)	0 (0)	830 (100)	8.3 (4.2)	66 (8)	-	1028 (3.8)
MESA	USA	2001	2012	6785	851 (12.5)	536 (63)	6470 (95)	62 (10)	3026 (47)	9.3 (7.9)	55 (10)	483 (7.1)	189 (39)	294 (61)	3.2 (1.6)	65 (10)	0.85 (0.15)	9 (0.1)
MONICA_KORA3	Germany	1995	2009	4846	231 (4.8)	206 (89)	4821 (99)	50 (14)	2384 (49)	8.4 (8.2)	54 (9)	175 (3.6)	0 (0)	175 (100)	7.8 (4.1)	66 (11)	-	170 (3.5)
MOSWEGOT	Sweden	1990	2003	4525	101 (2.2)	37 (37)	4461 (99)	46 (11)	2139 (48)	9.3 (7.1)	48 (9)	52 (1.1)	0 (0)	52 (100)	7.2 (3.5)	63 (7)	-	116 (2.6)
MRCOLD	UK	1996	2007	14035	1101 (7.8)	863 (78)	13797 (98)	80 (4)	5434 (39)	9.2 (8.6)	71 (9)	324 (2.3)	0 (0)	324 (100)	4.2 (0.7)	85 (4)	-	3113 (22.6)
NHANESI	USA	1973	1993	16036	756 (4.7)	754 (100)	16034 (100)	47 (17)	6279 (39)	8.9 (9.4)	53 (12)	833 (5.2)	62 (7)	771 (93)	10.7 (3.1)	64 (13)	2.99 (1.89)	2545 (15.9)
NHS1	USA	1976	2009	121214	1816 (1.5)	1523 (84)	120921 (100)	42 (7)	0 (0)	5.3 (4.5)	42 (6)	9599 (7.9)	0 (0)	9599 (100)	19.3 (7.2)	62 (9)	-	508 (0.4)
PARIS1	France	1970	1993	7061	252 (3.6)	242 (96)	7051 (100)	47 (2)	7051 (100)	4.5 (3.5)	43 (4)	39 (0.6)	39 (100)	0 (0)	1.4 (0.3)	48 (2)	0.68 (0.18)	0 (0.0)
PREVEND	Netherlands	1998	2010	8591	339 (3.9)	68 (20)	8320 (97)	49 (13)	4132 (50)	6.3 (4.0)	56 (10)	79 (0.9)	79 (100)	0 (0)	3.5 (1.1)	60 (10)	3.00 (0.00)	668 (8.0)
QUEBEC	Canada	1974	2002	3614	181 (5.0)	54 (30)	3487 (96)	46 (8)	3487 (100)	3.3 (2.3)	55 (8)	143 (4.0)	66 (46)	77 (54)	9.7 (4.3)	58 (9)	1.58 (0.38)	106 (3.0)
RANCHO	USA	1985	2006	2448	134 (5.5)	65 (49)	2379 (97)	70 (11)	1037 (44)	12.6 (9.6)	56 (12)	151 (6.2)	97 (64)	54 (36)	8.8 (2.5)	75 (9)	2.06 (0.24)	577 (24.3)
SHIP	Germany	1999	2011	4280	458 (10.7)	308 (67)	4130 (96)	49 (16)	2003 (48)	10.5 (7.0)	55 (10)	173 (4.0)	173 (100)	0 (0)	7.9 (2.8)	65 (12)	2.77 (0.36)	333 (8.1)
SHS	USA	1990	2003	2733	1064 (38.9)	680 (64)	2349 (86)	57 (8)	1019 (43)	10.7 (7.2)	48 (9)	348 (12.7)	139 (40)	209 (60)	5.3 (2.0)	61 (8)	1.94 (0.30)	114 (4.9)
TOYAMA	Japan	1996	2008	4534	203 (4.5)	189 (93)	4520 (100)	46 (7)	2906 (64)	6.2 (4.0)	44 (7)	385 (8.5)	0 (0)	385 (100)	6.7 (3.5)	51 (6)	-	0 (0.0)
TROMSØ	Norway	1987	2009	26581	318 (1.2)	287 (90)	26550 (100)	42 (16)	12590 (47)	8.5 (7.9)	60 (13)	468 (1.8)	8 (2)	460 (98)	11.1 (4.9)	61 (13)	4.05 (0.09)	823 (3.1)
ULSAM	Sweden	1972	2008	2318	126 (5.4)	7 (6)	2199 (95)	50 (1)	2199 (100)	1.1 (0.8)	50 (5)	315 (13.6)	118 (37)	197 (63)	23.0 (7.3)	73 (7)	4.71 (0.96)	33 (1.5)
USPHS2	USA	1997	2008	10723	24 (0.2)	23 (96)	10722 (100)	64 (8)	10722 (100)	6.1 (9.0)	57 (10)	707 (6.6)	0 (0)	707 (100)	5.3 (2.9)	70 (8)	-	0 (0.0)
WHIOS	USA	1994	2012	92865	5030 (5.4)	4927 (98)	92762 (100)	64 (7)	0 (0)	9.7 (8.3)	55 (10)	7167 (7.7)	0 (0)	7167 (100)	7.4 (3.7)	70 (8)	-	10084 (10.9)
WHS	USA	1994	2013	27969	715 (2.6)	678 (95)	27932 (100)	55 (7)	0 (0)	6.9 (6.2)	50 (9)	2379 (8.5)	77 (3)	2302 (97)	10.0 (4.0)	64 (7)	2.71 (0.15)	0 (0.0)
ZUTE	Netherlands	1985	2000	921	90 (9.8)	63 (70)	894 (97)	63 (13)	894 (100)	7.6 (6.9)	62 (10)	77 (8.4)	27 (35)	50 (65)	16.3 (12.5)	77 (5)	2.50 (0.02)	99 (11.1)
SUBTOTAL		1991	2015	587352	26452 (4.5)	18585 (70)	579485 (99)	56 (9)	199089 (34)	7.6 (7.5)	54 (9)	37103 (6.3)	3377 (9)	33726 (91)	8.8 (5.1)	67 (9)	2.32 (1.00)	39847 (6.9)
ATENA	Italy	1995	2001	4959	123 (2.5)	106 (86)	4942 (100)	50 (7)	0 (0)	8.8 (6.7)	48 (8)	-	-	-	-	-	-	216 (4.4)
BRHS1	UK	1999	2005	4247	290 (6.8)	284 (98)	4241 (100)	69 (6)	4241 (100)	8.4 (7.4)	61 (8)	-	-	-	-	-	-	1068 (25.2)
FINNMARK	Norway	2002	2009	6348	328 (5.2)	291 (89)	6311 (99)	59 (11)	3001 (48)	8.2 (6.7)	57 (9)	-	-	-	-	-	-	884 (14.0)
HISAYAMA	Japan	1988	2002	2574	206 (8.0)	193 (94)	2561 (99)	59 (11)	1078 (42)	8.9 (7.9)	53 (10)	-	-	-	-	-	-	0 (0.0)

HUBRO	Norway	2001	2009	20747	505 (2.4)	429 (85)	20671 (100)	48 (15)	9243 (45)	8.3 (7.4)	55 (13)	-	-	-	-	-	-	1190 (5.8)
KARELIA	Finland	1972	2008	11081	128 (1.2)	63 (49)	11016 (99)	41 (10)	5380 (49)	3.4 (2.6)	46 (7)	-	-	-	-	-	-	226 (2.1)
MCVDRFP	Netherlands	1989	2007	27569	241 (0.9)	241 (100)	27569 (100)	41 (11)	12941 (47)	5.9 (5.7)	46 (8)	-	-	-	-	-	-	1724 (6.3)
MIDFAM	UK	1996	2013	2330	21 (0.9)	19 (90)	2328 (100)	45 (6)	1035 (44)	4.4 (4.4)	44 (7)	-	-	-	-	-	-	148 (6.4)
MORGEN	Netherlands	1995	2007	22976	251 (1.1)	251 (100)	22976 (100)	42 (11)	10400 (45)	5.8 (5.9)	47 (8)	-	-	-	-	-	-	455 (2.0)
MPP	Sweden	1980	2010	33290	1105 (3.3)	264 (24)	32449 (97)	46 (7)	21851 (67)	5.2 (4.8)	44 (7)	-	-	-	-	-	-	825 (2.5)
NHANESIII	USA	1990	2013	18434	1494 (8.1)	1350 (90)	18290 (99)	49 (20)	8658 (47)	10.5 (8.7)	55 (12)	-	-	-	-	-	-	2002 (10.9)
NSHS	Canada	1995	2005	2776	134 (4.8)	133 (99)	2775 (100)	49 (19)	1380 (50)	9.2 (8.8)	56 (12)	-	-	-	-	-	-	609 (21.9)
OPPHED	Norway	2001	2009	11972	317 (2.6)	296 (93)	11951 (100)	50 (14)	5486 (46)	8.4 (7.7)	57 (12)	-	-	-	-	-	-	849 (7.1)
OSLO2	Norway	2000	2009	6428	413 (6.4)	383 (93)	6398 (100)	69 (6)	6398 (100)	9.7 (7.7)	61 (8)	-	-	-	-	-	-	1355 (21.2)
RS_II	Netherlands	2000	2011	2587	296 (11.4)	169 (57)	2460 (95)	65 (8)	1105 (45)	6.6 (6.3)	60 (10)	-	-	-	-	-	-	416 (16.9)
RS_III	Netherlands	2007	2011	3525	288 (8.2)	200 (69)	3437 (98)	57 (7)	1482 (43)	7.2 (5.6)	53 (9)	-	-	-	-	-	-	202 (5.9)
TROMS	Norway	2002	2009	2429	78 (3.2)	78 (100)	2429 (100)	49 (14)	1065 (44)	7.3 (5.7)	59 (11)	-	-	-	-	-	-	159 (6.5)
SUBTOTAL		1996	2013	184272	6218 (3.4)	4750 (76)	182804 (99)	52 (12)	94744 (52)	7.4 (7.4)	53 (11)	-	-	-	-	-	-	12328 (6.7)
AFTCAPS	USA	1992	1997	6577	127 (1.9)	0 (0)	6450 (98)	58 (7)	5480 (85)	-	-	84 (1.3)	0 (0)	84 (100)	3.3 (1.5)	61 (7)	-	160 (2.5)
ATTICA	Greek	2001	2012	2980	210 (7.0)	0 (0)	2770 (93)	45 (13)	1376 (50)	-	-	99 (3.3)	0 (0)	99 (100)	5.0 (0.0)	58 (11)	-	0 (0.0)
EAS	Scotland	1988	2008	1542	92 (6.0)	0 (0)	1450 (94)	65 (6)	732 (50)	-	-	13 (0.8)	5 (38)	8 (62)	5.5 (0.1)	70 (7)	2.72 (0.04)	488 (33.7)
EPESEBOS	USA	1982	1993	1242	167 (13.4)	0 (0)	1075 (87)	72 (5)	380 (35)	-	-	123 (9.9)	115 (93)	8 (7)	3.8 (2.1)	76 (5)	0.55 (0.11)	169 (15.7)
EPESENCA	USA	1986	2006	1746	257 (14.7)	0 (0)	1489 (85)	72 (5)	529 (36)	-	-	120 (6.9)	64 (53)	56 (47)	4.2 (2.0)	76 (6)	0.59 (0.10)	315 (21.2)
FINE_IT	Italy	1985	2006	681	63 (9.3)	0 (0)	618 (91)	73 (5)	618 (100)	-	-	22 (3.2)	22 (100)	0 (0)	9.4 (3.2)	80 (5)	2.59 (0.65)	185 (29.9)
GOH	Israel	1970	2006	2804	287 (10.2)	0 (0)	2517 (90)	43 (8)	1254 (50)	-	-	167 (6.0)	143 (86)	24 (14)	27.4 (9.4)	68 (10)	10.60 (2.79)	0 (0.0)
IKNS	Japan	1991	2008	8257	583 (7.1)	0 (0)	7674 (93)	58 (10)	3112 (41)	-	-	515 (6.2)	310 (60)	205 (40)	6.8 (4.2)	65 (9)	1.24 (0.91)	184 (2.4)
ISRAEL	Israel	1963	1986	10052	485 (4.8)	0 (0)	9567 (95)	49 (7)	9567 (100)	-	-	395 (3.9)	370 (94)	25 (6)	3.6 (1.4)	55 (7)	1.21 (0.23)	2097 (21.9)
KIHD	Finland	1986	2009	2663	147 (5.5)	0 (0)	2516 (94)	53 (5)	2516 (100)	-	-	206 (7.7)	206 (100)	0 (0)	15.7 (6.2)	68 (8)	3.34 (0.68)	537 (21.3)
MATISS83	Italy	1984	2002	2676	134 (5.0)	0 (0)	2542 (95)	51 (10)	1195 (47)	-	-	87 (3.3)	87 (100)	0 (0)	7.9 (3.7)	62 (8)	3.11 (1.37)	105 (4.1)
MATISS87	Italy	1987	2002	2184	91 (4.2)	0 (0)	2093 (96)	52 (10)	937 (45)	-	-	37 (1.7)	37 (100)	0 (0)	7.6 (1.3)	63 (8)	3.81 (0.67)	83 (4.0)
MIDCOLL	UK	1971	2010	5926	43 (0.7)	0 (0)	5883 (99)	48 (7)	5042 (86)	-	-	17 (0.3)	17 (100)	0 (0)	5.2 (0.9)	54 (7)	2.61 (0.45)	842 (14.3)
MIDRP	UK	1974	2010	14610	194 (1.3)	0 (0)	14416 (99)	55 (6)	6597 (46)	-	-	49 (0.3)	49 (100)	0 (0)	4.2 (0.7)	60 (5)	2.10 (0.36)	3629 (25.2)
MONFRI86	Italy	1986	2002	1168	12 (1.0)	0 (0)	1156 (99)	49 (9)	567 (49)	-	-	1 (0.1)	0 (0)	1 (100)	-	-	-	27 (2.3)
MRFIT	USA	1974	1985	12336	109 (0.9)	0 (0)	12227 (99)	47 (6)	12227 (100)	-	-	1515 (12.3)	435 (29)	1080 (71)	3.7 (1.8)	51 (6)	0.42 (0.12)	31 (0.3)
NCS1	Norway	1976	2012	28073	196 (0.7)	0 (0)	27877 (99)	41 (6)	13738 (49)	-	-	214 (0.8)	214 (100)	0 (0)	8.0 (2.7)	52 (5)	2.58 (0.25)	240 (0.9)
NCS2	Norway	1975	2012	16591	84 (0.5)	0 (0)	16507 (99)	40 (7)	8355 (51)	-	-	128 (0.8)	128 (100)	0 (0)	7.6 (2.6)	50 (6)	2.55 (0.16)	125 (0.8)
NCS3	Norway	1974	2012	15652	40 (0.3)	0 (0)	15612 (100)	38 (8)	7955 (51)	-	-	192 (1.2)	192 (100)	0 (0)	11.3 (3.7)	55 (6)	4.44 (1.35)	140 (0.9)
NPHSII	UK	1991	2005	3018	75 (2.5)	0 (0)	2943 (98)	57 (3)	2943 (100)	-	-	73 (2.4)	0 (0)	73 (100)	6.7 (3.3)	64 (5)	-	51 (1.7)
OSAKA	Japan	1992	2008	12720	635 (5.0)	0 (0)	12085 (95)	52 (10)	8161 (68)	-	-	840 (6.6)	543 (65)	297 (35)	5.9 (4.4)	60 (10)	0.61 (0.54)	207 (1.7)
PRHHP	Caribbean	1967	1980	9815	961 (9.8)	0 (0)	8854 (90)	54 (7)	8854 (100)	-	-	912 (9.3)	888 (97)	24 (3)	4.1 (1.6)	58 (7)	1.45 (0.22)	3127 (35.3)
PROCAM	Germany	1984	2003	26523	452 (1.7)	0 (0)	26071 (98)	40 (11)	18002 (69)	-	-	97 (0.4)	97 (100)	0 (0)	7.3 (4.2)	51 (7)	2.42 (1.48)	209 (0.8)
PROSPER	Scotland/Ireland /Netherland	1998	2002	5804	623 (10.7)	0 (0)	5181 (89)	75 (3)	2456 (47)	-	-	290 (5.0)	0 (0)	290 (100)	2.0 (0.9)	77 (3)	-	2326 (44.9)
REYK	Iceland	1975	2008	18881	420 (2.2)	0 (0)	18461 (98)	53 (9)	8873 (48)	-	-	322 (1.7)	321 (100)	1 (0)	16.2 (7.3)	64 (9)	2.69 (0.74)	1939 (10.5)
RS_I	Netherlands	1992	2005	6441	692 (10.7)	0 (0)	5749 (89)	68 (8)	2357 (41)	-	-	293 (4.5)	293 (100)	0 (0)	6.6 (0.4)	73 (7)	3.26 (0.20)	1359 (23.6)
TARFS	Turkey	1990	2014	4044	99 (2.4)	0 (0)	3945 (98)	41 (13)	1949 (49)	-	-	519 (12.8)	427 (82)	92 (18)	13.4 (5.0)	57 (11)	1.27 (0.49)	0 (0.0)
WCWC	Germany	1989	2008	22014	1042 (4.7)	0 (0)	20972 (95)	42 (11)	20972 (100)	-	-	325 (1.5)	325 (100)	0 (0)	6.1 (3.5)	51 (9)	2.36 (1.33)	1899 (9.1)
WHITEII	UK	1987	2004	10281	94 (0.9)	0 (0)	10187 (99)	45 (6)	6824 (67)	-	-	795 (7.7)	0 (0)	795 (100)	11.3 (4.6)	58 (7)	-	68 (0.7)
WOSCOPS	UK	1990	1995	6595	76 (1.2)	0 (0)	6519 (99)	55 (6)	6519 (100)	-	-	32 (0.5)	0 (0)	32 (100)	4.0 (1.1)	61 (6)	-	374 (5.7)
SUBTOTAL		1986	2014	263896	8490 (3.2)	0 (0)	255406 (97)	53 (8)	170087 (67)	-	-	8482 (3.2)	5288 (62)	3194 (38)	7.7 (3.7)	62 (8)	2.53 (0.79)	20916 (8.2)
TOTAL ERFC		1990	2015	1035520	41160 (4.0)	23335 (57)	1017695 (98)	55 (10)	463920 (46)	7.6 (7.5)	54 (10)	45585 (4.4)	8665 (19)	36920 (81)	8.4 (4.8)	65 (8)	2.41 (0.88)	73091 (7.2)
TOTAL UKBB	UK	2009	2020	498458	25416 (5.1)	24981 (98)	498023 (100)	57 (8)	226676 (46)	6.5 (6.2)	54 (8)	1819 (0.36)	1226 (67)	593 (33)	7.9 (2.7)	70 (9)	2.35 (0.36)	69693 (14)

eTable 2. Cohort-specific characteristics: Ascertainment of diabetes and deaths

	Diabetes ascertainment		Deaths ascertainment	
Cohort	Prevalent diabetes	Incident diabetes	Information source	ICD codes provided to ERFC?
ARIC	3	3	2	1
AUSDIAB	5	5	1	1
BHS	3	3	1	1
BRUN	3	7	2	0
BWHHS	1	7	2	1
CHARL	1	6	2	1
CHS	4	4	2	0
COPEN	1	4	2	1
DESIR	4	4	1	1
DRECE			1	1
EPESEIOW	1	1	1	1
EPESENHA	1	1	1	1
EPICNOR	1	7	1	0
ESTHER	2	7	1	1
FINE_FIN	1		2	1
FINRISK92	3	2	2	1
FINRISK97	3	6	2	1
FRAMOFF	4	4	2	0
FUNAGATA	4	7	1	1
GOTO13	1	3	2	1
GOTO33	1	3	2	1
GOTO43	1	3	2	1
GOTOW	4	4	1	1
HBS	4	2	2	0
HCS	1	7	1	1
HIMS	1	7	1	1
HOORN	4	4	1	1
HPFS1	2	4	2	1
LASA	2	7	1	0
MDC	1	7	2	1
MESA	4	4	2	0
MONICA_KORA3	2	7	2	1
MOSWEGOT	1	1	2	1
MRCOLD	5		1	1
NHANESI	2	7	1	1
NHS1	2	4	1	1
PARIS1	3	3	2	1
PREVEND	3	4	2	1
QUEBEC	1	4	2	0
RANCHO	3	4	1	1
SHIP	2	1	2	1
SHS	4	4	2	1
TOYAMA			1	1
TROMSØ	2	7	1	1
ULSAM	3	7	2	1
USPHS2	1	1	1	0
WHIOS	2	2	2	0
WHS	4	4	2	0
ZUTE	1	4	2	1
ATENA	3		2	1
BRHS1	1	4	1	1
FINNMARK	1	6	1	1
HISAYAMA	4	4	2	1
HUBRO	1	1	1	1
KARELIA			2	1
MCVDRFP	1	1	1	1
MIDFAM	1	7	1	1
MORGEN	1	7	1	1
MPP	1	6	2	1
NHANESIII	1	1	1	1
NSHS			2	1
OPPHED	1	1	1	1
OSLO2	1	1	1	1
RS_II	3	7	2	1
RS_III	3	7	2	1
TROMS	2	7	1	1
AFTCAPS	1	4	2	0
ATTICA			1	0
EAS	4	4	2	1
EPESEBOS	1	1	1	1
EPESENCA	1	1	1	1
FINE_IT	1		2	1
GOH	4	6	2	1
IKNS	4	4	2	1
ISRAEL	3	3	2	0
KIHD	3	7	2	1
MATISS83	3		2	1
MATISS87	3		2	1
MIDCOLL	1	7	1	1
MIDRP	1	7	1	1
MONFRI86	3		2	1
MRFIT	1	4	2	1
NCS1	1		1	1
NCS2	1		1	1
NCS3	1		1	1
NPHSII	1	6	2	1
OSAKA	5	4	2	1
PRHHP	3	4	2	1
PROCAM	3	3	2	0
PROSPER	4	4	2	0
REYK	4	4	2	1
RS_I	3	7	2	1
TARFS	4	4	1	1
WCWC	1	1	1	1
WHITEII	3	4	1	1
WOSCOPS	1	4	2	0
UKBB	2	7	1	1

Prevalent diabetes:
- 0 Not recorded
- 1 Self report only
- 2 Self report and medications
- 3 Self report and biochemical
- 4 Self report + biochemical + medications
- 5 Biochemical only

Incident diabetes:
- 0 Not recorded
- 1 Self report only
- 2 Self report and medications
- 3 Self report and biochemical
- 4 Self report + biochemical + medications
- 5 Biochemical only
- 6 Medical records only
- 7 Self report and medical records

Deaths information source:
- 1 Death certificate only
- 2 Death certificate plus additional checks*

Deaths ICD codes provided to ERFC?:
- 0 Not provided
- 1 Provided

* e.g. supplemented by medical records, findings on autopsy, and other sources.

eTable 3. Cohort-specific characteristics: Follow up and outcomes

Cohort abbreviation	Median year of baseline	Maximum year of follow up	Median follow-up (5th & 95th percentiles)	CVD mortality	Non-CVD mortality	Cancer mortality	Non-CVD, non-cancer mortality	Unknown/ill-defined mortality	All-cause mortality	Person-years of follow up, fatal event
ARIC	1988	2011	22.5 (7.0 to 24.6)	1436	2996	1633	1357	57	4489	287,957
AUSDIAB	2000	2012	12.6 (7.9 to 13.5)	234	480	289	191	386	1100	130,584
BHS	1972	2004	24.2 (4.2 to 33.2)	1317	1274	680	570	5	2596	163,716
BRUN	1990	2010	20.2 (3.8 to 20.5)	135	204	98	106	4	343	15,211
BWHHS	2000	2014	14.2 (3.9 to 15.5)	465	901	445	456	17	1383	54,089
CHARL	1961	2009	27.3 (4.1 to 48.8)	989	887	380	483	66	1942	59,716
CHS	1990	2007	12.9 (2.9 to 15.9)	1049	1705	682	1023	32	2786	59,129
COPEN	1993	2013	19.1 (2.6 to 21.1)	1379	2582	885	1697	847	4808	150,513
DESIR	1995	2010	14.4 (13.6 to 15.0)	21	48	34	14	7	76	55,908
DRECE	1991	2011	19.3 (17.1 to 19.6)	36	145	88	56	0	181	55,294
EPESEIOW	1982	2008	15.4 (7.3 to 26.5)	815	740	194	539	195	1750	30,696
EPESENHA	1982	1992	10.5 (7.2 to 10.8)	151	61	15	46	48	260	9,835
EPICNOR	1996	2005	9.8 (6.3 to 12.0)	354	0	0	0	0	354	207,915
ESTHER	2001	2015	14.3 (5.3 to 15.3)	673	1188	716	472	112	1973	121,922
FINE_FIN	1984	1999	12.3 (5.7 to 15.0)	151	133	65	68	1	285	5,223
FINRISK92	1992	2008	16.9 (10.3 to 16.9)	136	245	105	140	1	382	97,451
FINRISK97	1997	2008	11.8 (10.6 to 11.9)	162	257	99	158	5	424	95,091
FRAMOFF	1992	2005	12.1 (7.8 to 14.4)	38	237	146	91	33	308	39,664
FUNAGATA	1991	2003	11.2 (5.2 to 12.0)	76	162	105	57	7	245	26,821
GOTO13	1963	1998	29.4 (10.7 to 34.5)	296	211	143	68	54	561	21,271
GOTO33	1984	1998	12.8 (6.9 to 13.1)	27	55	27	28	3	85	9,075
GOTO43	1993	2003	10.0 (9.6 to 10.7)	6	21	16	5	1	28	7,811
GOTOW	1969	2009	28.8 (4.6 to 41.0)	385	478	248	230	81	944	57,905
HBS	1986	2013	26.0 (6.0 to 27.5)	135	248	148	100	312	695	26,228
HCS	2001	2012	8.8 (4.7 to 11.6)	92	239	169	70	1	332	25,728
HIMS	1997	2010	12.7 (2.4 to 14.2)	1719	3256	1855	1401	51	5026	121,339
HOORN	1991	2005	13.6 (4.5 to 14.7)	196	217	157	60	128	541	28,228
HPFS1	1986	2010	20.2 (5.7 to 21.9)	5713	8880	4870	3993	367	14960	919,304
LASA	1993	2010	9.9 (1.5 to 10.5)	0	0	0	0	1006	1006	21,822
MDC	1994	2010	16.2 (6.7 to 19.2)	1710	3353	2282	1071	112	5175	423,677
MESA	2001	2012	8.6 (3.4 to 8.9)	114	363	0	0	3	480	51,592
MONICA_KORA3	1995	2009	14.0 (6.1 to 14.7)	275	380	219	161	22	677	63,121
MOSWEGOT	1990	2003	12.9 (8.0 to 18.6)	101	194	132	62	5	300	56,730
MRCOLD	1996	2007	7.9 (0.9 to 11.7)	4178	4739	1767	2972	278	9195	98,710
NHANESI	1973	1993	17.6 (0.0 to 21.0)	2280	2119	1042	1062	57	4456	222,817
NHS1	1976	2009	28.6 (10.3 to 30.3)	5363	16933	10526	6407	1515	23811	3,185,948
PARIS1	1970	1993	22.9 (7.6 to 26.1)	478	1388	914	463	204	2070	149,184
PREVEND	1998	2010	12.7 (8.0 to 13.2)	215	525	363	162	29	769	101,656
QUEBEC	1974	2002	27.8 (10.6 to 28.2)	378	542	373	169	93	1013	85,376
RANCHO	1985	2006	14.2 (2.5 to 18.2)	594	593	249	341	4	1191	29,878
SHIP	1999	2011	11.3 (6.2 to 12.5)	168	312	184	128	26	506	44,596
SHS	1990	2003	12.7 (2.6 to 14.3)	205	490	161	326	9	704	26,592
TOYAMA	1996	2008	12.7 (8.5 to 12.8)	16	45	29	16	35	96	55,118
TROMSØ	1987	2009	21.9 (9.2 to 22.4)	1281	1800	1000	799	410	3491	483,044
ULSAM	1972	2008	32.2 (8.8 to 37.9)	680	795	506	263	16	1491	62,910
USPHS2	1997	2008	10.9 (7.0 to 11.5)	166	748	0	0	0	914	106,091
WHIOS	1994	2012	12.0 (5.0 to 14.8)	3361	7698	4564	3134	1151	12210	1,029,077
WHS	1994	2013	19.1 (12.3 to 20.0)	297	1317	922	395	0	1614	502,735
ZUTE	1985	2000	15.1 (2.0 to 40.0)	293	323	201	117	47	663	16,682
SUBTOTAL	1991	2015	14.8 (4.9 to 29.0)	40339	72507	39726	31527	7843	120689	9,700,980
ATENA	1995	2001	6.8 (5.2 to 8.1)	12	32	22	10	1	45	33,087
BRHS1	1999	2005	5.0 (2.3 to 5.0)	198	0	0	0	345	543	19,970
FINNMARK	2002	2009	7.5 (4.0 to 7.5)	196	351	194	115	0	547	45,353
HISAYAMA	1988	2002	14.0 (4.8 to 14.0)	163	363	169	194	2	528	32,545
HUBRO	2001	2009	8.5 (5.0 to 9.5)	385	950	509	345	0	1335	177,649
KARELIA	1972	2008	36.8 (8.5 to 36.9)	1979	2041	838	1199	13	4033	337,531
MCVDRFP	1989	2007	16.6 (4.4 to 18.9)	593	1421	967	453	131	2145	431,584
MIDFAM	1996	2013	17.4 (14.1 to 17.8)	27	102	63	39	25	154	39,462
MORGEN	1995	2007	10.6 (0.0 to 13.0)	186	457	340	117	34	677	230,313
MPP	1980	2010	29.2 (8.8 to 34.1)	3527	5963	3676	2287	257	9747	851,318
NHANESIII	1990	2013	18.7 (3.0 to 22.6)	2562	3651	1376	2275	95	6308	302,092
NSHS	1995	2005	9.6 (3.9 to 10.0)	108	267	0	0	1	376	25,233
OPPHED	2001	2009	8.5 (6.0 to 9.5)	277	553	302	192	0	830	100,383
OSLO2	2000	2009	9.5 (2.0 to 9.5)	505	1087	629	320	0	1592	53,934
RS_II	2000	2011	10.0 (4.2 to 10.9)	135	239	152	87	24	398	22,972
RS_III	2007	2011	3.6 (1.9 to 4.8)	19	36	30	6	2	57	11,929
TROMS	2002	2009	7.5 (7.5 to 7.5)	38	60	41	15	0	98	18,004
SUBTOTAL	1996	2013	11.7 (3.5 to 33.9)	10910	17573	9308	7654	930	29413	2,733,360
AFTCAPS	1992	1997	5.1 (4.5 to 6.7)	40	113	81	32	0	153	34,300
ATTICA	2001	2012	5.0 (5.0 to 10.0)	22	16	11	5	0	38	18,087
EAS	1988	2008	18.7 (3.2 to 21.2)	308	456	250	205	52	816	22,675
EPESEBOS	1982	1993	11.4 (7.6 to 11.9)	97	103	32	71	19	219	11,767
EPESENCA	1986	2006	13.6 (7.7 to 20.7)	415	474	153	321	59	948	21,457
FINE_IT	1985	2006	11.2 (1.7 to 21.4)	260	219	153	66	55	534	7,051
GOH	1970	2006	35.0 (16.9 to 36.0)	210	265	144	121	380	855	81,009
IKNS	1991	2008	11.1 (5.1 to 18.6)	217	554	279	255	41	812	87,441
ISRAEL	1963	1986	23.3 (7.3 to 23.9)	1287	1864	0	0	0	3151	196,447
KIHD	1986	2009	21.7 (5.1 to 25.2)	363	428	223	200	2	793	49,337
MATISS83	1984	2002	18.7 (6.7 to 19.5)	259	152	91	61	72	483	43,440
MATISS87	1987	2002	15.6 (7.2 to 16.2)	125	86	45	41	44	255	30,777
MIDCOLL	1971	2010	29.3 (5.9 to 40.1)	1991	2209	1253	956	19	4219	159,690
MIDRP	1974	2010	23.7 (4.2 to 36.8)	5736	6029	3264	2765	59	11824	329,319
MONFRI86	1986	2002	16.7 (6.2 to 16.9)	62	54	34	20	36	152	18,054
MRFIT	1974	1985	11.0 (8.3 to 11.8)	525	450	281	168	5	980	131,084
NCS1	1976	2012	34.8 (16.6 to 35.7)	2974	4954	3013	1901	187	8115	888,385
NCS2	1975	2012	36.0 (17.7 to 36.9)	1494	2812	1778	1006	56	4362	542,907
NCS3	1974	2012	36.9 (15.3 to 37.8)	2024	2974	1651	1241	158	5156	514,401
NPHSII	1991	2005	8.4 (4.5 to 12.0)	127	318	245	72	3	448	23,994
OSAKA	1992	2008	10.2 (3.9 to 18.8)	137	395	220	157	136	668	135,743
PRHHP	1967	1980	12.0 (5.3 to 12.0)	674	747	352	395	28	1449	99,277
PROCAM	1984	2003	8.9 (3.6 to 18.7)	330	677	452	225	52	1059	280,020
PROSPER	1998	2002	3.3 (1.8 to 3.9)	248	276	185	91	0	524	16,723
REYK	1975	2008	26.1 (6.5 to 39.1)	4590	5438	3109	2323	120	10148	466,273
RS_I	1992	2005	12.0 (2.7 to 14.3)	786	1071	589	478	251	2108	61,205
TARFS	1990	2014	20.0 (4.9 to 20.0)	340	296	70	59	23	659	69,163
WCWC	1989	2008	17.0 (5.9 to 20.2)	496	1384	818	566	354	2234	334,245
WHITEII	1987	2004	17.5 (13.9 to 18.9)	113	441	153	65	2	556	174,683
WOSCOPS	1990	1995	4.8 (3.7 to 6.0)	118	117	0	0	0	235	31,316
SUBTOTAL	1986	2014	17.5 (4.0 to 36.9)	26368	35372	18929	13866	2213	63953	4,880,270
TOTAL ERFC	1990	2015	14.9 (4.2 to 35.0)	77617	125452	67963	53047	10986	214055	17,314,610
TOTAL UKBB	2009	2020	11.8 (9.9 to 13.2)	6826	25520	17051	8469	269	32615	5,742,318
TOTAL ERFC+UKBB	2009	2020	12.5 (5.0 to 32.1)	84443	150972	85014	61516	11255	246670	23,056,928

eTable 4. Outcome definitions used in current analyses

(a) Definitions of main outcomes

Outcome	ICD-10 codes
All-cause mortality	A00 – R99
Cardiovascular disease (CVD) mortality	G45, I01, I03 – I82, I87, I95 – I99, F01, Q20 – Q28, R96
Cancer mortality	C00 – C97, D00 – D48
Non-CVD, non-cancer mortality	A00 – A99, B00 – B99, D50 – D99, E00 – E99, F00, F02 – F99, G00 – G44, G46 – G99, H00 – H99, I00, I02, I83 – I86, I88 – I89, J00 – J99, K00 – K99, L00 – L99, M00 – M99, N00 – N99, O00 – O99, P00 – P99, Q00 – Q18, Q30 – Q99, S00 – S99, T00 – T99, U04, V00 – V99, W00 – W99, X00 – X99, Y00 – Y99, Z00 – Z99
Unknown/Ill-defined mortality†	R00 – R95, R97 – R99

(b) Definitions of cause-specific non-CVD mortality

Outcome	ICD-10 codes
**Cancer mortality**	
Digestive related cancer	C15-C26
Lung cancer	C34
Genitourinary related cancer	C51-C68
Breast cancer	C50
**Non-CVD, non-cancer mortality**	
Respiratory system disease	J00-J99
External (violence/suicide/trauma)	S00-S99, T00-T98, U04, V01-V99, W00-W99, X00-X99, Y00-Y98, Z00-Z99
Nervous system disorder	F00, F02, F03, G00-G44, G46-G99
Digestive system disease	K00-K69, K78-K93
Liver disease	B15-B19, K70-K77
Mental disorder	F04-F99
Infectious/bacterial/parasitic disease	A00-A99, B00-B14, B20-B99, U07
Renal disease	N00-N19

Note: Attribution of death refers to the primary cause (or, in its absence, the underlying cause) provided by individual studies. Corresponding ICD-9 codes were used in studies that recorded outcomes using this earlier version of ICD.

†Ill-defined causes of death were non-vascular deaths defined according to study-specific read-codes for mortality, and not the standard ICD codes.

eTable 5. Hazard ratios for all-cause and cause-specific mortality per decade earlier age at diagnosis of diabetes without and with adjustment for markers of glycemia, renal function, inflammation and lipids.

Outcome \ Adjustment*	Cohorts	Participants	Events	HR (95% CI)	p	I^2 (95% CI)
**All-cause mortality**						
Adjusted for conventional risk factors	54	258179	57568	1.14 (1.05, 1.23)	0.001	67 (56, 75)
Plus fasting glucose	54	258179	57568	1.10 (1.01, 1.19)	0.027	65 (53, 74)
Adjusted for conventional risk factors	27	584337	51356	1.20 (1.12, 1.29)	<0.0001	74 (62, 82)
Plus HbA1c	27	584337	51356	1.13 (1.05, 1.20)	<0.001	65 (48, 77)
Adjusted for conventional risk factors	40	709009	84134	1.12 (1.04, 1.22)	0.003	78 (70, 84)
Plus log eGFR	40	709009	84134	1.11 (1.03, 1.20)	0.008	78 (70, 83)
Adjusted for conventional risk factors	37	624554	59810	1.13 (1.04, 1.24)	0.004	71 (60, 79)
Plus log CRP	37	624554	59810	1.14 (1.04, 1.24)	0.003	71 (60, 79)
Adjusted for conventional risk factors	61	771173	78258	1.15 (1.08, 1.23)	<0.0001	69 (60, 76)
Plus non-HDL, HDL, triglycerides†	61	771173	78258	1.15 (1.07, 1.23)	<0.0001	70 (61, 77)
**CVD mortality**						
Adjusted for conventional risk factors	51	256898	21704	1.24 (1.12, 1.36)	<0.0001	50 (31, 64)
Plus fasting glucose	51	256898	21704	1.19 (1.09, 1.31)	<0.001	41 (17, 58)
Adjusted for conventional risk factors	27	584337	13745	1.27 (1.15, 1.40)	<0.0001	64 (46, 76)
Plus HbA1c	27	584337	13745	1.18 (1.08, 1.28)	<0.001	46 (14, 65)
Adjusted for conventional risk factors	37	707336	25841	1.19 (1.07, 1.32)	0.001	69 (57, 78)
Plus log eGFR	37	707336	25841	1.18 (1.06, 1.31)	0.002	68 (55, 77)
Adjusted for conventional risk factors	34	620848	17108	1.21 (1.07, 1.38)	0.003	67 (52, 77)
Plus log CRP	34	620848	17108	1.22 (1.07, 1.38)	0.003	68 (54, 77)
Adjusted for conventional risk factors	59	770091	24027	1.20 (1.09, 1.32)	<0.001	59 (46, 69)
Plus non-HDL, HDL, triglycerides†	59	770091	24027	1.20 (1.09, 1.32)	<0.001	60 (46, 70)
**Cancer mortality**						
Adjusted for conventional risk factors	50	242787	18144	0.91 (0.80, 1.03)	0.135	34 (7, 54)
Plus fasting glucose	50	242787	18144	0.89 (0.80, 1.00)	0.059	23 (0, 46)
Adjusted for conventional risk factors	25	567738	21919	1.01 (0.92, 1.11)	0.863	33 (0, 59)
Plus HbA1c	25	567738	21919	0.96 (0.88, 1.04)	0.307	17 (0, 49)
Adjusted for conventional risk factors	36	693693	32747	0.93 (0.86, 1.00)	0.059	11 (0, 41)
Plus log eGFR	36	693693	32747	0.91 (0.84, 0.99)	0.033	16 (0, 45)
Adjusted for conventional risk factors	28	579753	24117	0.97 (0.85, 1.10)	0.589	37 (1, 60)
Plus log CRP	28	579753	24117	0.97 (0.86, 1.10)	0.651	34 (0, 59)
Adjusted for conventional risk factors	55	733508	30014	0.97 (0.88, 1.07)	0.607	31 (3, 51)
Plus non-HDL, HDL, triglycerides†	55	733508	30014	0.97 (0.88, 1.07)	0.598	31 (3, 50)
**Non-CVD, non-cancer mortality**						
Adjusted for conventional risk factors	45	239784	14248	1.19 (1.07, 1.31)	<0.001	40 (14, 58)
Plus fasting glucose	45	239784	14248	1.15 (1.03, 1.27)	0.010	41 (15, 58)
Adjusted for conventional risk factors	24	566615	13878	1.25 (1.13, 1.39)	<0.0001	61 (39, 75)
Plus HbA1c	24	566615	13878	1.19 (1.08, 1.31)	<0.001	50 (19, 69)
Adjusted for conventional risk factors	34	692544	21768	1.18 (1.06, 1.31)	0.002	63 (47, 75)
Plus log eGFR	34	692544	21768	1.16 (1.05, 1.29)	0.004	62 (45, 74)
Adjusted for conventional risk factors	26	576653	15342	1.13 (1.00, 1.28)	0.047	56 (32, 72)
Plus log CRP	26	576653	15342	1.14 (1.01, 1.28)	0.035	55 (30, 71)
Adjusted for conventional risk factors	51	725027	19965	1.21 (1.12, 1.32)	<0.0001	39 (14, 57)
Plus non-HDL, HDL, triglycerides†	51	725027	19965	1.21 (1.11, 1.31)	<0.0001	39 (14, 56)

* Conventional risk factors included: cohort, sex, age, smoking status, BMI, systolic blood pressure, and total cholesterol. Fasting glucose, HbA1c, and log eGFR were adjusted for as a continuous variables with linear and quadratic terms.

† Replacing total cholesterol with the trio of non-HDL cholesterol, HDL cholesterol, and log triglycerides.

eTable 6. Assessment of additive interactions by category of age at diagnosis of diabetes (<50 years vs >50 years) for all-cause mortality risk adjusted for conventional risk factors*.

		Diabetes diagnosed < 50 years		Diabetes diagnosed ≥ 50 years	
Characteristic \ Subgroup	Statistic	Statistic (95% CI)	p	Statistic (95% CI)	p
Sex					
Male vs Female sex (without diabetes)	RR	1.63 (1.58, 1.68)	<0.0001	1.63 (1.58, 1.68)	<0.0001
With diabetes AND Male sex	RR	2.57 (2.35, 2.81)	<0.0001	1.66 (1.56, 1.77)	<0.0001
With diabetes AND Female sex	RR	3.38 (3.10, 3.69)	<0.0001	2.49 (2.34, 2.65)	<0.0001
Relative excess risk due to interaction (RERI)	RERI_RR	0.186 (-0.066, 0.438)	0.148	0.196 (0.105, 0.287)	<0.0001
Attributable proportion (AP)	AP	0.055 (-0.017, 0.127)	0.132	0.079 (0.045, 0.113)	<0.0001
Synergy index (SI)	SI	1.08 (0.97, 1.21)	0.142	1.15 (1.08, 1.23)	<0.0001
Smoking status					
Current vs Other status (without diabetes)	RR	1.96 (1.87, 2.05)	<0.0001	1.96 (1.87, 2.05)	<0.0001
With diabetes AND Other status	RR	2.39 (2.21, 2.59)	<0.0001	1.61 (1.52, 1.70)	<0.0001
With diabetes AND Current smoker	RR	3.89 (3.47, 4.36)	<0.0001	2.73 (2.52, 2.95)	<0.0001
Relative excess risk due to interaction (RERI)	RERI_RR	0.542 (0.192, 0.892)	0.002	0.164 (0.011, 0.316)	0.036
Attributable proportion (AP)	AP	0.139 (0.063, 0.216)	<0.001	0.060 (0.008, 0.112)	0.025
Synergy index (SI)	SI	1.23 (1.09, 1.39)	0.001	1.10 (1.01, 1.21)	0.027
Age at risk					
Age ≥ 60 vs Age < 60 years (without diabetes)	RR	3.95 (3.51, 4.44)	<0.0001	3.95 (3.51, 4.44)	<0.0001
With diabetes AND Age < 60 years	RR	2.35 (2.11, 2.61)	<0.0001	2.88 (2.52, 3.29)	<0.0001
With diabetes AND Age ≥ 60 years	RR	6.85 (5.90, 7.96)	<0.0001	6.48 (5.60, 7.50)	<0.0001
Relative excess risk due to interaction (RERI)	RERI_RR	1.555 (0.851, 2.259)	<0.0001	0.653 (0.242, 1.063)	0.002
Attributable proportion (AP)	AP	0.227 (0.148, 0.305)	<0.0001	0.101 (0.046, 0.155)	<0.001
Synergy index (SI)	SI	1.36 (1.21, 1.53)	<0.0001	1.14 (1.06, 1.22)	0.001
History of CVD (HxCVD)					
HxCVD=Yes vs HxCVD=No (without diabetes)	RR	1.67 (1.59, 1.74)	<0.0001	1.67 (1.59, 1.74)	<0.0001
With diabetes AND HxCVD=No	RR	2.20 (2.04, 2.38)	<0.0001	1.55 (1.47, 1.64)	<0.0001
With diabetes AND HxCVD=Yes	RR	3.82 (3.35, 4.35)	<0.0001	2.36 (2.18, 2.55)	<0.0001
Relative excess risk due to interaction (RERI)	RERI_RR	0.945 (0.513, 1.377)	<0.0001	0.138 (0.016, 0.260)	0.027
Attributable proportion (AP)	AP	0.248 (0.163, 0.332)	<0.0001	0.059 (0.010, 0.107)	0.018
Synergy index (SI)	SI	1.51 (1.29, 1.76)	<0.0001	1.11 (1.02, 1.22)	0.019

* Conventional risk factors included: cohort, sex, age, smoking status, BMI, systolic blood pressure, and total cholesterol.

For each subgroup characteristic, the table summarises (a) relative risks (RR) for groups of the characteristic with/without diabetes diagnosed before and after age 50 years; and (b) summary indices of additive interactions that are functions of the preceding relative risks (for details see VanderWeele TJ et al A Tutorial on Interaction Epidemiol Methods 2014(3)33–72).

eTable 7. Hazard ratios for all-cause and cause-specific mortality according to duration of diabetes with adjustment for conventional risk factors*

		HR (95% CI) adjusted for…		
Outcome \ Duration of diabetes	Events	Age and sex	Age, sex, and smoking	Age, sex, smoking, and other risk factors*
**All-cause mortality**				
No Diabetes	153068	1 (Ref)	1 (Ref)	1 (Ref)
<5 yrs	3908	1.58 (1.45, 1.73)	1.62 (1.49, 1.76)	1.58 (1.45, 1.72)
5 to <10 yrs	2867	1.56 (1.46, 1.67)	1.60 (1.50, 1.71)	1.54 (1.45, 1.64)
10 to <15 yrs	2657	1.69 (1.57, 1.82)	1.72 (1.60, 1.85)	1.66 (1.56, 1.78)
15 to <20yrs	2029	2.00 (1.86, 2.14)	2.04 (1.90, 2.18)	1.94 (1.82, 2.06)
≥20 yrs	2633	2.01 (1.85, 2.19)	2.07 (1.90, 2.25)	2.00 (1.84, 2.16)
**Per decade higher**	**167162**	**1.14 (1.08, 1.19)**	**1.13 (1.08, 1.19)**	**1.13 (1.07, 1.19)**
P-value		<0.0001	<0.0001	<0.0001
I2 (95% CI)		67 (59, 74)	68 (60, 74)	67 (60, 74)
**CVD mortality**				
No Diabetes	53857	1 (Ref)	1 (Ref)	1 (Ref)
<5 yrs	1538	1.85 (1.66, 2.06)	1.89 (1.70, 2.10)	1.73 (1.56, 1.92)
5 to <10 yrs	1097	2.01 (1.81, 2.22)	2.06 (1.85, 2.29)	1.89 (1.72, 2.09)
10 to <15 yrs	988	2.13 (1.91, 2.38)	2.17 (1.95, 2.42)	1.98 (1.80, 2.18)
15 to <20yrs	817	2.61 (2.33, 2.92)	2.66 (2.38, 2.97)	2.40 (2.19, 2.63)
≥20 yrs	1054	2.56 (2.25, 2.92)	2.63 (2.31, 2.99)	2.41 (2.15, 2.69)
**Per decade higher**	**59351**	**1.19 (1.11, 1.27)**	**1.19 (1.11, 1.27)**	**1.20 (1.12, 1.28)**
P-value		<0.0001	<0.0001	<0.0001
I2 (95% CI)		58 (47, 67)	58 (48, 67)	59 (48, 67)
**Cancer mortality**				
No Diabetes	53217	1 (Ref)	1 (Ref)	1 (Ref)
<5 yrs	1063	1.40 (1.27, 1.55)	1.43 (1.30, 1.57)	1.42 (1.29, 1.55)
5 to <10 yrs	816	1.27 (1.17, 1.38)	1.29 (1.19, 1.40)	1.24 (1.15, 1.33)
10 to <15 yrs	760	1.43 (1.28, 1.60)	1.46 (1.32, 1.62)	1.43 (1.33, 1.54)
15 to <20yrs	431	1.34 (1.20, 1.50)	1.37 (1.24, 1.52)	1.31 (1.19, 1.44)
≥20 yrs	469	1.26 (1.13, 1.40)	1.30 (1.17, 1.43)	1.24 (1.13, 1.36)
**Per decade higher**	**56756**	**0.95 (0.89, 1.02)**	**0.95 (0.89, 1.02)**	**0.95 (0.89, 1.01)**
P-value		0.154	0.143	0.113
I2 (95% CI)		16 (0, 37)	15 (0, 36)	17 (0, 37)
**Non-CVD, non-cancer mortality**				
No Diabetes	35986	1 (Ref)	1 (Ref)	1 (Ref)
<5 yrs	954	1.84 (1.61, 2.10)	1.87 (1.64, 2.13)	1.92 (1.68, 2.19)
5 to <10 yrs	764	1.79 (1.61, 1.99)	1.83 (1.65, 2.03)	1.82 (1.65, 2.00)
10 to <15 yrs	775	2.03 (1.84, 2.24)	2.07 (1.88, 2.28)	2.02 (1.84, 2.21)
15 to <20yrs	696	2.73 (2.49, 2.99)	2.78 (2.54, 3.04)	2.64 (2.42, 2.87)
≥20 yrs	984	2.76 (2.43, 3.13)	2.85 (2.50, 3.24)	2.82 (2.49, 3.19)
**Per decade higher**	**40159**	**1.18 (1.10, 1.27)**	**1.18 (1.10, 1.27)**	**1.16 (1.08, 1.25)**
P-value		<0.0001	<0.0001	<0.0001
I2 (95% CI)		48 (32, 60)	49 (33, 61)	48 (33, 60)

*Analyses based on ERFC and UK Biobank, including 92 cohorts and 1,132,277 participants with complete information on age at diagnosis of diabetes, age, sex, smoking and other risk factors.

* Other risk factors were body mass index, systolic blood pressure and total cholesterol.

eTable 8. Hazard ratios for all-cause and cause-specific mortality per decade higher duration of diabetes without and with adjustment for markers of glycemia, renal function, inflammation and lipids.

Outcome \ Adjustment*	Cohorts	Participants	Events	HR (95% CI)	p	I^2 (95% CI)
**All-cause mortality**						
Adjusted for conventional risk factors	54	258179	57568	1.14 (1.06, 1.24)	<0.001	66 (55, 74)
Plus fasting glucose	54	258179	57568	1.10 (1.01, 1.19)	0.021	63 (51, 72)
Adjusted for conventional risk factors	27	584337	51356	1.20 (1.12, 1.29)	<0.0001	74 (63, 82)
Plus HbA1c	27	584337	51356	1.13 (1.05, 1.20)	<0.001	66 (48, 77)
Adjusted for conventional risk factors	40	709009	84134	1.12 (1.04, 1.22)	0.003	78 (70, 84)
Plus log eGFR	40	709009	84134	1.11 (1.03, 1.20)	0.008	78 (70, 83)
Adjusted for conventional risk factors	37	624554	59810	1.13 (1.04, 1.24)	0.004	71 (60, 79)
Plus log CRP	37	624554	59810	1.14 (1.04, 1.24)	0.003	71 (60, 79)
Adjusted for conventional risk factors	61	771173	78258	1.15 (1.08, 1.23)	<0.0001	69 (60, 76)
Plus non-HDL, HDL, triglycerides†	61	771173	78258	1.15 (1.07, 1.23)	<0.0001	70 (61, 77)
**CVD mortality**						
Adjusted for conventional risk factors	51	256898	21704	1.24 (1.12, 1.36)	<0.0001	49 (30, 63)
Plus fasting glucose	51	256898	21704	1.19 (1.09, 1.31)	<0.001	40 (17, 58)
Adjusted for conventional risk factors	27	584337	13745	1.27 (1.15, 1.40)	<0.0001	64 (45, 76)
Plus HbA1c	27	584337	13745	1.18 (1.09, 1.28)	<0.001	45 (14, 65)
Adjusted for conventional risk factors	37	707336	25841	1.19 (1.07, 1.32)	0.001	69 (57, 78)
Plus log eGFR	37	707336	25841	1.18 (1.06, 1.31)	0.002	68 (55, 77)
Adjusted for conventional risk factors	34	620848	17108	1.20 (1.06, 1.36)	0.004	65 (50, 76)
Plus log CRP	34	620848	17108	1.21 (1.06, 1.37)	0.003	66 (51, 76)
Adjusted for conventional risk factors	59	770091	24027	1.19 (1.09, 1.31)	<0.001	59 (45, 69)
Plus non-HDL, HDL, triglycerides†	59	770091	24027	1.19 (1.09, 1.32)	<0.001	59 (45, 69)
**Cancer mortality**						
Adjusted for conventional risk factors	50	242787	18144	0.92 (0.80, 1.04)	0.184	36 (10, 55)
Plus fasting glucose	50	242787	18144	0.90 (0.79, 1.02)	0.089	30 (1, 51)
Adjusted for conventional risk factors	25	567738	21919	1.01 (0.92, 1.11)	0.859	35 (0, 60)
Plus HbA1c	25	567738	21919	0.96 (0.88, 1.05)	0.351	20 (0, 51)
Adjusted for conventional risk factors	36	693693	32747	0.93 (0.86, 1.00)	0.060	10 (0, 40)
Plus log eGFR	36	693693	32747	0.92 (0.84, 0.99)	0.034	15 (0, 44)
Adjusted for conventional risk factors	28	579753	24117	0.97 (0.86, 1.10)	0.626	32 (0, 57)
Plus log CRP	28	579753	24117	0.98 (0.87, 1.10)	0.699	29 (0, 55)
Adjusted for conventional risk factors	55	733508	30014	0.98 (0.89, 1.07)	0.620	28 (0, 49)
Plus non-HDL, HDL, triglycerides†	55	733508	30014	0.98 (0.89, 1.07)	0.610	28 (0, 48)
**Non-CVD, non-cancer mortality**						
Adjusted for conventional risk factors	44	231659	14222	1.19 (1.07, 1.31)	<0.001	41 (15, 59)
Plus fasting glucose	44	231659	14222	1.15 (1.03, 1.28)	0.011	42 (16, 59)
Adjusted for conventional risk factors	24	566615	13878	1.25 (1.13, 1.39)	<0.0001	61 (39, 75)
Plus HbA1c	24	566615	13878	1.19 (1.08, 1.31)	<0.001	49 (18, 68)
Adjusted for conventional risk factors	34	692544	21768	1.18 (1.06, 1.31)	0.002	63 (47, 75)
Plus log eGFR	34	692544	21768	1.17 (1.05, 1.29)	0.004	62 (45, 74)
Adjusted for conventional risk factors	26	576653	15342	1.13 (1.00, 1.28)	0.045	56 (31, 72)
Plus log CRP	26	576653	15342	1.14 (1.01, 1.28)	0.033	55 (29, 71)
Adjusted for conventional risk factors	51	725027	19965	1.21 (1.12, 1.32)	<0.0001	39 (14, 56)
Plus non-HDL, HDL, triglycerides†	51	725027	19965	1.21 (1.11, 1.31)	<0.0001	38 (13, 56)

* Conventional risk factors included: cohort, sex, age, smoking status, BMI, systolic blood pressure, and total cholesterol. Fasting glucose, HbA1c, and log eGFR were adjusted for as a continuous variables with linear and quadratic terms.

† Replacing total cholesterol with the trio of non-HDL cholesterol, HDL cholesterol, and log triglycerides.

eTable 9. Hazard ratios for components of non-CVD mortality per decade earlier age at diagnosis of diabetes adjusted for conventional risk factors*.

Outcome	Cohorts	Participants	Events	HR (95% CI)	p	I^2 (95% CI)
**Cancer mortality**						
All cancer mortality	83	1068608	56756	0.94 (0.88, 1.02)	0.128	24 (0, 43)
Digestive related cancer	70	1014958	15871	0.97 (0.87, 1.09)	0.646	12 (0, 35)
Lung cancer	63	954825	11687	0.93 (0.83, 1.04)	0.195	0 (0, 30)
Genitourinary related cancer	64	999797	9430	0.95 (0.84, 1.07)	0.389	0 (0, 30)
Breast cancer	33	855481	3197	1.07 (0.87, 1.32)	0.516	0 (0, 39)
**Non-CVD, non-cancer mortality**						
All non-CVD, non-cancer mortality	80	1061784	40159	1.16 (1.08, 1.25)	<0.0001	50 (36, 62)
Respiratory system disease	69	1027860	12853	1.07 (0.96, 1.19)	0.232	19 (0, 40)
External (violence/suicide/trauma)	63	1022280	5710	1.21 (1.04, 1.42)	0.015	3 (0, 25)
Nervous system disorder	39	838787	5792	1.13 (1.01, 1.28)	0.041	0 (0, 37)
Digestive system disease	48	926328	3149	1.20 (1.03, 1.40)	0.020	0 (0, 34)
Liver disease	33	829812	1699	0.77 (0.62, 0.96)	0.022	0 (0, 39)
Mental disorder	27	759888	1092	1.10 (0.84, 1.44)	0.486	0 (0, 43)
Infectious/bacterial/parasitic disease	33	838853	2162	1.28 (1.07, 1.53)	0.007	13 (0, 44)
Renal disease	25	731736	983	1.46 (1.16, 1.84)	0.001	29 (0, 57)

* Conventional risk factors included: cohort, sex, age, smoking status, BMI, systolic blood pressure, and total cholesterol. Studies with fewer than 10 events of any outcome were excluded from the analysis of that outcome.

eTable 10. Hazard ratios (HR) and subdistribution hazard ratios (SHRs) for all-cause and cause-specific mortality per decade earlier age at diagnosis of diabetes adjusted for conventional risk factors* with/without adjustment for competing risks in analyses restricted to studies with ≥80 deaths recorded$.

Outcome	Cohorts‡	Participants	Main events	Competing events†	HR (95% CI)	p	I^2 (95% CI)
**Cox model results (without competing risks)**							
All-cause mortality	87	1119110	166929	-	1.13 (1.08, 1.19)	<0.0001	69 (61, 75)
CVD mortality	76	1090897	58899	-	1.20 (1.12, 1.29)	<0.0001	65 (55, 72)
Cancer mortality	67	1030099	56143	-	0.94 (0.87, 1.01)	0.084	27 (0, 46)
Non-CVD, non-cancer mortality	57	999429	39093	-	1.15 (1.06, 1.24)	<0.001	58 (44, 69)
**Fine and Gray model results (competing risk adjusted)**	Cohorts‡	Participants	Main events	Competing events†	SHR (95% CI)	p	I^2 (95% CI)
CVD mortality	73	1078926	58340	104806	1.30 (1.22, 1.38)	<0.0001	59 (48, 67)
Cancer mortality	65	1019096	55515	101606	0.97 (0.90, 1.03)	0.287	39 (21, 54)
Non-CVD, non-cancer mortality	56	991281	38880	114155	1.24 (1.17, 1.32)	<0.0001	51 (37, 63)

* Conventional risk factors included: cohort, sex, age, smoking status, BMI, systolic blood pressure, and total cholesterol. Studies with fewer than 80 events of any outcome were excluded from the analysis of that outcome.
† Competing events in the Fine and Gray model comprised deaths other than the main cause of interest.
‡ The Fine and Gray model did not converge to a solution in a few cohorts hence the slight difference in sample sizes shown.
$ Analyses were restricted to studies with ≥80 deaths recorded as additional sensitivity analyses to check for possible overfitting according to conventional rule of 10 events per variable included in the regression model (here 8 variables x 10 events = 80 deaths).

eFigure 1. Associations of age at diagnosis of diabetes with all-cause and cause-specific mortality adjusted for age and sex.

(a) Sex adjusted associations by categories of age at diagnosis of diabetes

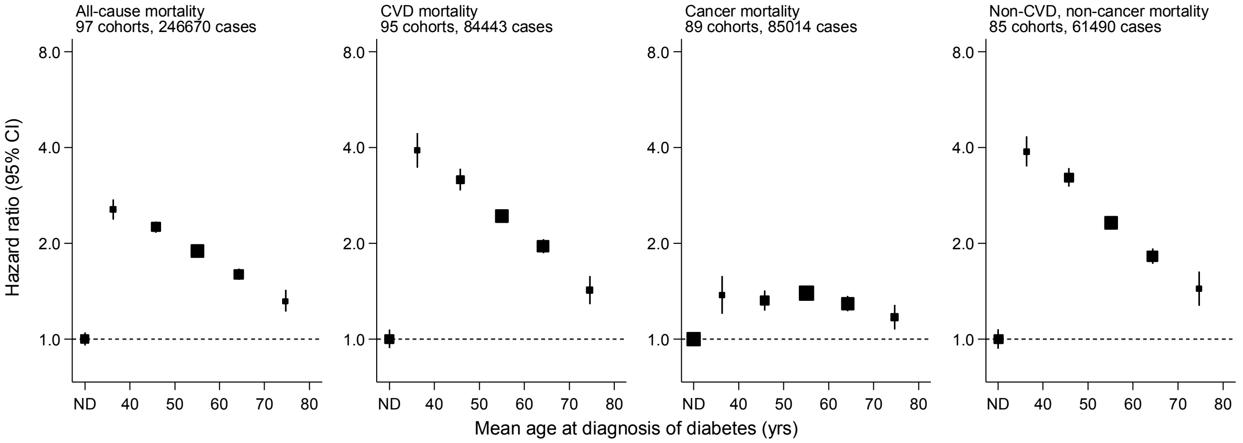

ND, No diabetes. The 6 categories of age at diagnosis correspond to: ND, 30 to <40 yrs, 40 to <50 yrs, 50 to <60 yrs, 60 to <70yrs, and ≥70 yrs. Hazard ratios adjusted for age and sex. The reference category is no diabetes. Studies with fewer than 10 events of any outcome were excluded from the analysis of that outcome. Sizes of the boxes are proportional to the inverse of the variance of the log-transformed hazard ratios. Vertical lines represent 95% CIs.

(b) Continuous associations by sex and overall using fractional polynomial modelling

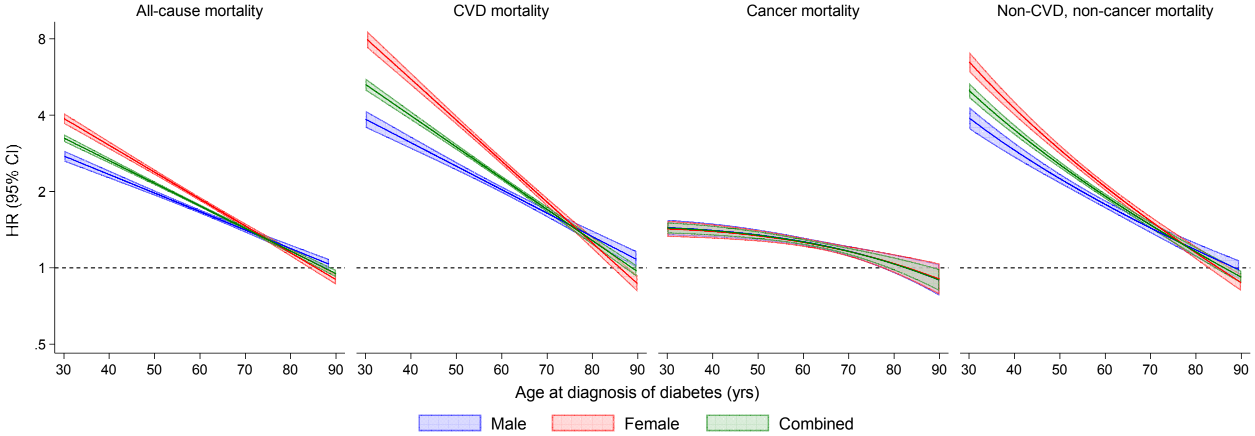

The reference group is no diabetes. Studies with fewer than 10 events of any outcome were excluded from the analysis of that outcome.

eFigure 2. Hazard ratios for all-cause mortality per decade earlier age at diagnosis of diabetes according to selected participant and study-level characteristics.

Variable\ Subgroup	No. of studies	No. of participants	No. of events	HR (95% CI)	Interaction P-value
Sex					
Male	88	585590	104678	1.12 (1.06, 1.19)	0.031
Female	71	546687	62484	1.18 (1.11, 1.24)	
Smoking status					
Other	92	911248	110486	1.14 (1.08, 1.19)	0.421
Current	91	281546	56676	1.11 (1.04, 1.19)	
Age at risk (yrs)					
<60	81	766166	29011	1.16 (1.10, 1.22)	0.024
>=60	91	802702	138004	1.14 (1.08, 1.21)	
Prior vascular disease?					
No	91	617524	115112	1.14 (1.10, 1.19)	0.014
Yes	80	49930	22198	1.16 (1.11, 1.20)	
Diabetes diagnosis available					
Prevalent and incident	45	247270	47548	1.13 (1.06, 1.22)	0.790
Prevalent only	16	172676	27478	1.17 (1.03, 1.33)	
Incident only	30	247508	62284	1.10 (0.93, 1.28)	
Median year of baseline					
<1990	42	346749	98609	1.06 (0.98, 1.16)	0.003
>=1990	49	320705	38701	1.20 (1.11, 1.30)	
Median year of follow up					
<2000	21	115710	32532	1.03 (0.91, 1.16)	0.005
>=2000	70	551744	104778	1.16 (1.09, 1.24)	

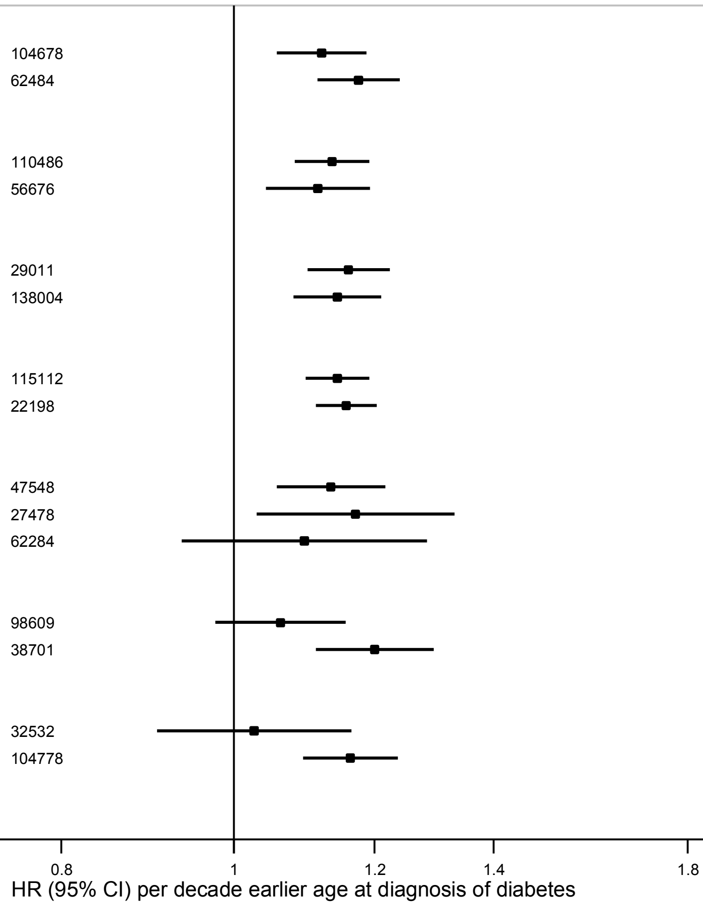

eFigure 3. Cohort-specific hazard ratios for all-cause mortality per decade earlier age at diagnosis of diabetes according to calendar time of study enrolment and follow-up period.

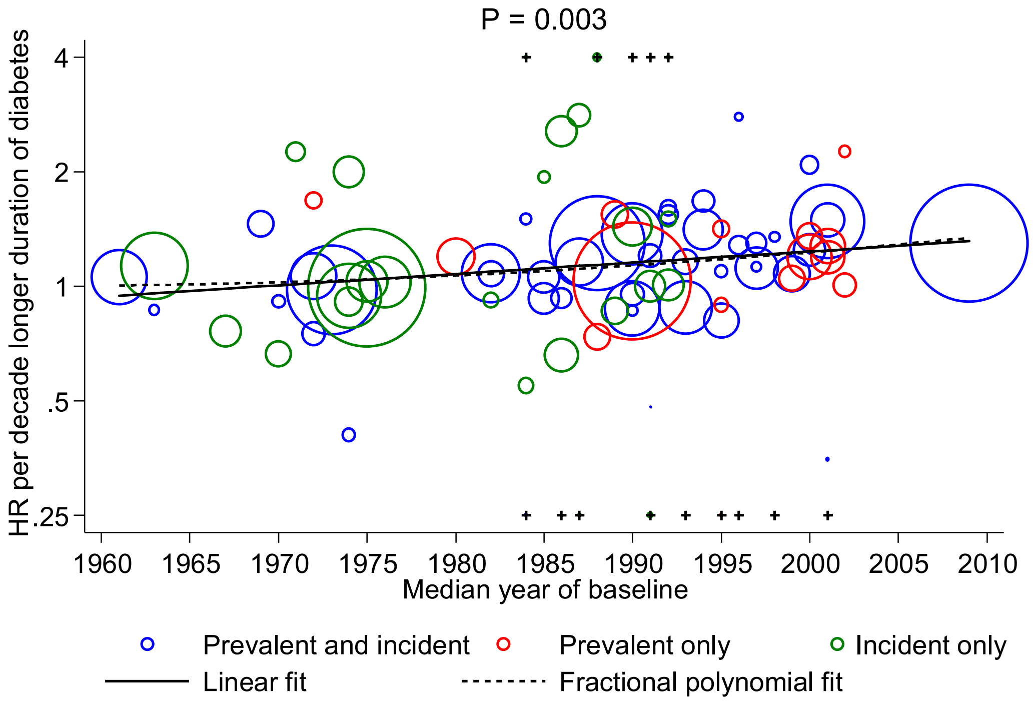

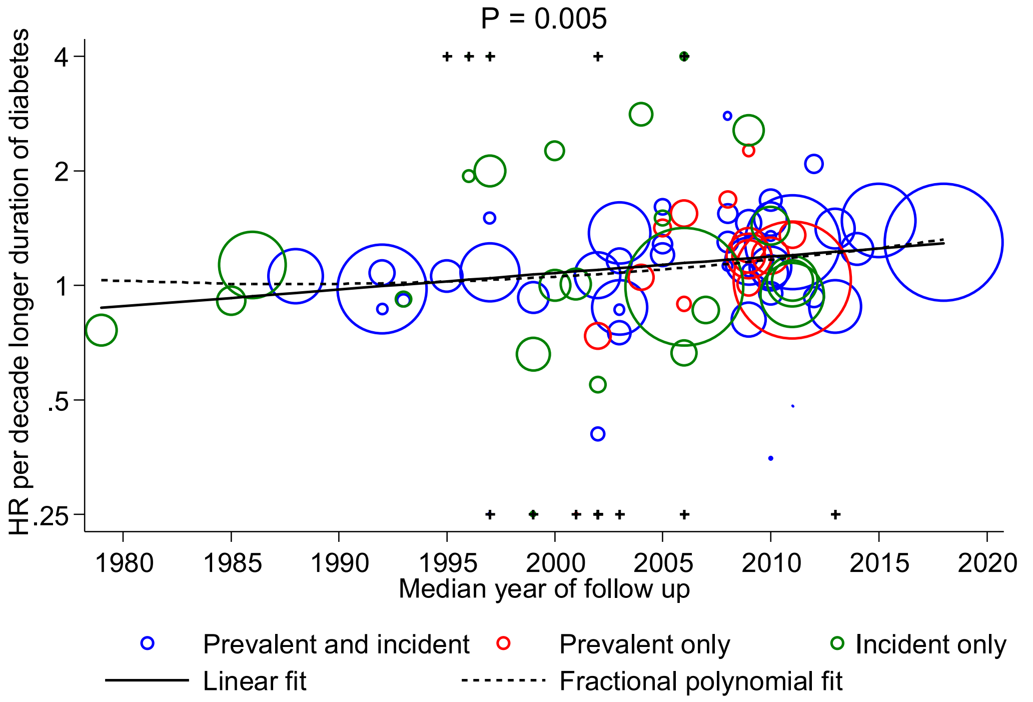

* Multivariate meta-analysis of cohort-specific hazard ratios estimated using Cox-regression models stratified by sex and adjusted for age, smoking status, BMI, systolic blood pressure, and total cholesterol. Cohort-specific hazard ratios exceeding 0.25 or 4.0 are truncated at those values and shown as plus (+) in the meta-regression plots. The line of best-fit corresponds to the meta-regression based relationship of cohort-specific log HRs and calendar time.

eFigure 4. Hazard ratios for all-cause and cause-specific mortality in ERFC and UK Biobank separately according to age at diagnosis of diabetes adjusted for age and sex.*

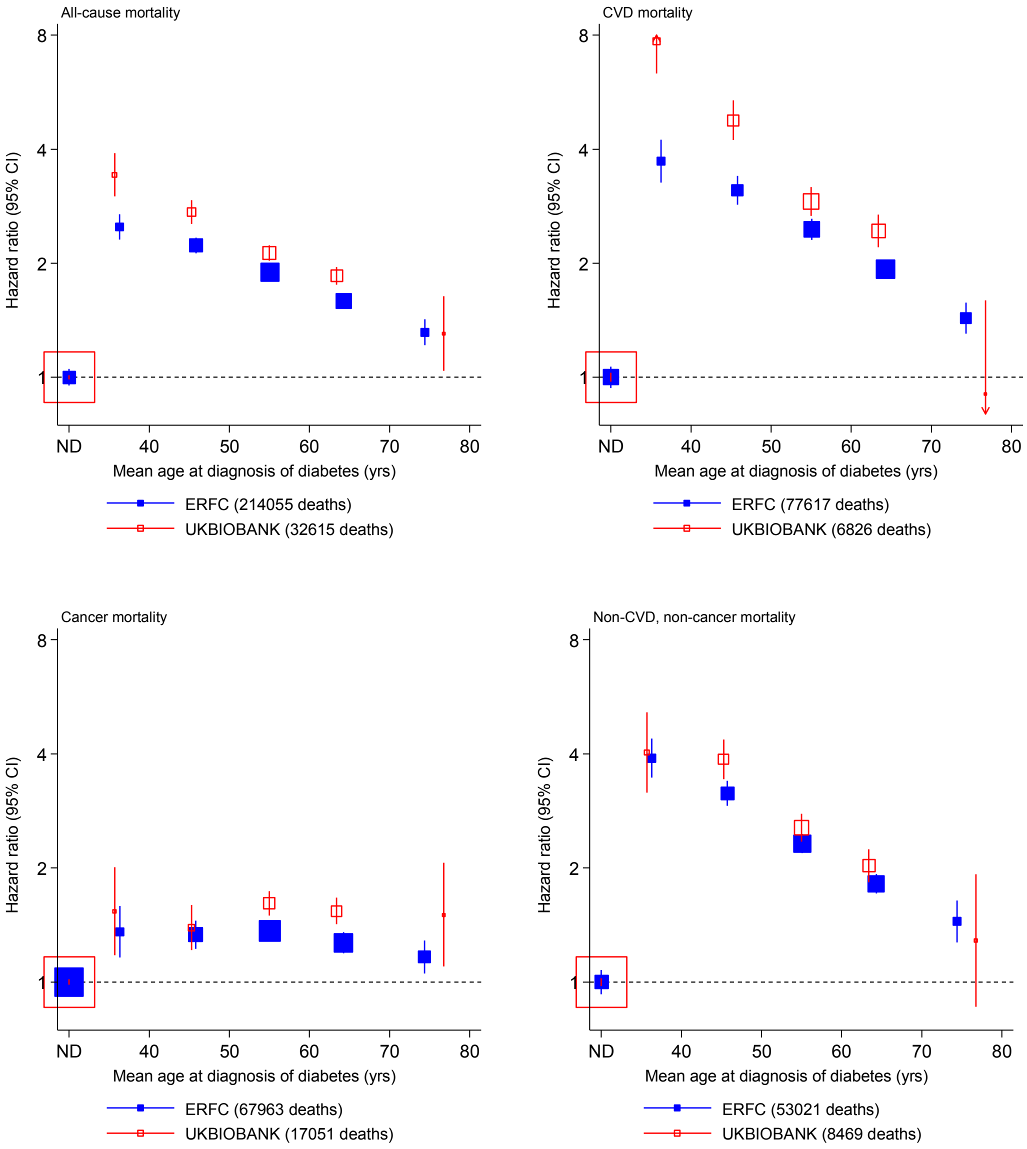

* Hazard ratios were estimated using Cox-regression models stratified by centre and sex and adjusted for age. The 5 categories of age at diagnosis correspond to: No diabetes (ND, Reference), 30 to <40 yrs, 40 to <50 yrs, 50 to <60 yrs, 60 to <70 yrs, and ≥70 yrs.

eFigure 5. Hazard ratios for all-cause and cause-specific mortality according to duration of diabetes adjusted for age and sex (top) and sex-specific (bottom).

(a) Sex adjusted and sex-specific associations by categories of age at diagnosis of diabetes

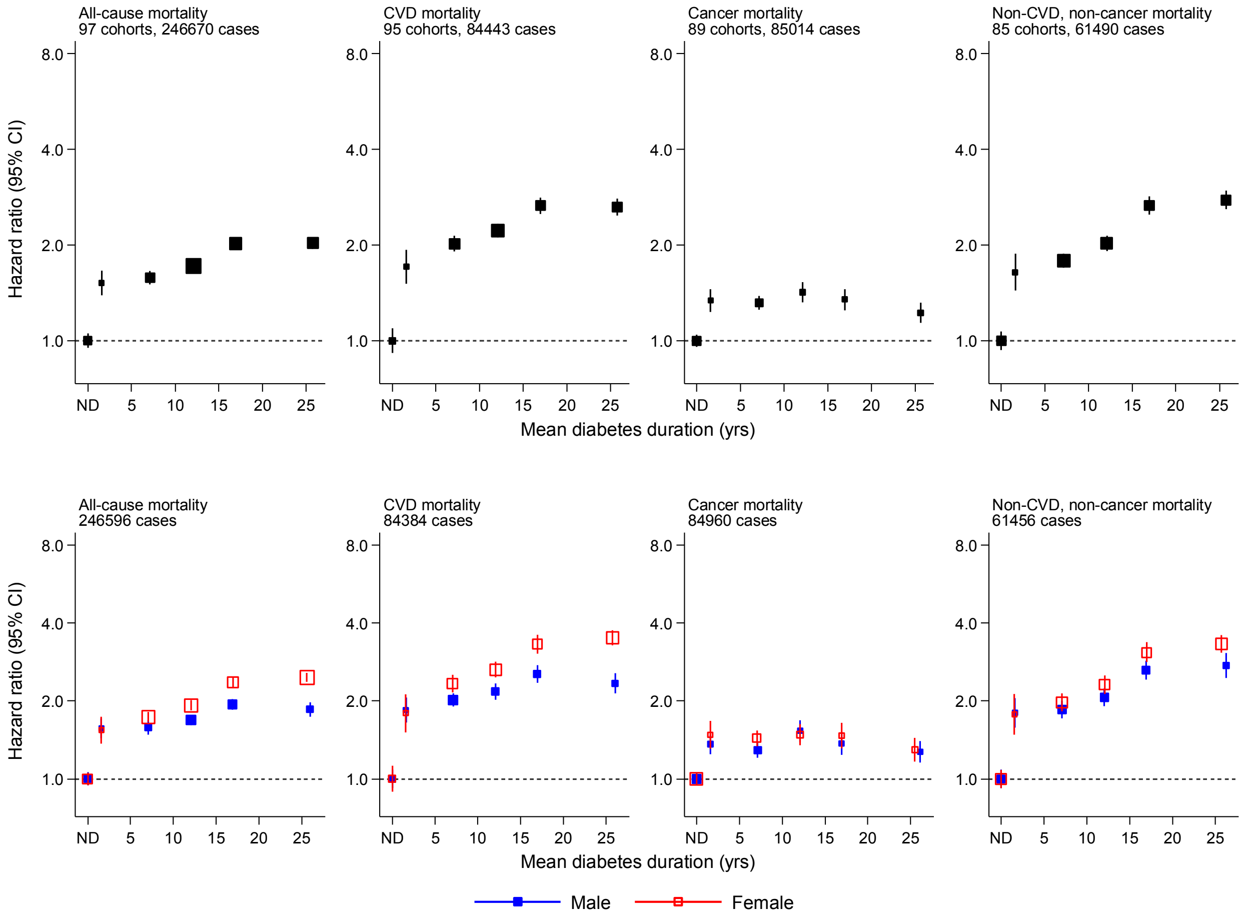

ND, No diabetes. The 6 categories of duration of diabetes correspond to: ND, <5 yrs, 5 to <10 yrs, 10 to <15 yrs, 15 to <20yrs, and ≥20 yrs. Hazard ratios adjusted for age and sex. The reference category is no diabetes. Studies with fewer than 10 events of any outcome were excluded from the analysis of that outcome. Sizes of the boxes are proportional to the inverse of the variance of the log-transformed hazard ratios. Vertical lines represent 95% CIs.

(b) Continuous associations by sex and overall using fractional polynomial modelling

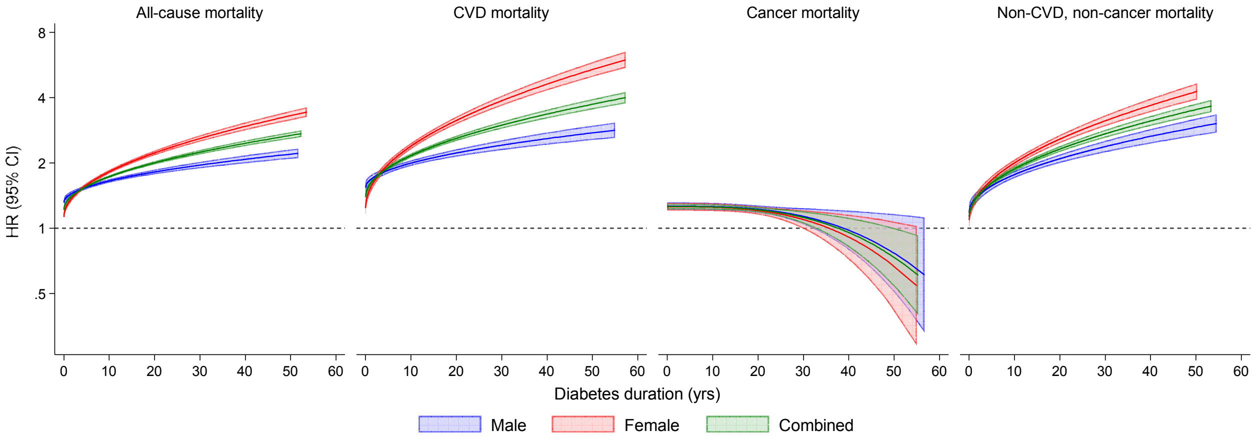

eFigure 6. Cohort specific percentages of right censoring, deaths and loss to follow up.

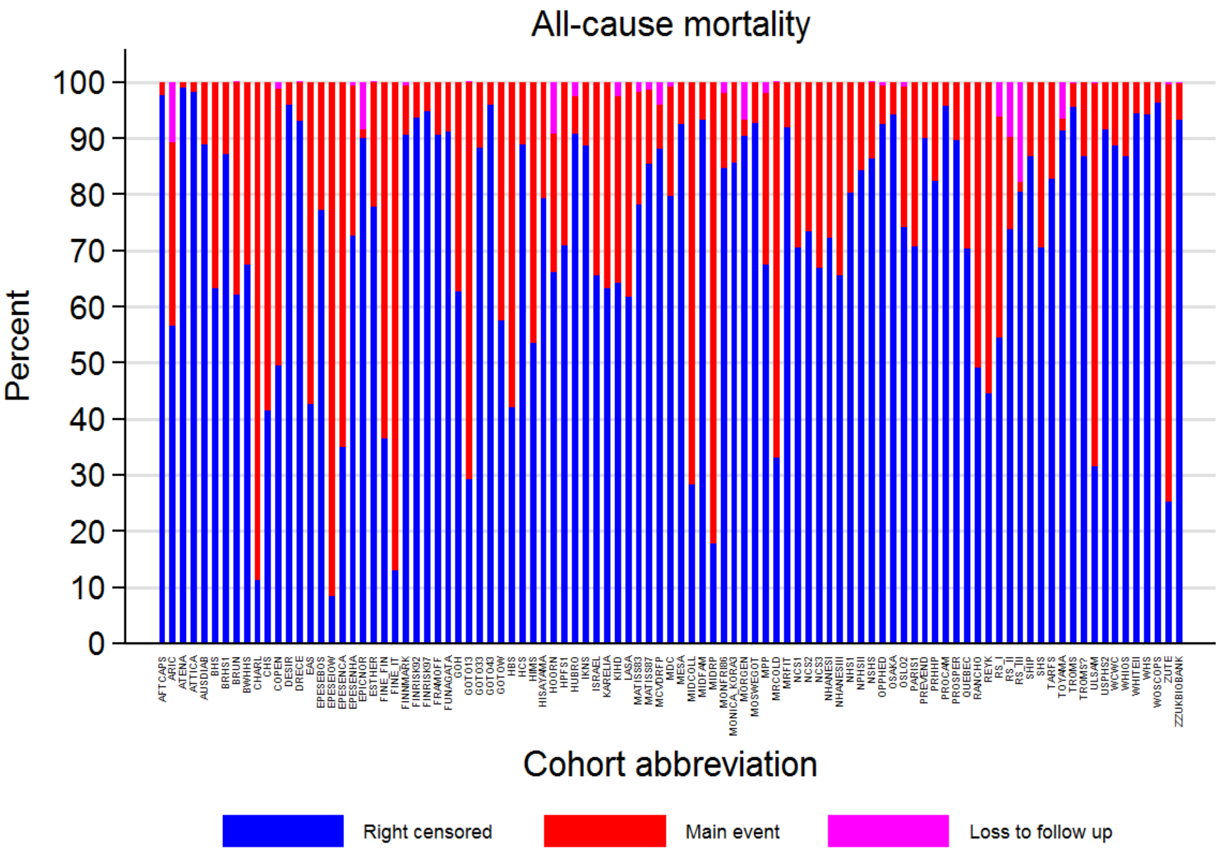

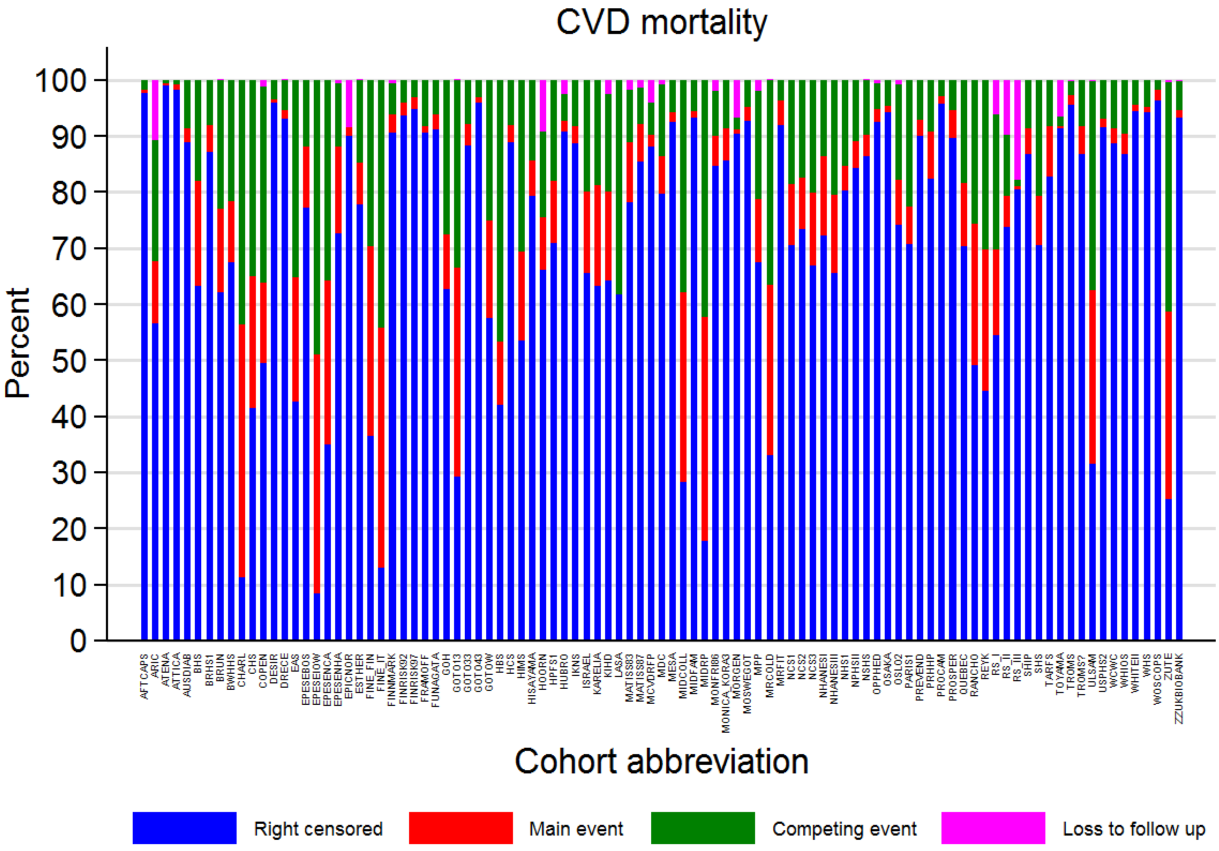

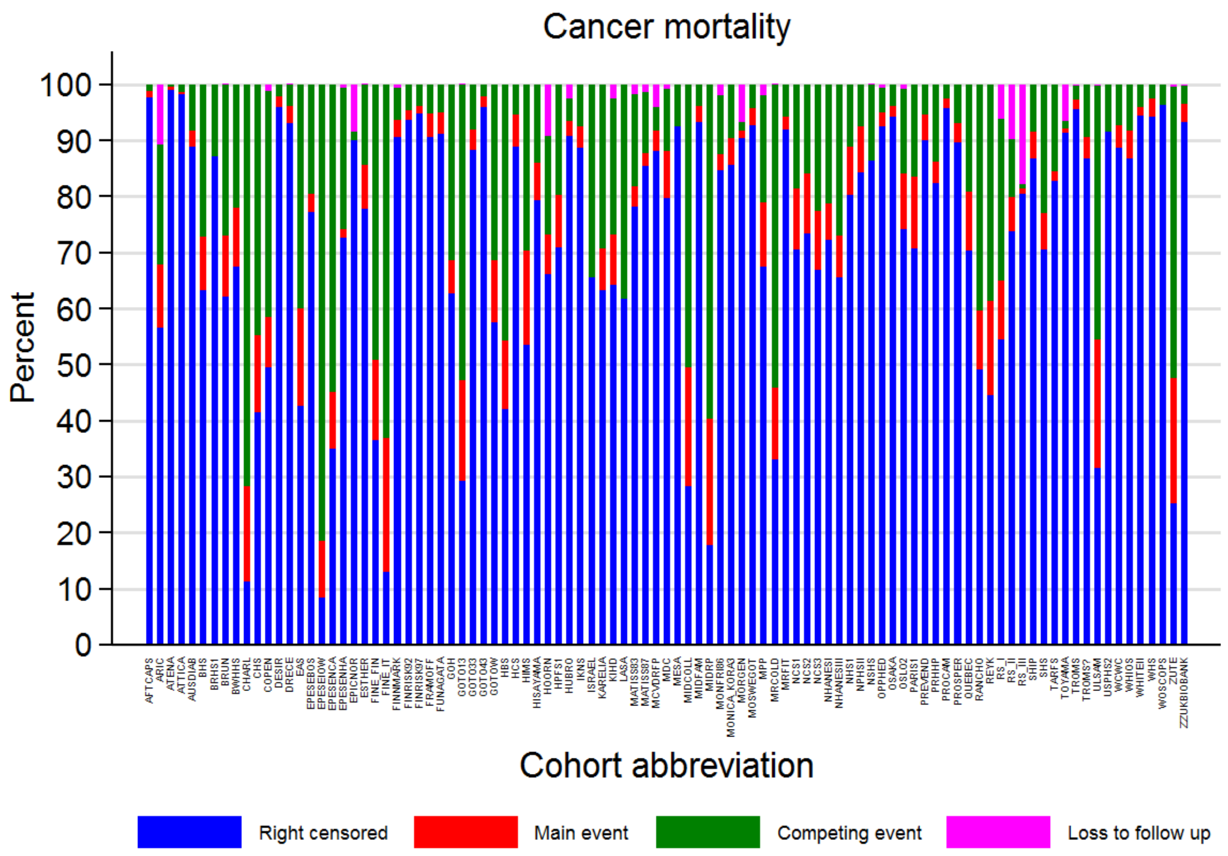

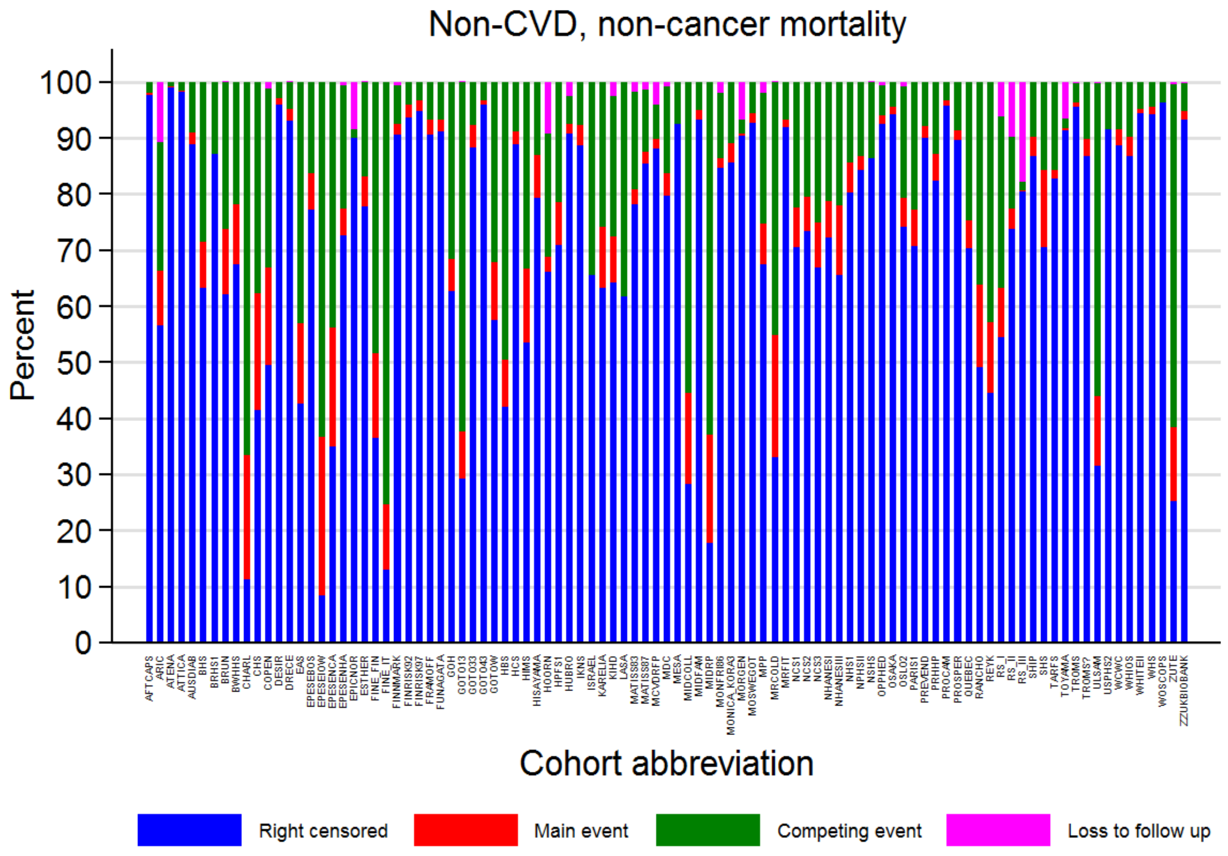

The graphs show cohort-specific percentages of participants that: (a) were still alive at the end of follow up (right censored) or (b) died of the main cause indicated on the graph title (i.e. main event) or (c) died of a different cause than one indicated (competing event) or (d) were lost to follow up (loss to follow up).

eFigure 7. Estimated cause-specific contributions to overall reduction in life expectancy by age at diagnosis of diabetes using US 2015 death rates.

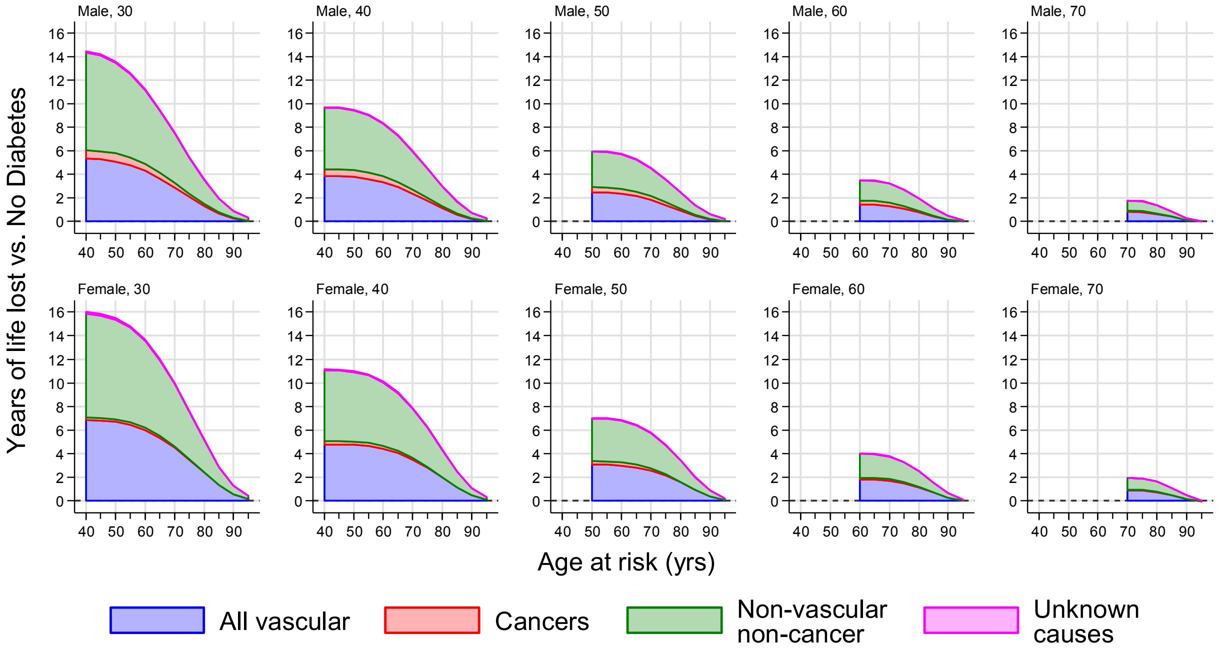

The estimates of cumulative survival from 40 years of age onwards according to age at diagnosis of diabetes were calculated by applying hazard ratios (specific to age at risk) for all-cause mortality associated with age at diagnosis of diabetes to US 2015 death rates at the age of 40 years or older.

eFigure 8. Estimated cause-specific contributions to overall reduction in life expectancy by age at diagnosis of diabetes using EU 2015 death rates.

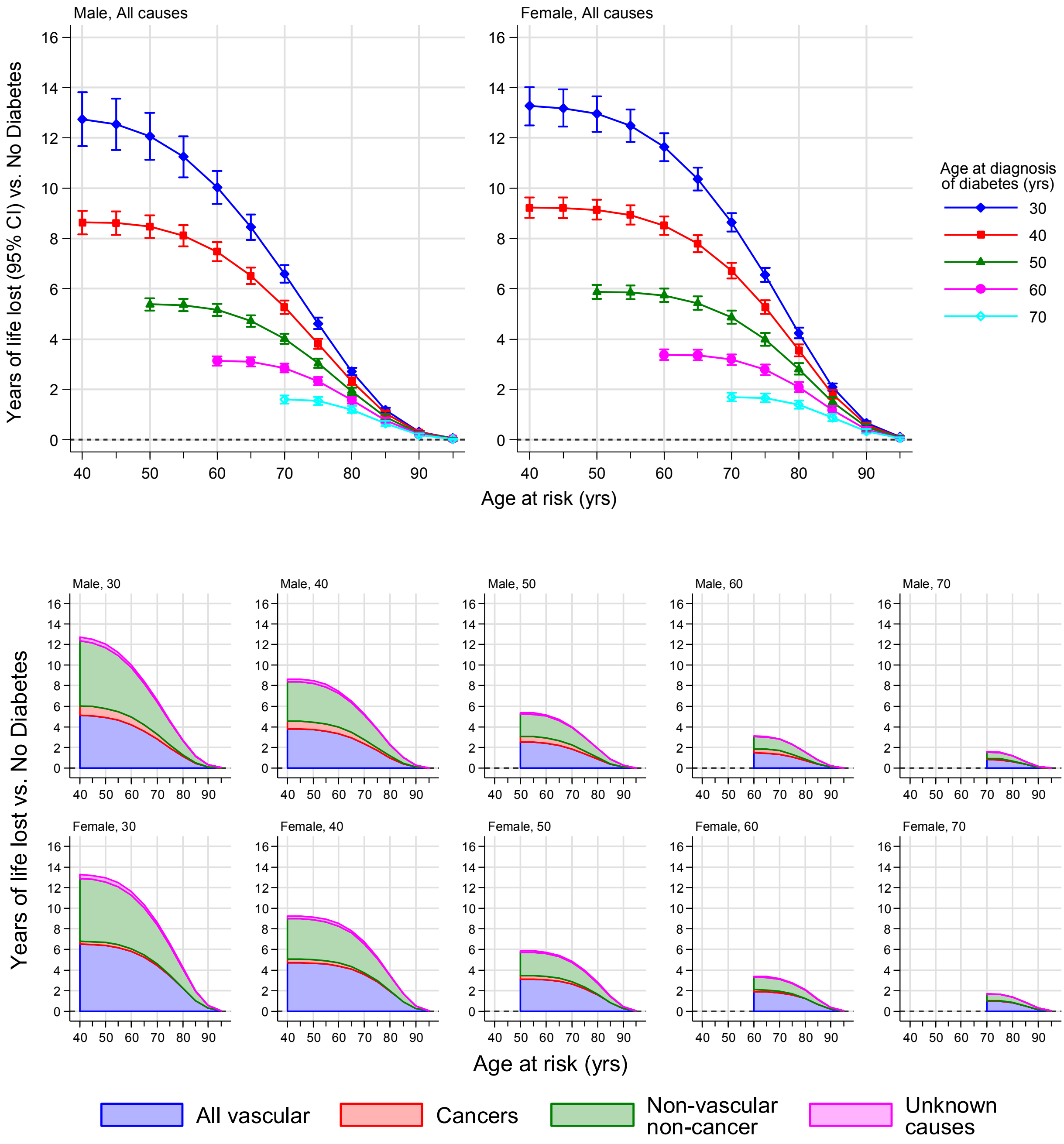

The estimates of cumulative survival from 40 years of age onwards according to age at diagnosis of diabetes were calculated by applying hazard ratios (specific to age at risk) for all-cause mortality associated with age at diagnosis of diabetes to EU 2015 death rates at the age of 40 years or older.

eFigure 9. Estimated cause-specific contributions to overall reduction in life expectancy by age at diagnosis of diabetes using US 2015 death rates, including diabetes diagnosed before age 30 years.

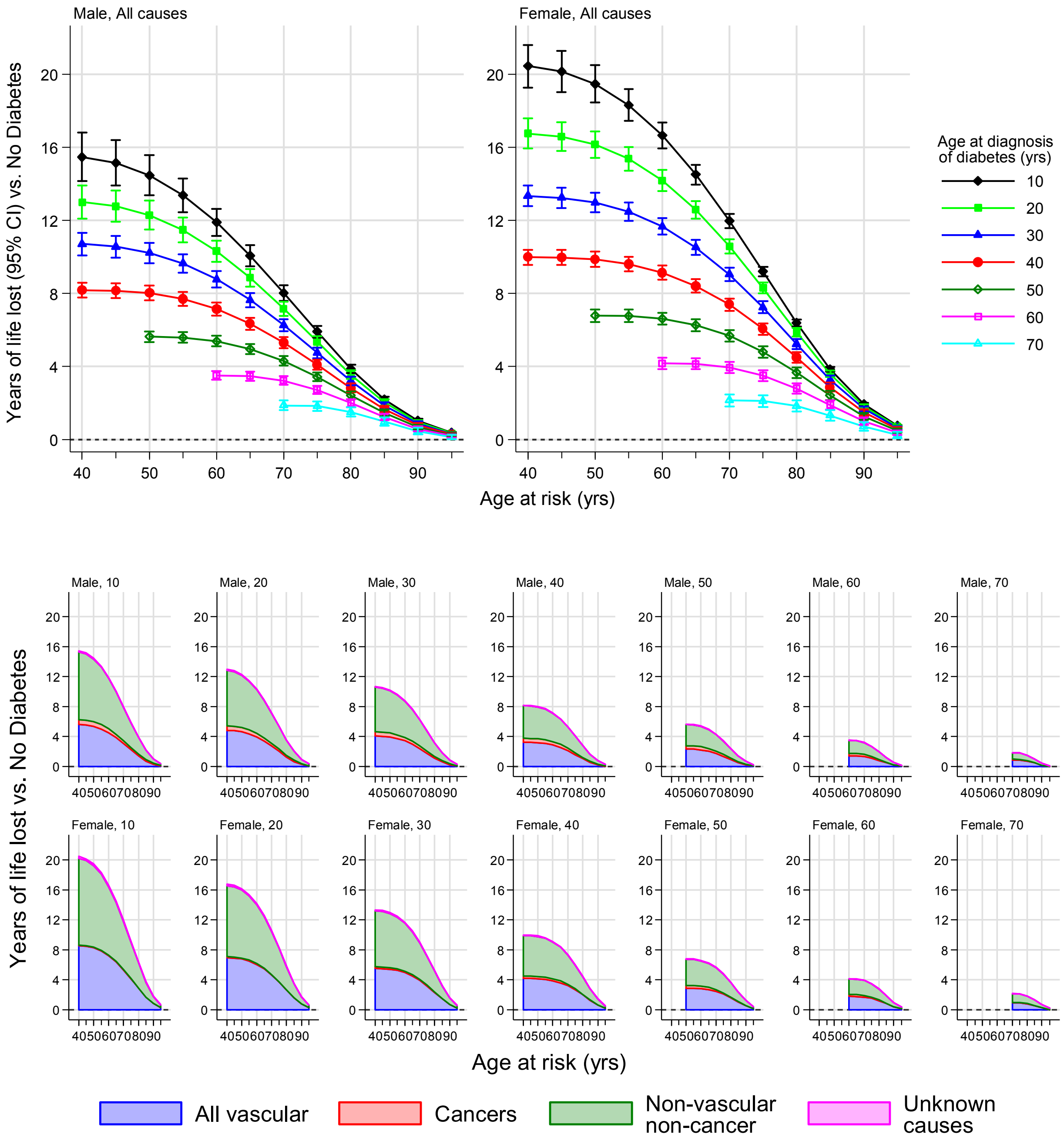

The estimates of cumulative survival from 40 years of age onwards according to age at diagnosis of diabetes were calculated by applying hazard ratios (specific to age at risk) for all-cause mortality associated with age at diagnosis of diabetes to US 2015 death rates at the age of 40 years or older.

eFigure 10. Estimated cause-specific contributions to overall reduction in life expectancy by age at diagnosis of diabetes using EU 2015 death rates, <u>including diabetes diagnosed before age 30 years</u>.

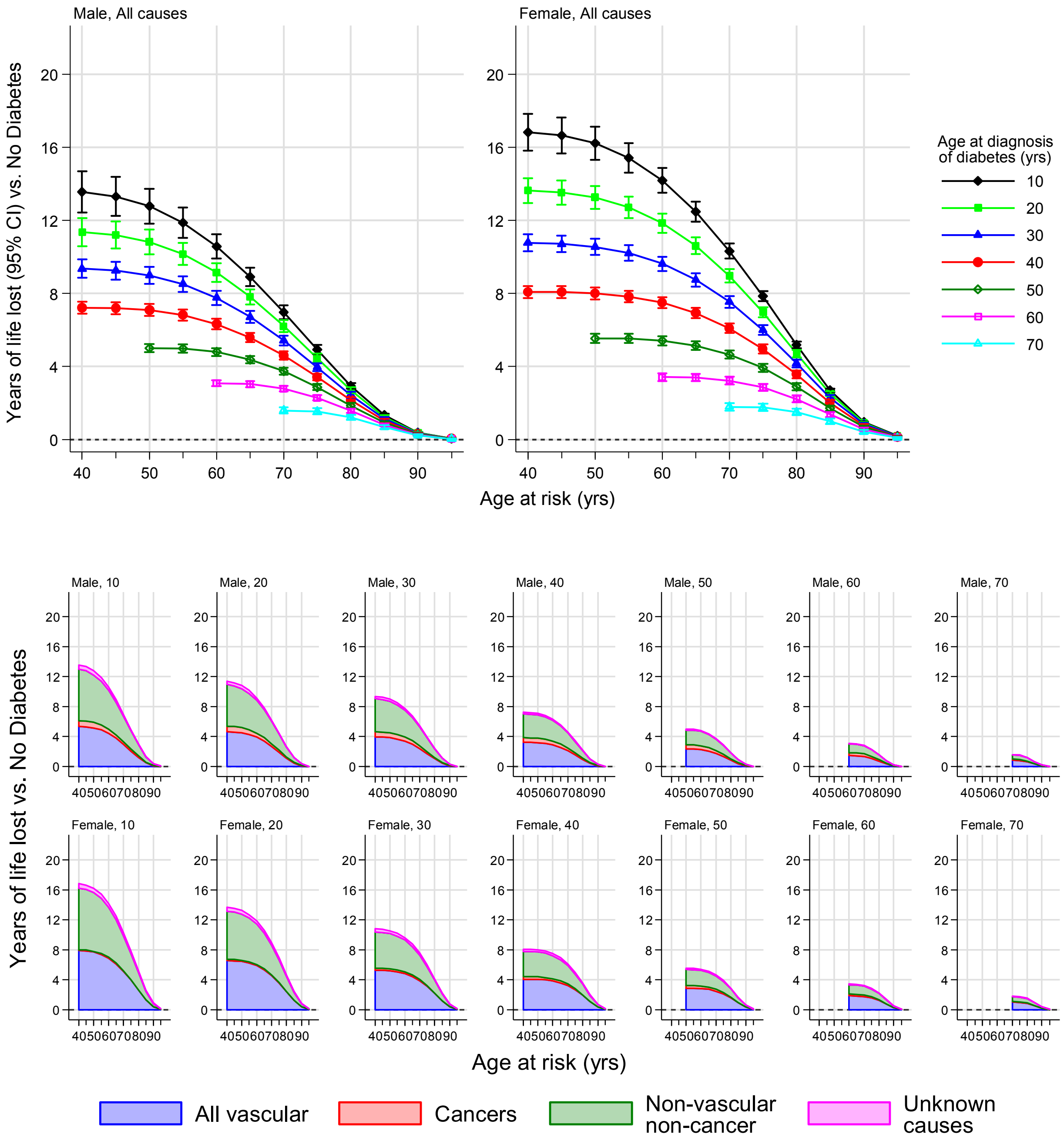

The estimates of cumulative survival from 40 years of age onwards according to age at diagnosis of diabetes were calculated by applying hazard ratios (specific to age at risk) for all-cause mortality associated with age at diagnosis of diabetes to EU 2015 death rates at the age of 40 years or older.

eAppendix 1. List of ERFC study acronyms*

Abbreviation	Full name
ARIC	Atherosclerosis Risk in Communities Study
AUSDIAB	Australian Diabetes, Obesity and Lifestyle Study
BHS	Busselton Health Study
BRUN	Bruneck Study
BWHHS	British Women's Heart and Health Study
CHARL	Charleston Heart Study
CHS	Cardiovascular Health Study
COPEN	Copenhagen City Heart Study
DESIR	Data from an Epidemiological Study on the Insulin Resistance Syndrome
DRECE	Diet and Risk of Cardiovascular Disease in Spain
EPESEIOW	Established Populations for the Epidemiologic Study of the Elderly Studies, Iowa
EPESENHA	Established Populations for the Epidemiologic Study of the Elderly Studies, New Haven
FINE_FIN	Finland, Italy and Netherlands Elderly Study
FINRISK92	Finrisk Cohort 1992
FRAMOFF	Framingham Offspring Cohort
GOTO13	Goteborg Study 1913
GOTOW	Population Study of Women in Göteborg, Sweden
HCS	Hertfordshire Cohort Study
HIMS	Health in Men Study
HOORN	Hoorn Study
HPFS1	Health Professionals Follow-up Study
LASA	Longitudinal Aging Study Amsterdam
MDC	Malmö Diert and Cancer Cardiovascular Study
MESA	Multi-Ethnic Study of Atherosclerosis
MONICA_KORA3	MONICA/KORA Augsburg Survey S3
NHANESI	National Health and Nutrition Examination Survey I
NHS1	Nurses' Health Study
PARIS1	Paris Prospective Study I
PREVEND	Prevention of Renal and Vascular End Stage Disease Study
QUEBEC	Quebec Cardiovascular Study
RANCHO	Rancho Bernardo Study
SHIP	Study of Health in Pomerani
SHS	Strong Heart Study
TOYAMA	Toyama Study
TROMSØ	Tromsø Study
ULSAM	Uppsala Longitudinal Study of Adult Men
WHS	Women's Health Study
ZUTE	Zutphen Elderly Study
ATENA	Progetto CUORE
BRHS1	British Regional Heart Study
EPICNOR	European Prospective Investigation of Cancer Norfolk Study
ESTHER	Epidemiologische Studie zu Chancen der Verhutung und optimierten Therapie chronischer Erkrankungen in der alteren Bevolkerung
FINNMARK	Cohort of Norway
FINRISK97	Finrisk Cohort 1997
FUNAGATA	The Funagata Study
GOTO33	Goteborg Study 1933
GOTO43	Goteborg Study 1943
HBS	Helsinki Businessmen Study
HISAYAMA	Hisayama Study
HUBRO	Cohort of Norway
KARELIA	North Karelia Project
MCVDRFP	Monitoring of CVD Risk Factors Project
MIDFAM	MIDSPAN Family Study
MORGEN	Dutch Monitoring Project on Risk Factors for Chronic Diseases
MOSWEGOT	MONICA Göteborg Study
MPP	Malmö Preventive Project
MRCOLD	MRC Study of Older People
NHANESIII	National Health and Nutrition Examination Survey III
NSHS	Nova Scotia Health Survey
OPPHED	Cohort of Norway
OSLO2	Cohort of Norway
RS_II	The Rotterdam Study II
RS_III	The Rotterdam Study III
TROMS	Cohort of Norway
USPHS2	U.S. Physicians Health Study II
WHIOS	Women's Health Initiative (Observational Study)
AFTCAPS	Air Force/Texas Coronary Atherosclerosis Prevention Study
CAPS	Caerphilly Prospective Study
EAS	Edinburgh Artery Study
EPESEBOS	Established Populations for the Epidemiologic Study of the Elderly Studies, East Boston
EPESENCA	Established Populations for the Epidemiologic Study of the Elderly Studies, North Carolina
FINE_IT	Finland, Italy and Netherlands Elderly Study
GOH	The Glucose Intolerance, Obesity and Hypertension Study
IKNS	Ikawa, Kyowa, and Noichi Study
ISRAEL	Israeli Ischaemic Heart Disease Study
KIHD	Kuopio Ischaemic Heart Disease Study
MATISS83	Progetto CUORE
MATISS87	Progetto CUORE
MIDCOLL	MIDSPAN Collaborative Study
MIDRP	MIDSPAN Renfrew & Paisley Study
MRFIT	Multiple Risk Factor Intervention Trial 1
NCS1	Norwegian Counties Study 1
NCS2	Norwegian Counties Study 2

NCS3	Norwegian Counties Study
NPHSII	Northwick Park Heart Study II
OSAKA	Osaka Study
PRHHP	Puerto Rico Heart Health Program
PROCAM	Prospective Cardiovascular Münster Study
PROSPER	Prospective Study of Pravastatin in the Elderly at Risk
REYK	Reykjavik Study
RS_I	The Rotterdam Study I
SPEED	Speedwell Study
TARFS	Turkish Adult Risk Factor Study
WCWC	Wuerttemberg Construction Workers Cohort
WHITEII	Whitehall II Study
WOSCOPS	West of Scotland Coronary Prevention Study

* The cohorts are listed in the same order as in eTable 1 for ease of cross-referencing.

eAppendix 2. UK Biobank

Details of UK Biobank have been described previously.[1] Briefly, over 500,000 participants aged 40-69 years were recruited during 2006-2010 in 22 geographical centres throughout the United Kingdom. The assessment visit comprised electronic signed consent; a self-completed touch-screen questionnaire; brief computer-assisted interview; physical and functional measures; and collection of biological samples. Participants have been linked with death records of the UK Office for National Statistics through National Health Service identification numbers.[2]

A total of 499,808 participants with complete data on age, sex, and medical history of diabetes, stroke and myocardial infarction were included in the current analysis. Medical history of diabetes, stroke and myocardial infarction was defined using self-reported information recorded at baseline visit in UK Biobank and updated using information on hospitalization before baseline extracted from Hospital Episode Statistics (using the following ICD codes: I21-I22 for history of myocardial infarction; I60, I61, I63, I64 for history of stroke). Information on smoking status, level of education (as measure of socioeconomic status), and fruit and meat consumption were collected using the touchscreen questionnaire at baseline visit. Body mass index was calculated (kg/m^2) using measured height and weight. Weight was measured using the Tanita BC-418 MA body composition analyser (accurate to within 0.1kg) after removal of heavy clothing and shoes. Standing height was measured without shoes using a Seca 202 height measure. Blood pressure was measured using the Omron HEM-7015IT digital blood pressure monitor.[1] The mean of two measurements taken approximately within a minute of each other was used in the analysis. The UK Biobank study was approved by the North West Multi-centre Research Ethics Committee (MREC) and all participants provided written informed consent to participate in the UK Biobank.

Statistical analysis

Hazard ratios and 95% CI for all-cause and cause-specific mortality were calculated using Cox proportional-hazards regression models. The primary analysis adjusted for age and sex only. Secondary analyses were additionally adjusted for body mass index, systolic blood pressure, medication usage (including lipid-lowering, anti-hypertensive, and diabetes drugs) and education level. Data were analysed using Stata version 13.1.

Reference List

(1) Sudlow C, Gallacher J, Allen N et al. UK biobank: an open access resource for identifying the causes of a wide range of complex diseases of middle and old age. PLoS Med 2015;12(3):e1001779.

(2) UK Biobank (2007). Protocol for a large-scale prospective epidemiological resource. http://www.ukbiobank.ac.uk/wp-content/uploads/2011/11/UK-Biobank-Protocol.pdf (accessed 06 March 2017).

eAppendix 3. Calculation of age at diagnosis of diabetes mellitus

To enable current analysis of associations according to age at diagnosis, prospective cohorts were asked to provide further data on age at diagnosis (or duration) of prevalent diabetes and also, where available, information on incident diabetes (provided either as known age/date/time of diagnosis, or participant's diabetes status at date-stamped resurveys). Where information was provided as time duration since/prior to baseline, we calculated the age at diagnosis as age at baseline plus/minus the time duration. Where incidence information was provided as diabetes status (yes/no) at date-stamped resurveys, we estimated the age at diagnosis as the participant's age at the midpoint of two consecutive surveys in which the participant developed diabetes. We also computed an accuracy indicator as half-width of the time interval between the two surveys.

The age at diagnosis of diabetes could either have been reported at a baseline survey (i.e. prevalent diabetes) or corresponded to new diagnosis of diabetes during follow up (i.e. incident diabetes). Thus the 96 ERFC cohorts that met this criteria, could be broadly classified into three groups (**eTable 1**): (i) those that recorded both age at diagnosis of prevalent diabetes and incident diabetes (36 cohorts, 374,136 participants); (ii) those that enquired on age of diagnosis of prevalent diabetes and did not ascertain incident diabetes (29 cohorts, 300,080 participants); and (iii) those that did not enquire on age of diagnosis of prevalent diabetes but ascertained incident diabetes (31 cohorts, 234,369 participants).

eAppendix 4. Statistical methods used for estimating years of life lost

We used three pieces of information to estimate reductions in life expectancy associated with diagnosis of diabetes at a specified age (henceforth "age at diagnosis"):

(i) age-at-risk specific hazard ratios for all-cause (and cause-specific) mortality for specified ages at diagnosis of diabetes compared to those without diabetes (derived from the ERFC);

(ii) population all-cause (and cause-specific) mortality rates (derived from the detailed mortality component of the CDC WONDER database of the US Centers for Disease Control and Prevention); and

(iii) age-at-risk specific prevalence of diabetes in the population by age at diagnosis (derived from the ERFC).

We utilised age-at-risk specific hazard ratios for mortality by age at diagnosis of diabetes, estimated from ERFC data, and published routine statistics on overall population mortality rates to estimate population survival curves by age at diagnosis of diabetes. To calculate an appropriate mortality rate for the reference group (i.e. those without diabetes at a given age-at-risk) we modelled age-at-risk specific prevalence of diabetes by age at diagnosis in ERFC data as described below. We estimated reductions in life-expectancy as differences in areas under any two survival curves compared.

Age-at-risk specific hazard ratios for mortality by age at diagnosis of diabetes were estimated from ERFC data separately for each sex. Specifically, a Cox regression model stratified by cohort and trial arm (where applicable) was fitted separately for each sex using a dataset in which participant ages-at-risk were deterministically updated by splitting the follow up times every 5-years and recalculating an age-at-risk variable at the beginning of each 5-year interval of follow up. Interactions between time-dependent variables of diabetes status (yes/no) and age at diagnosis with linear and quadratic terms for the age-at-risk variable were included in the model to obtain smoothed hazard ratios for specified ages at diagnosis of diabetes. Thus, for participant i in stratum s the log hazard rate at time t since baseline was modelled as:

$$\begin{aligned}\log(h_{si}(t)) = \log(h_{s0}(t)) + \beta_0 diab_{si} + \beta_1 agediab_{si} + \beta_2 agerisk_{si} + \beta_3 agerisk_{si}^2 + \\ \beta_4 diab_{si} \times agerisk_{si} + \beta_5 diab_{si} \times agerisk_{si}^2 + \\ \beta_6 agediab_{si} \times agerisk_{si} + \beta_7 agediab_{si} \times agerisk_{si}^2\end{aligned} \quad (1)$$

from which the age-at-risk specific hazard ratios (and 95% CIs) for mortality at specified ages at diagnosis of diabetes (30, 40, 50, 60, and 70 years) were obtained as linear combinations of the relevant estimated coefficients, with age-at-risk fixed at values corresponding to midpoints of 5-year age-groups from age 40 onwards (**Figure 1**).

Population all-cause (and cause-specific) mortality rates per 100,000 were obtained in 5-year age-groups for the US population during year 2015 from the Center for Disease Control (CDC) WONDER online database (https://wonder.cdc.gov/ucd-icd10.html) (**Figure2**), as well as for 28 EU countries during year 2015 (http://ec.europa.eu/eurostat/data/database). Because the mortality rates were provided only up to age-group 80-84 years and the open-ended interval of ≥85 years, but we desired to estimate the overall population survival curves, we used a piecewise cubic Hermite interpolation (PCHIP) method to smooth through the midpoints of 5-year age-groups and extrapolate the mortality rates to age 110 years (**Figure 3**). Next, assuming exponential survival (i.e. constant hazard) within each 5-year age group, we estimated the age-specific survival probability as $S_a = \exp(-5 \times IR_a)$ and derived the overall population survival curves from age 35 onwards as the product of the relevant age-group specific survival probabilities (**Figure 4**).

$$p(survival \mid agerisk \geq 35) = \prod_{agerisk \geq 35} S_a \quad (2)$$

In order to infer population mortality rates appropriate for the reference group used in our estimation of age-specific hazard ratios (i.e. those without diabetes at a given age), we used logistic regression to model the age-at-risk specific prevalence of diabetes by age at diagnosis in ERFC cohorts by sex and decade of follow up (**Figure 5**) We used the

age-specific prevalence estimates for the decade commencing in the year 2000 to infer the age-specific mortality rates appropriate for the reference group without diabetes IR_{a0} as:[1]

$$IR_{a0} = \frac{IR_a}{p_{a0} + \sum_{j=1}^{5} p_{aj} \times RR_{aj}} \qquad (3)$$

Where IR_a is the population mortality rate for age group a, p_{aj} is the age-specific prevalence of 5 groups of diabetes age at diagnosis j, and RR_{aj} is the age-specific hazard ratio for comparison of group j versus reference group ($j = 0$). The age-specific mortality rates in each of the non-reference age at diagnosis groups were then inferred in turn by multiplying the age-specific mortality rate for the reference group IR_{a0} by the age-specific hazard ratios RR_{aj} based on ERFC data, and equation (2) above used to infer the age at diagnosis-specific population survival curves (**Figure 6**). Finally, reductions in life expectancy according to age at diagnosis were estimated as difference in the areas under the survival curves for the reference group and each age at diagnosis in turn (**Figure 7**). The areas under curves were calculated by numerical integration.

eAppendix 4 References

1 Woloshin S, Schwartz LM, Welch HG. The risk of death by age, sex, and smoking status in the United States: putting health risks in context. J Natl Cancer Inst 2008;100(12):845-53.

eAppendix 4. Figures

Figure 1. Age-at-risk specific hazard ratios for all-cause mortality by sex and age at diagnosis of diabetes

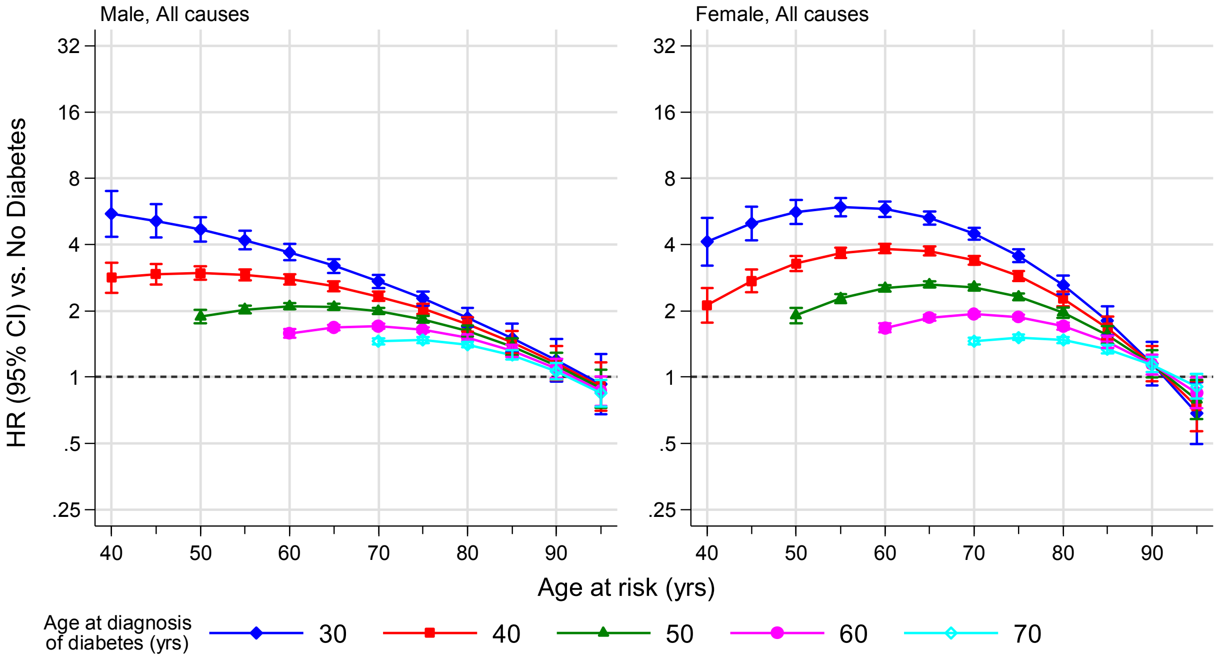

Figure 2. US population mortality rates during year 2015 downloaded from CDC WONDER online database*

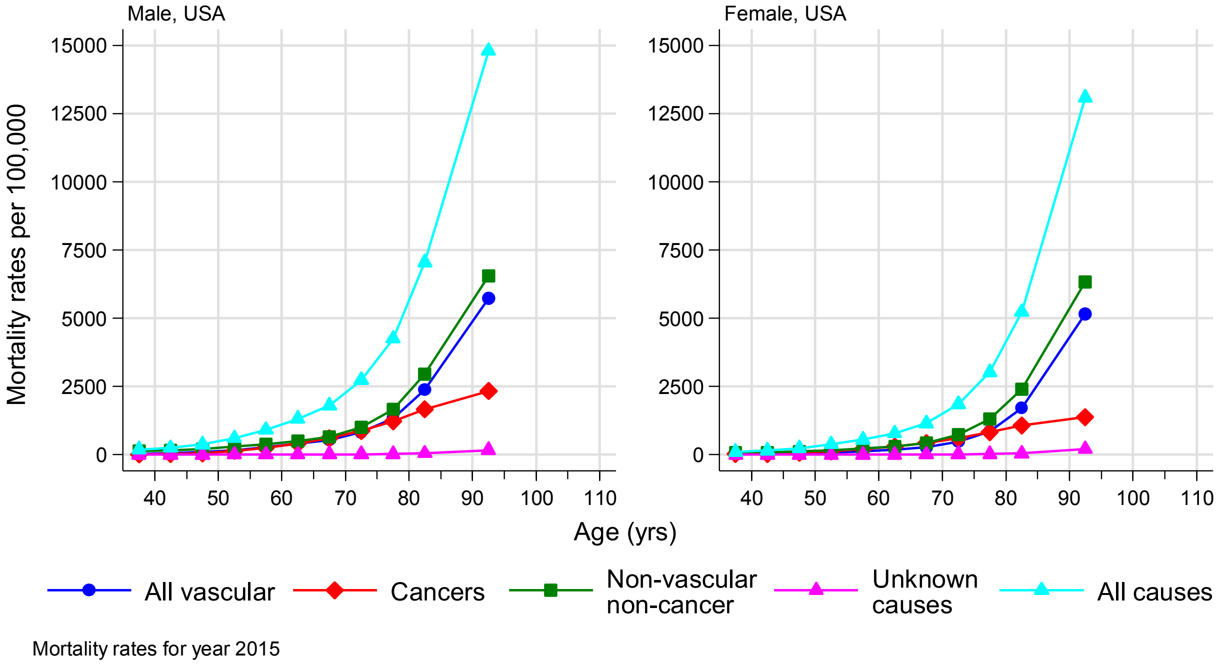

* To maintain consistency with analyses conducted in ERFC, the mortality rate for non-vascular non-cancer causes was recalculated as the difference of all-cause mortality and the sum of vascular mortality (I00-I99), cancer mortality (C00-D48), and unknown causes of mortality (R00-R99).

Figure 3. Assessment of adequacy of a piecewise cubic Hermite interpolation (PCHIP) method to smooth and extrapolate US population mortality rates during year 2015. Data was downloaded from CDC WONDER online database in 5-year age groups up to 80 years inclusive, and the unbounded 85 years plus category (the database's highest age group category with mortality rates estimates provided).

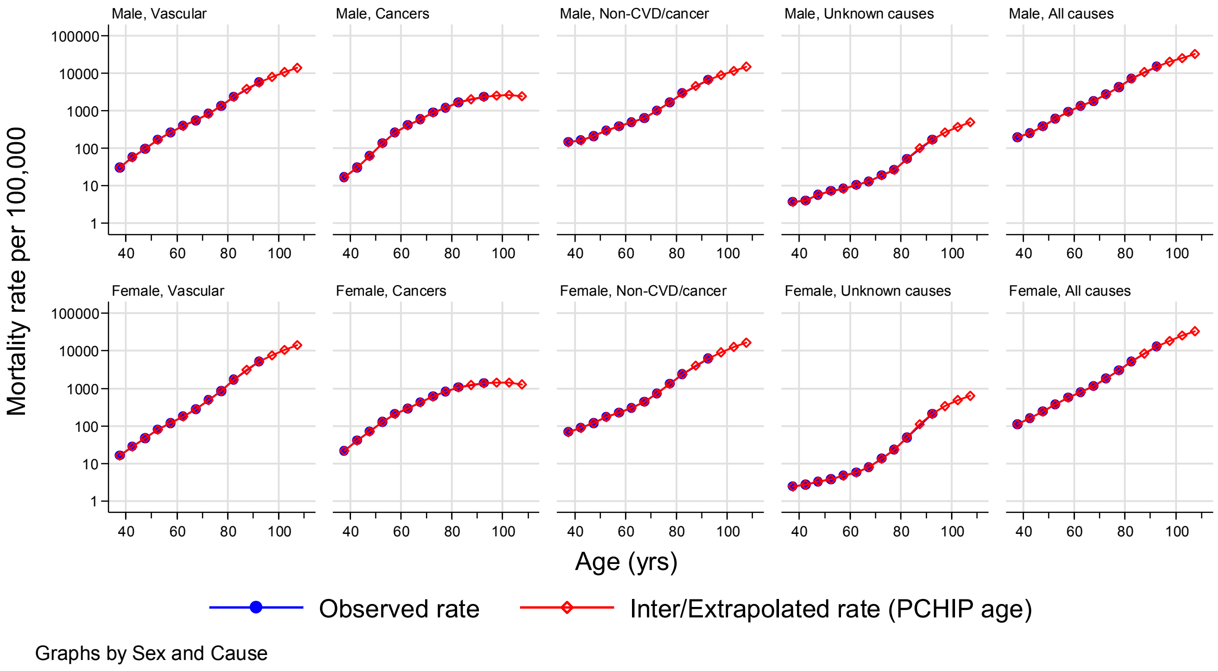

Figure 4. Derived population survival curves for all-cause and cause-specific mortality from age 35 years based on smoothed and extrapolated US population mortality rates for year 2015

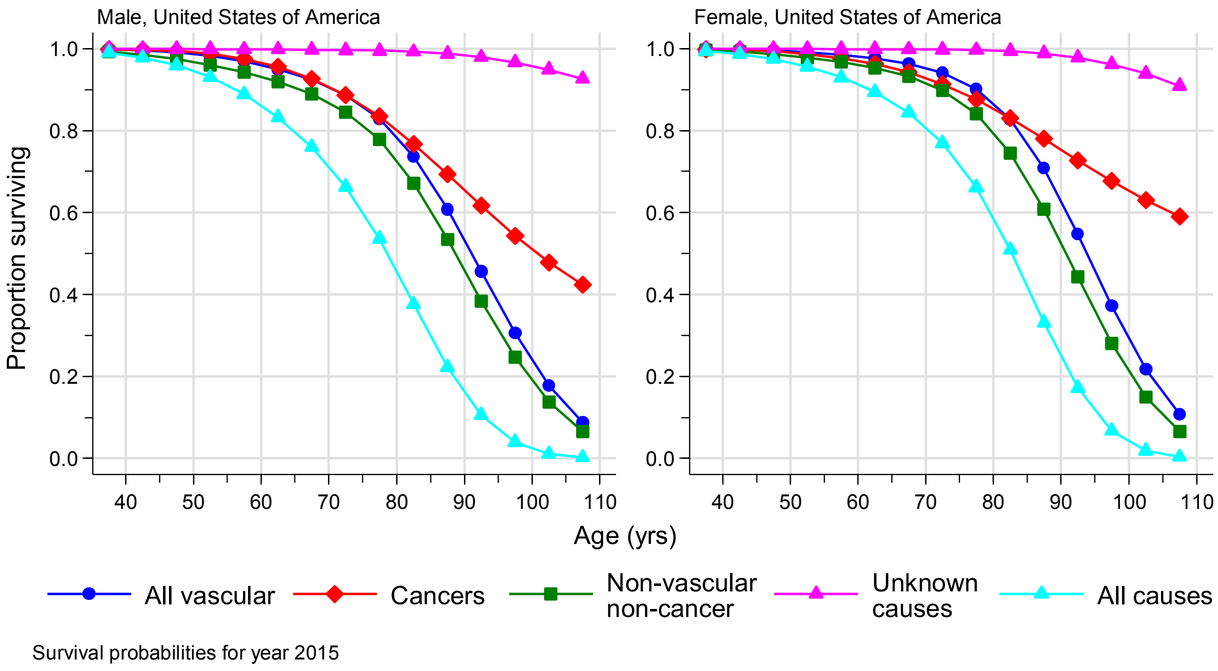

Figure 5. Modelled age-specific prevalence of diabetes in ERFC cohorts by sex and decade of recruitment and age at diagnosis.

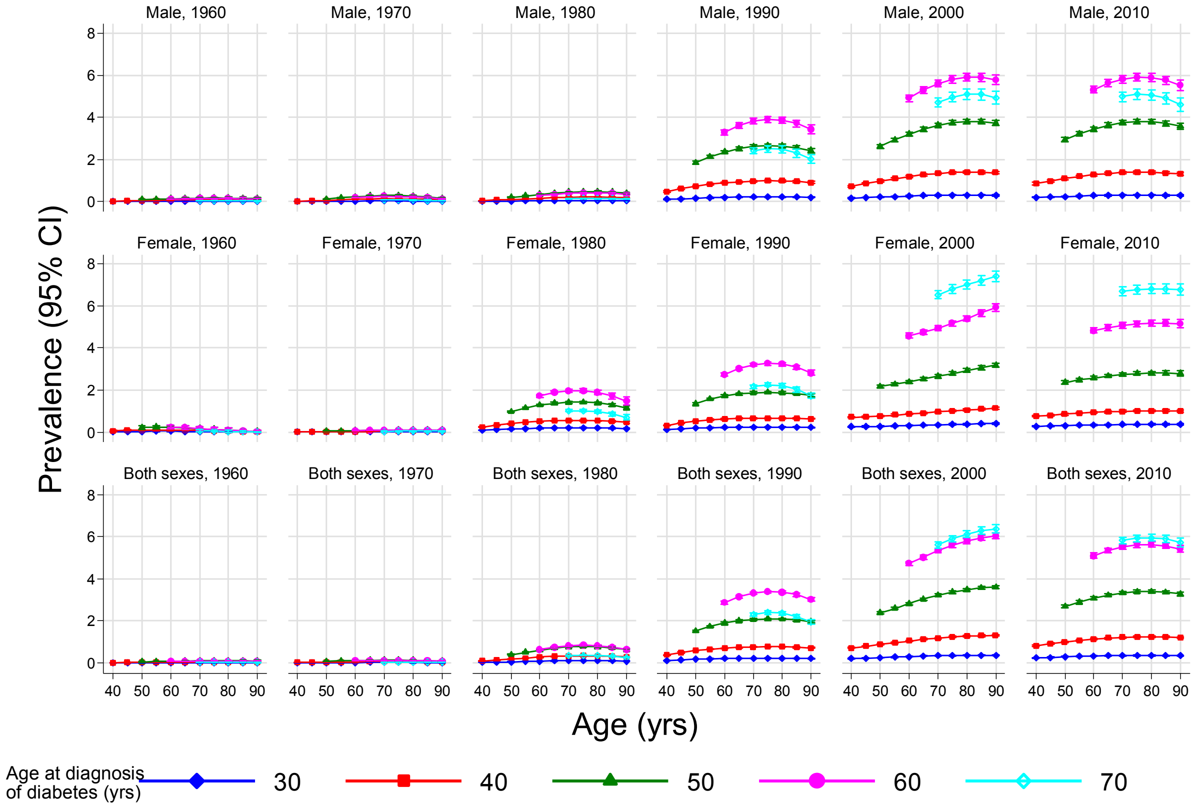

Figure 6. Inferred survival curves for US population by sex and age at diagnosis of diabetes

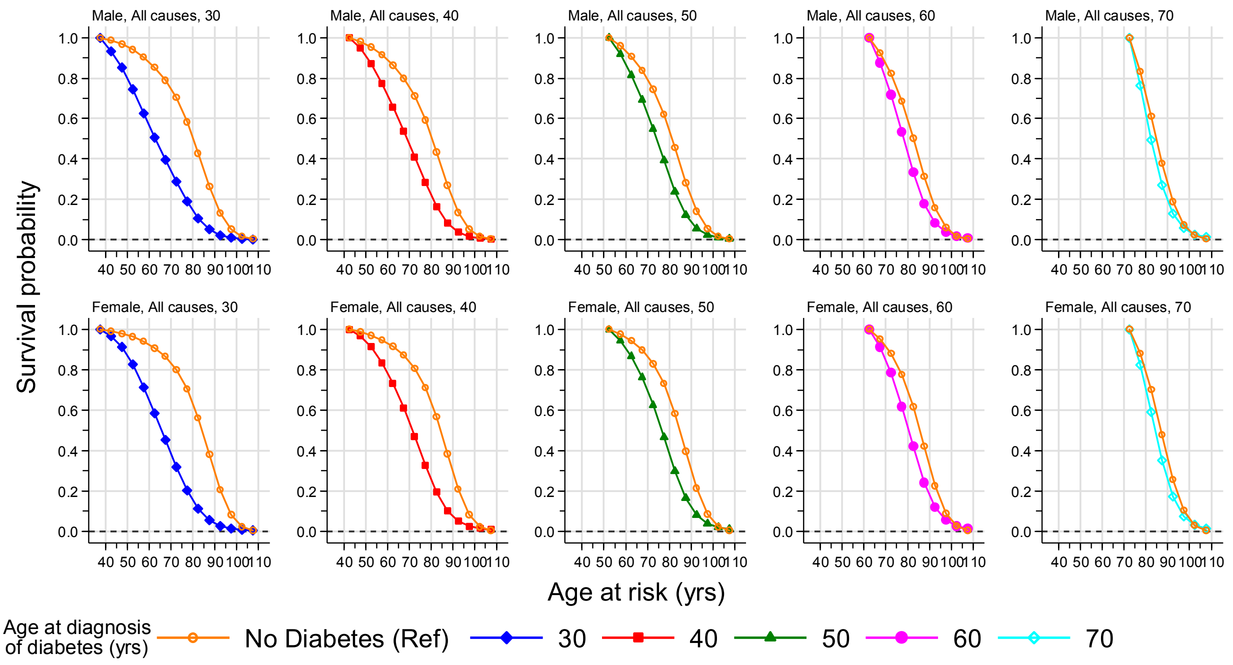

Figure 7. Estimated sex-specific reductions in life expectancy in the US population according to age at diagnosis of diabetes

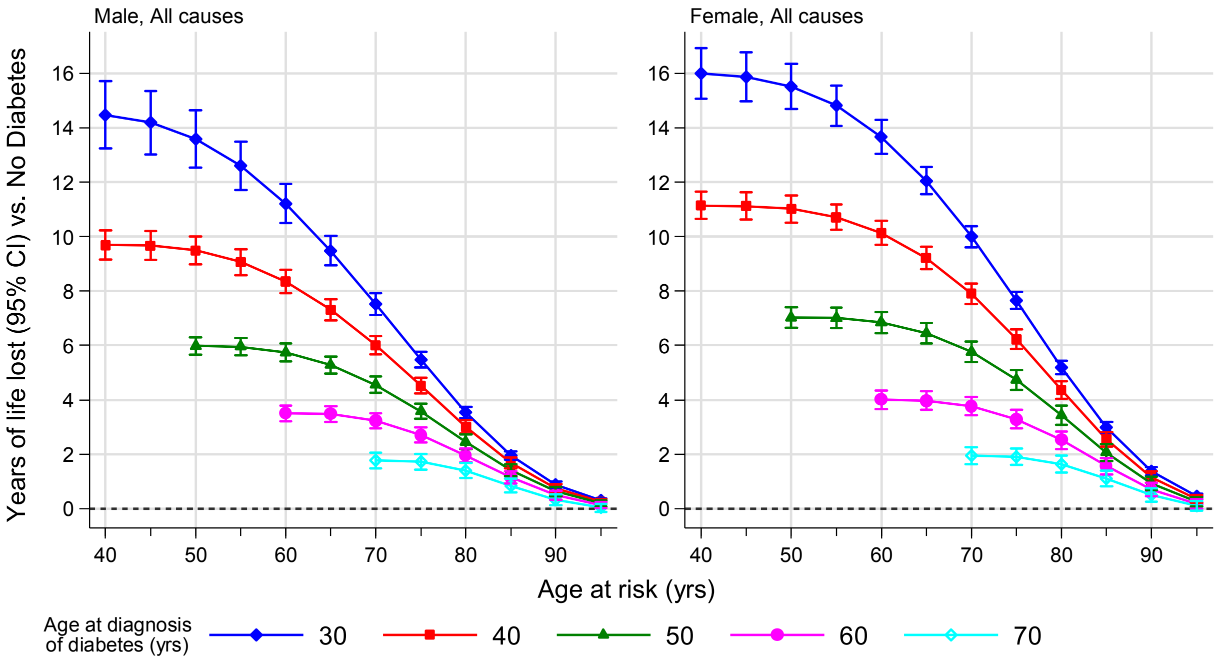

Supplement: Supplementary material [file EMS189036-supplement-Supplementary_material.pdf]
